# Therapeutic Efficacy of Carvacrol-Loaded Mesoporous Silicate Nanoparticles Against Cryptosporidiosis

**DOI:** 10.3390/pharmaceutics18091065

**Published:** 2026-08-26

**Authors:** Moataz M. Rashad, Shaimaa M. Kasem, Khaled E. El-Kelany, Sara A. Abdel Gaber

**Affiliations:** 1 Institute of Nanoscience and Nanotechnology, Kafrelsheikh University, Kafrelsheikh 33516, Kafr El‑Sheikh, Egypt; 2 Nanomedicine Department, Institute of Nanoscience and Nanotechnology, Kafrelsheikh University, Kafrelsheikh 33516, Kafr El‑Sheikh, Egypt; 3 Zoology Department, Faculty of Science, Kafrelsheikh University, Kafrelsheikh 33516, Kafr El‑Sheikh, Egypt; 4 Chemistry Department, Faculty of Science, University of Sadat City, Sadat City 32897, Monufia, Egypt; 5 Clinical Pharmacy Department, Faculty of Pharmacy, Egypt-Japan University of Science and Technology, New Borg El-Arab 21934, Alexandria, Egypt

**Keywords:** carvacrol, mesoporous silicate nanoparticles, cryptosporidiosis, anti-inflammatory, antioxidant

## Abstract

**Purpose:** Cryptosporidiosis is a parasitic inflammatory disease that causes the death of around 1.6 million people annually worldwide and is associated with *Cryptosporidium parvum* (*C. parvum*) infection. This study aimed to evaluate the therapeutic efficacy of carvacrol (CV) loaded into mesoporous silicate nanoparticles (MSNs) against *C. parvum* using in vitro, in vivo, histopathological, immunohistochemical, biochemical, and computational approaches. **Methods:** In vitro assays were conducted to evaluate the oocysticidal activity of CV-loaded MSNs (CV-MSNs) for 96 h. In silico assays were carried out to investigate target inflammatory proteins involved in cryptosporidiosis. In vivo tests were performed on male Swiss albino mice (22 ± 5 g, 4–6 weeks) to assess the anti-inflammatory and antioxidant effects of CV after loading onto MSNs. **Results:** CV showed its highest oocysticidal efficacy after loading onto spherical 72 nm MSNs (MSN2), exhibiting a 0.07 mg/mL LC_50_. At the in vivo level, CV-MSN2 markedly restored ileal, pulmonary, and hepatic histoarchitecture and normalized biochemical indices in infected mice. CV-MSN2 also showed the strongest inhibitory action against computationally selected target proteins, TLR-4, NF-κb-P65, STAT-3, NOS2, and JAK-1, with accompanying downregulation of IL-6, IL-1β, and TNF-α. Similarly, antioxidant markers were significantly decreased following CV-MSN2 treatment compared to the positive controls. **Conclusions:** This study shows that MSNs enhanced CV efficacy against ileal inflammation induced by *C*. *parvum*, preserving CV’s molecular targets.

## 1. Introduction

Cryptosporidiosis is an intestinal inflammatory disease caused by infection with *C. parvum* in contaminated food and water [1]. It causes about 1.6 million deaths annually worldwide, especially among children and immunocompromised and diabetic patients, according to the World Health Organization (WHO) [2]. More than 7.5 million cases and 200,000 deaths are recorded annually in children aged 0 to 24 months in sub-Saharan Africa and Asia due to cryptosporidiosis [3]. After ingestion, sporulated *Cryptosporidium* oocysts mainly invade the ileum epithelium and ectopically invade the liver and lungs [4]. The released sporozoites adhere to the apical microvillus membrane of the intestinal epithelium [5]. During this time, the attached sporozoites can be identified by toll-like receptor 4 (TLR4) expressed on immune cells, including macrophages, dendritic cells, and intestinal epithelial cells (IECs) via pathogen-associated molecular patterns (PAMPs) [5,6].

Early TLR4 activation triggers rapid nuclear translocation of nuclear factor-κB p65 (NF-κB p65) within approximately 2 h post-infection, resulting in a primary mild upregulation of key pro-inflammatory cytokines, including tumor necrosis factor-α (TNF-α), interleukin-6 (IL-6), and interleukin-1β (IL-1β) [7,8]. This reaction initiates a cascade of inflammatory signaling pathways that may lead to persistent chronic intestinal, hepatic, or pulmonary damage. Following TLR4 recognition, the activation of nuclear factor-κB p65 (NF-κB p65) results in the overexpression of inducible nitric oxide synthase (iNOS), which leads to severe injury due to peroxynitrite secretion (ONOO^−^) [9,10]. This increases the level of malondialdehyde (MDA), which increases as a consequence of the proposed lipid peroxidation that suppresses the release of antioxidants, including superoxide dismutase (SOD), catalase (CAT), and glutathione (GSH) [10,11]. At a later stage, the Janus kinase-1 (JAK-1) signal transducer and activator of transcription-3 (STAT-3) pathway is activated to ensure sustained chronic inflammation by maintaining cytokine production and reinforcing NF-κB activation [12]. p38 mitogen-activated protein kinase (p38-MAPK) can also be activated by pro-inflammatory cytokines such as tumor necrosis factor-α (TNF-α), interleukin-6 (IL-6), and interleukin-1β (IL-1β) to upregulate the secretion of NF-κB p65 [13].

Nitazoxanide (NTZ) is currently the only FDA-approved drug for the treatment of cryptosporidiosis. However, its therapeutic efficacy is limited, particularly in immunocompetent patients, and concerns remain regarding its effectiveness in young children due to complicated inflammatory reactions. Consequently, increasing attention has been directed toward natural phenolic compounds as promising alternative therapeutic agents. These compounds include carvacrol (CV), gallic acid, caffeic acid, quercetin, rutin, tannic acid, and curcumin.

CV is a naturally occurring monoterpene, predominantly found in the essential oils of thyme and oregano species. Its antioxidant activity is primarily attributed to the scavenging of reactive oxygen species (ROS) through its phenolic hydroxyl group that is stabilized by resonance within the hydrophobic aromatic ring [14,15]. In addition, CV exhibits several favorable biological properties, including high membrane permeability, excellent biocompatibility, and broad-spectrum antimicrobial activity [16,17]. Compared with many other phenolic compounds, CV has demonstrated potent anti-parasitic activity owing to its strong membrane-disruptive effect, low molecular weight, multi-target mechanism of action, and favorable physiological stability [18]. Nevertheless, the biomedical application of CV is hindered by several physicochemical limitations, including poor aqueous solubility, high volatility, and low bioavailability [17,19].

Several studies have reported its anti-cryptosporidial activity despite significant research gaps remaining uncovered. One study demonstrated that the oocysticidal efficacy of thyme extract is about 3–30% of CV, up to ≈45%, using *C. parvum* infectivity in vitro in HCT-8 cells. Nevertheless, this investigation did not explore the antioxidant or anti-inflammatory mechanisms in depth and ignored the role of sporozoites in virulence occurrence [20]. The second in vitro study focused on the analysis of its anti-cryptosporidial effects via oxidative stress induction, phospholipase inhibition, and calcium signaling modulation, yet its precise molecular mechanism remains poorly defined [21]. Another in vivo study largely concentrated on evaluating the anti-cryptosporidial activity of thyme oil and other essential oils like mint, chamomile, and basil using parasitological, histopathological, serological, and biochemical analyses [22]. In silico studies were limited and depended on molecular docking and dynamics, in addition to network pharmacology analyses, to assess the anti-inflammatory activity of CV and oregano essential oils toward target proteins like TNF and caspase-3, key regulators of apoptosis and neuro-inflammation [23,24,25].

Despite the promising therapeutic potential of CV, a critical gap remains unaddressed in overcoming its physicochemical limitations, which restrict its anti-cryptosporidial, anti-inflammatory, and antioxidant efficacy. Although several previous studies have investigated the anti-parasitic activity of thyme extracts, these extracts contain complex mixtures of essential oil constituents, making it difficult to attribute the observed biological effects to CV. Moreover, the crosstalk between inflammatory signaling pathways and antioxidant defense mechanisms during *Cryptosporidium* infection, as well as the therapeutic impact of pure CV on these interconnected processes, has not been comprehensively investigated. Addressing these research gaps is essential for the rational development of effective CV-based therapeutic strategies against cryptosporidiosis.

Thus, this study aims to provide a comprehensive in vitro, in silico, and in vivo study to evaluate the anti-inflammatory effects of free CV and CV loaded onto mesoporous silicate nanoparticles (MSNs) with optimized shapes and sizes. Cryptosporidiosis was used as an understudied model for chronic inflammation induced by the *C. parvum* parasite to study the molecular mechanisms of inflammatory pathways during the infection and after CV-based treatments. Using an integrative approach combining experimental studies with network pharmacology and computational chemistry, this study provides a mechanistic insight into how nanocarrier-mediated CV delivery reshapes host inflammatory responses while simultaneously enhancing anti-oocysticidal efficacy.

## 2. Materials and Methods

### 2.1. Materials

Tetraethyl orthosilicate (TEOS) (Mw = 208.33 g/mol) and 3-(triaminopropyl)-triethoxysilane (APTES) (Mw = 221.37 g/mol) were purchased from Merck KGaA, Darmstadt, Germany. Carvacrol (CV, 99% purity, food grade) was obtained from Merck (Darmstadt, Germany) and used without further purification. Toluene and Trimethylammonium bromide (CTAB) were provided by Sigma-Aldrich, Taufkirchen, Germany. Serum glutamic pyruvic transaminase (SGPT) and serum glutamic oxaloacetic transaminase (SGOT) kits were obtained from Sant Esteve de Bas, Girona, Spain. Urea and Creatinine kits were purchased from Diamond Diagnostics (Holliston, MA, USA). iNOS (cat #2982S), NF-ĸB-p65 (cat #6956S), JAK-1 (cat #3344S), STAT-3 (cat #9139), TLR4 (cat #14358), and p38-MAPK primary antibodies (cat #9217) (1:100 dilution) were provided by Cell Signaling Technology (Danvers, MA, USA). Horseradish peroxidase (HRP)-loaded goat anti-rabbit secondary antibody (cat #10004301) and diaminobenzidine (DAB) were supplied by Cayman Chemical Company, Ann Arbor, MI, USA, and Sigma-Aldrich, Taufkirchen, Germany, respectively. Murine ELISA kits of TNF-α, IL-6, and IL-1β cytokines were purchased from Thermo Fisher Scientific, Waltham, MA, USA. Direct-zol RNA Miniprep Plus (cat #R2072, Zymo Research Corp., Irvine, CA, USA), SuperScript IV One-Step RT-PCR kit/SYBER green master mix (cat #12594100, Thermo Fisher Scientific, Waltham, MA, USA), and primer sequences (cat #12594100, Thermo Fisher Scientific, Waltham, MA USA) were utilized in qRT-PCR analysis. All reagents were of analytical grade and used without further purification. Double distilled water was used in the experiments.

### 2.2. Synthesis of Amino (NH_2_)-Functionalized Mesoporous Silicate Nanoparticles (MSNs)

In this study, three MSNs were synthesized, labeled as MSN1, MSN2, and MSN3. MSN1 was prepared by dissolving 80 mg of CTAB in 40 mL of a deionized water and ethanol mixture (31.5:1, H_2_O: EtOH, V:V). After stirring at 800 rpm, 500 µL of TEOS and ammonia (35%) were added. Stirring at 25 °C continued for 3 h at 800 rpm, and centrifugation was conducted at 14,000 rpm for 1 h. A white precipitate was obtained and washed three times with deionized water. Drying took place in two steps: the first was at 100 °C in a drying oven (BINDER GmbH, Tuttlingen, Germany), and the second was by calcination in a calcination muffle furnace (Nabertherm GmbH, Lilienthal, Germany) at 600 °C for 3 h. MSN2 and MSN3 were prepared by dissolving 200 mg of CTAB in 96 mL of deionized water. NaOH (700 µL of 2 M) was added while stirring at 100 °C. TEOS (1400 µL) was added directly in the case of MSN2 and in a dropwise addition over 1 h in the case of MSN3. The mixture was stirred at 900 rpm at 100 °C for 2 h and left to cool. The white precipitate was obtained by centrifugation at 12,181× *g* for 1 h, followed by washing three times with deionized water. The obtained precipitate was dried in a drying oven (BINDER GmbH, Tuttlingen, Germany) at 100 °C. Calcination was performed in the muffle furnace (Nabertherm GmbH, Lilienthal, Germany) at 400 °C for 3 h [26].

For amino-functionalization, calcinated MSNs (50 mg) were suspended in 10 mL of toluene and 700 µL of APTES at 120 °C, at reflux for 3 h. Centrifugation was then performed at 1218× *g* for 1 h, followed by washing 3 times with ethanol and deionized water. The functionalized MSNs were dried at 100 °C in the drying oven (BINDER GmbH, Tuttlingen, Germany).

### 2.3. Carvacrol (CV) Loading with MSNs

A solution of CV was prepared at a concentration of 0.5 mg/mL using (H_2_O:EtOH, 80:20, V:V) as the solvent. CV solution was added in a dropwise manner into the MSN solution (2 mg/mL, H_2_O) in a mass ratio (CV:MSN, 1:4) with gentle stirring overnight at RT. This mass ratio was selected based on preliminary optimization experiments performed prior to this study. Centrifugation was then performed at 12,181× *g* for 1 h, followed by washing three times. Supernatants were collected to be used for quantifying CV loading. The CV-loaded MSNs were dried at 40 °C for 3 h.

### 2.4. Characterization Techniques

Transmission electron microscopy (TEM) was conducted at 200 KV using JEOL, JEM-200 Tokyo, Japan. Scanning electron microscopy (SEM) was carried out with a field-emission SEM (FEG, Thermo Scientific, Quattro S, NL Breda, The Netherlands) at 10 KV. Particle size distribution histograms were drawn based on measurements of 50 nanoparticles (NPs) after setting the scale bar using ImageJ software, version 1.54 (National Institutes of Health (NIH), Bethesda, MD, USA). Elemental analysis and distribution were determined using a SEM silicon-drift EDS detector (SEM, JSM IT-100, JEOL, Tokyo, Japan) with an energy resolution of about 129 eV, maintaining the analysis conditions as WD of 10 mm and a voltage up to 20 KV. Samples were coated with gold for SEM examination using sputter coating (DIL-29030SCTR, JEOL Smart Coater, Japan Ltd., Tokyo, Japan). Surface functionalization was evaluated using a Fourier transform infrared (FTIR) spectrometer (Japan, FTIR 6800 JASCO Corporation, Tokyo, Japan). X-ray diffraction (XRD) was measured using an X-ray detector (XRD 6000, Shimadzu Corporation, Kyoto, Japan). Hydrodynamic size and zeta potential were analyzed with a Nano-ZS (Brookhaven Instruments Corporation, Holtsville, NY, USA).

### 2.5. Calculation of CV Loading Efficiency (L.E.%)

The concentration of free CV in the collected supernatants was measured using a UV–vis spectrophotometer (Shimadzu Corporation, Kyoto, Japan) at a λmax of 273 nm. A calibration curve of the known CV concentration was plotted to determine the concentration of free CV corresponding to the detected absorbance. The L.E.% was calculated using the following equation [27]:

$$L.E.(\%)=\frac{Total\;CV\;Conc.-Free\;CV\;Conc.\;in\;Supernatant}{Total\;CV\;Conc.}$$

### 2.6. Evaluation of CV Release Profile

The release profile experiment was performed to evaluate the release behavior of CV-MSNs in an environment representative of the intended in vivo application. Release kinetics were determined in a mixture of normal saline and ethanol (80:20, V:V) at pH 7.4 at 37 °C ± 0.5 °C under continuous agitation. The release medium was chosen to maintain sink conditions and ensure adequate solubility of CV during the release study, while the pH and temperature were maintained close to physiological values. CV-MSN suspensions were stirred at RT. Samples (1 mL) were withdrawn at different time intervals over 72 h, centrifuged at 10,000 rpm, and supernatants were collected for CV quantification. The withdrawn samples were replaced with fresh solvent. All collected supernatants were measured using a UV–vis spectrophotometer at a λmax of 273 nm. The concentrations were determined using a calibration curve, and the cumulative release over 72 h was plotted against time intervals. The release profile was fitted using different kinetic models to extract the best one over 0–72 h. However, a Korsmeyer–Peppas model was fitted only at the initial stage of drug release up to 70%. The best kinetic model was determined based on the largest regression coefficient (R^2^) to identify the mechanism of release. The Korsmeyer–Peppas model was used to calculate the release parameters, including the release exponent (*n*) from the slope and the release rate (K) from the intercept.

### 2.7. In Vitro Study

#### 2.7.1. Collection and Preservation of *Cryptosporidium* Oocysts

Stool samples were collected from diarrheic calves and examined directly using concentration techniques to exclude the presence of any other parasitic infections other than *Cryptosporidium* spp. *Cryptosporidium* oocysts were identified via a modified Ziehl–Neelsen technique according to Henriksen & Pohlenz (1981) [28], preserved in 2.5% potassium dichromate (K_2_Cr_2_O_7_) solution, and stored at 4 °C; K_2_Cr_2_O_7_ effectively kills most viruses, vegetative bacteria, and fungi and significantly reduces offensive fecal odors [29].

#### 2.7.2. Mice Infection Protocol

BALB/c pathogen-free male Swiss Albino mice (20 ± 5 g) were orally infected with 3000 *Cryptosporidium* oocysts in 200 µL of phosphate-buffered saline solution (PBS) using a stomach tube. Seven days post-infection, fecal pellets were collected from mice, examined by direct wet mount and a modified Ziehl–Neelsen technique, as described by Henriksen and Pohlenz (1981) (Figure S1) [28]. Species identification was accomplished by estimating morphometric sizes of the *Cryptosporidium* oocysts using a micrometric slide that was examined using a light microscope and measured using ImageJ software, version 1.54 (National Institutes of Health (NIH), USA (Java 1.83.0)). After species identification, *Cryptosporidium* oocysts were collected, preserved in 2.5% K_2_Cr_2_O_7_, and stored at 4 °C until used. Just before use, the *Cryptosporidium* oocysts were purified through washing three times using sterile-filtered PBS (pH 7.4) and were repeatedly concentrated by sedimentation and centrifugation at 1500× *g* for 10 min to obtain clear *Cryptosporidium* oocysts sediment.

#### 2.7.3. In Vitro Oocysticidal Assessment

Collected oocysts were placed in 6-well plates at a density of 1 × 10^5^ oocyst/well. They were treated for 96 h at room temperature. The treatment groups were free CV, free MSN1, free MSN2, free MSN3, CV-MSN1, CV-MSN2, and CV-MSN3, compared to negative and positive controls, and for each preparation, several concentrations were included (0.125, 0.25, 0.5, 1, 2 mg/mL PBS). At the end of the incubation period, oocysts were examined for their count, deformation, and signs of damage. The viable *Cryptosporidium* oocysts per 1 mL were counted after staining with 0.1% eosin staining using a counting chamber for 96 h. The viable *Cryptosporidium* oocysts were unstained, while non-viable counterparts were stained red. The viability percentage (V%) was calculated according to the following equation [30]:

$$V\left(\%\right)=\frac{C-E}{C}\times 100$$

where C is the oocyst count/mL in the control group, and E is the oocyst count/mL in the treated group.

The median lethal dose (LC_50_), or the concentration at which half of the *Cryptosporidium* oocysts inside the well were non-viable, was determined by nonlinear regression analysis using a sigmoidal dose–response (four-parameter logistic) model [31].

The deformations of *Cryptosporidium* oocysts morphology were observed using optical and scanning electron microscopy (SEM). Photographs of *Cryptosporidium* oocysts were taken with a LEICA ICC50 HD photomicroscope camera (Leica Microsystems, Wetzlar, Germany) using LAS EZ imaging software (version 2.1.0). SEM micrographs were visualized using field-emission SEM (FEG, Thermo Scientific, Quattro S, NL Breda, The Netherlands). *Cryptosporidium* oocysts were examined after fixation in 2.5% buffered glutaraldehyde, dehydrated in ascending concentrations of ethanol, and dried in a critical-point dryer. Finally, samples were sputter coated with gold using a sputter coater (JFC-1100E, JEOL Ltd., Tokyo, Japan) and examined at 10 KV.

The oocyst damage percentage was further calculated based on the following equation:

$$Damage\left(\%\right)=\frac{Damaged\;Oocyst\;count}{Total\;number\;of\;oocyst\;count}\times 100$$

The morphometric changes in *Cryptosporidium* oocysts were determined after identifying the reduction in their size compared to their normal size using ImageJ software, version 1.54 (National Institutes of Health (NIH), USA) after scale bar calibration [32]. Oocyst deformations were recorded in 100 *Cryptosporidium* oocysts of each sample compared to negative controls.

### 2.8. In Vivo Study

#### 2.8.1. Animals

The conducted animal experiments complied with ARRIVE guidelines and were in accordance with the NIH (National Research Council) Guidelines for the Care and Use of Laboratory Animals. Thirty BALB/c mice albino male mice (22 ± 5 g, 4–6 weeks) were used in this study, obtained from the Experimental Animal Facility of Kafrelsheikh University. The mice had a 7-day acclimatization period. All mice were kept in cages with proper ventilation, a free supply of water, and standard pellet food at a maintained temperature of 25 °C with 12 h of light and 12 h of darkness. Our study was approved by the Institutional Animal Care and Use Committee (approval number: KFS-IACUC/328/2026, approval date: 15/10/2025). Pain and distress were monitored through daily clinical observations, including changes in body weight, posture, grooming behavior, mobility, food and water intake, and response to handling. Signs such as lethargy, hunching, piloerection, diarrhea, or abnormal respiration were considered indicators of distress.

#### 2.8.2. Treatment Protocol

Infected mice were divided into five groups, each consisting of six mice based on body weight, as shown in Table 1. All treatment solutions were orally administered daily (the dose was calculated based on a preliminary pilot experiment, starting from the seventh day post infection for five consecutive days). All mice were sacrificed on the 21st day post-infection. The total body weights of the mice were recorded weekly. Blood and tissue samples were collected at the end of the experiment. All histological, IHC, and biochemical analyses were performed by a technician who was blinded to the group allocations.

#### 2.8.3. Biochemical Investigations

At the end of the experimental period, blood was collected using a fine heparinized capillary tube that was inserted into the retro-orbital sinus vein of mice in EDTA-vacutainer tubes. Blood was obtained on the 21st day post-infection and the 14th day post-treatment. The collected blood samples were centrifuged at 3000 rpm for 15 min to isolate sera. Finally, the isolated sera were used to determine serum biochemical parameters; namely, glutamate pyruvate transaminase (GPT), glutamate oxaloacetate transaminase (GOT), creatinine (CREA), and urea using a biochemistry analyzer (Microlab 300 Meril Diagnostics Pvt. Ltd., Vapi, Gujarat, India).

##### Histopathological Examination

Ileum (last 2 cm), liver, and lung sections were collected from anesthetized mice (via inhalation of ether vapor in a closed chamber) and fixed in 10% normal saline formalin for 36 h; these sections were dehydrated in ascending ethanol concentrations, embedded in paraffin wax blocks, and cut into 4 µm thick sections before being placed onto glass slides using a microtome (Leica RM2125 RTS rotary microtome, Leica Microsystems, Wetzlar, Germany). Slides were stained with hematoxylin and eosin (H&E) using the Bancroft and Gamble staining protocol. Sections were examined under a light microscope [33].

### 2.9. In Silico Bioinformatics Study

#### 2.9.1. Network Pharmacology Analysis

##### Protein–Protein Interaction (PPI) Network

A PPI network was constructed using the STRING database (https://string-db.org/) and subsequently visualized and analyzed using Cytoscape software (version 3.10.4) [34]. The input proteins were selected based on their established roles in inflammation and oxidative stress, with particular relevance to ileal pathology, based on the Gene Expression Omnibus database (https://www.ncbi.nlm.nih.gov/geo/, accessed on 1 September 2025 [35]). Core inflammatory signaling molecules, including TLR4, NF-κB, iNOS, p38-MAPK, JAK1, and STAT3—together with key pro-inflammatory cytokines (TNF-α, IL-6, and IL-1β)—were manually curated from the literature and incorporated into the network. Additional proteins (BCL10, ICAM1, TICAM2, IL1RL1, PPARG, HSP90B1, HSPA4, AREG, WNT5A, RC3H1, and NFAT5) were extracted from the Gene Expression Omnibus (GEO) dataset GSE11223 and selected based on differential expression criteria (log_2_ fold change (FC) > 2 and *p* < 0.05). Network construction was performed using the STRING database (https://string-db.org/), restricted to *Homo sapiens*, and only high-confidence interactions (confidence score ≥ 0.700) were retained. Network topology parameters, including the average node degree, were used to rank interaction relevance.

##### Gene Ontology (GO) and Kyoto Encyclopedia of Genes and Genomes (KEGG) Pathway Enrichment Analyses

Hub genes obtained from the PPI network analysis were imported into ShinyGO 0.85.1 for functional enrichment analysis (https://bioinformatics.sdstate.edu/go/, accessed on 1 September 2025 [36]). GO analysis was conducted by investigating the biological processes (BP), cellular components (CC), and molecular functions (MF) of hub genes. KEGG analysis was performed through the identification of significantly enriched signaling-, metabolic-, or disease-related pathways associated with the hub genes. Enrichment significance was evaluated through over-representation analysis, and multiple testing correction was applied using the Benjamini–Hochberg false discovery rate (FDR) method, with an adjusted FDR value < 0.05. Visualization was carried out with ShinyGO, a web-based graphical enrichment tool (https://bioinformatics.sdstate.edu/go/, accessed on 1 September 2025). Analysis was adjusted to *Homo sapiens*, and the minimum pathway size was adjusted to 2.

#### 2.9.2. Computational Studies

##### Ligand Preparation

The PubChem database (https://pubchem.ncbi.nlm.nih.gov/, accessed on 1 September 2025) was used to obtain the canonical smiles of CV (CV, 2-methyl-5-propan-2-ylphenol, MF: C_10_H_14_O, MW: 105 g/mol, compound CID: 10364) [37]. Moreover, the ACD/ChemSketch program, version 2024.2.0, Advanced Chemistry Development, Inc. (ACD/Labs), Toronto, ON, Canada was utilized to draw, clean, and optimize the chemical structure of CV, which was saved as an MDL MOL file.

For MSN, BIOVIA Materials Studio 20 was used to create an N-capped MSN nanocluster (5 Å) from the quartz crystal unit cell of SiO_2_ (space group: 152 P3121, lattice type: 3D hexagonal, crystal system: trigonal, IT number: 152), available in the program’s library. The amorphous MSN sphere was created using the amorphous cell, maintaining SiO_2_ stoichiometry, derived from quartz as a compositional reference while eliminating long-range crystalline order according to the XRD pattern of the synthesized MSN. This was performed with a density of 2.2 g/cm^3^, a temperature of 300 K, a universal force field, and ultrafine quality [38]. Geometry optimization of ligands was carried out using the Forcite module with convergence tolerance conditions as follows: ultrafine quality, energy of 2.0 × 10^−5^ Kcal/mol and 500 max iterations. OpenBabel GUI v2.3.2 software was used to convert ligand MDL MOL to PDB-file format with energy minimization.

To create the CV-MSN complex, the adsorption locator module was used to adsorb the CV molecule onto the MSN nanocluster surface with ultrafine conditions of convergence tolerance and universal force field, as shown in Scheme S1. Among the 10 configurations created, the most stable adsorption complex was selected based on the lowest adsorption energy. The selected structure was further geometry-optimized using the COMPASS force field to ensure energetic and structural stability [39].

##### Receptor Preparation

For target proteins, the X-ray crystal structures of inducible nitric oxide synthetase (iNOS, PDB ID: 4NOS, 2.25 Å), Janus Kinas–1 (JAK-1, PDB ID:3EYG, 2.25 Å), signal transducer and activator transcription factor 3 (STAT-3, PDB ID: 3CWG, 3.05 Å), toll-like receptor-4 (TLR4, PDB ID: 3FXI, 3.10 Å), and p38 mitogen-activated protein kinase-P65 (p38-MAPK-P65, PDB ID: 3ZSG, 1.89 Å) were retrieved from the RCSB-PDB database as PDB file formats. After removing all inhibitors and water molecules from the crystal structures, the polar hydrogens were added, and the energy was minimized.

##### Molecular Docking

Molecular docking was performed using Molegro Virtual Docker (MVD), version 6.0 (MVD), as suitable software to study the interactions between CV-MSNs and the previously mentioned receptors. MVD was chosen for this study as it is characterized by its high accuracy in identifying ligands like MSNs in docking studies, unlike other software [40]. The receptor was imported into the MVD working space, in which water and cofactors were removed. Polar hydrogens were added, and the mismatched amino acids were repaired. After ligand import, the largest cavity was detected as the binding active site where docking was performed. The docking process was carried out with a grid resolution of 0.30 Å to calculate the MOLDOCK score after 10 runs using 4 CPU processors (Intel, Santa Clara, CA, USA). The docking outputs were saved in MOL2 file format. The docking poses were visualized using Molegro Molecular Viewer 2.5 [41].

##### Global Reactivity Descriptors (GRDs)

To gain molecular insight into the anti-inflammatory behavior of free CVs compared with CV-MSNs, density functional theory (DFT) calculations were performed to evaluate the global reactivity descriptors (GRDs) derived from frontier molecular orbital energies. GRDs were calculated using Gaussian 09 software using conceptual density functional theory (DFT) at the B3LYP/6-31G basis set. The calculated GRDs were as follows: the highest occupied molecular orbital energy (E_HOMO_), the lowest unoccupied molecular orbital energy (E_LUMO_), HOMO-LUMO gap (∆E = E_LUMO_ − E_HOMO_), the chemical potential (µ = [E_LUMO_ + E_HOMO_]/2), electronegativity (χ) = −µ, the chemical hardness (η = [E_LUMO_ − E_HOMO_]/2), the global electrophilicity (ω = µ^2^/2η), and the chemical softness (S = 1/2η). All energies calculated were expressed in eV [42].

### 2.10. Immunohistochemical (IHC) Examinations

Ileum sections embedded in paraffin wax were sectioned into 4 µm thick sections. They were deparaffinized in xylene and rehydrated into descending ethanol concentrations. Blocking was carried out using bovine serum albumin (BSA, 5%) in Tris buffer solution (TBS) for 2–3 h. Immunostaining was performed using iNOS, NF-ĸB-p65, JAK-1, STAT-3, TLR4, and p38-MAPK primary antibodies (1:100 dilution) overnight at 4 °C. After washing three times in PBS, slides were incubated with horseradish peroxidase (HRP)-loaded goat anti-rabbit secondary antibody at room temperature for 1 h. Then, slides were incubated in 0.05% diaminobenzidine (DAB) and 0.01% H_2_O_2_ for 3 min to enhance the peroxidase reaction color. Counterstaining of the slides was carried out using hematoxylin for 1 min. Finally, the slides were examined under a light microscope, and IHC scoring was quantified via ImageJ software, version 1.54 (National Institutes of Health (NIH), USA [43]).

### 2.11. Assessment of Oxidative Stress

Parts of the ileum were collected, washed with sterile PBS buffer (pH 7.4), and stored at −80 °C for further analysis of GSH, SOD, CAT, and MDA using the enzyme-linked immunosorbent assay (ELISA) technique, as described by the supplier’s protocol [44].

### 2.12. Assessment of Cytokines Levels

Pro-inflammatory cytokines (IL-1β, IL-6, and TNF-α) were estimated in the supernatant of ileum homogenate using ELISA kits following the manufacturer’s instructions (Thermo Fisher Scientific, Waltham, MA, USA).

### 2.13. qRT-PCR Analysis

Frozen ileum sections from each treatment group were used to quantify the gene expression of iNOS, NF-ĸB-P65, JAK-1, STAT-3, TLR4, p38-MAPK, and GAPDH. Total RNA was extracted from tissues using Direct-zol RNA Miniprep Plus (Cat# R2072, Zymo Research Corp., Irvine, CA, USA). RNA purification was assessed using a Beckman dual spectrophotometer (Beckman Coulter, Brea, CA, USA) at a λmax of 260 nm. Reverse transcription of the purified RNA was carried out with a SuperScript IV One-Step RT-PCR kit (cat #12594100, Thermo Fisher Scientific, Waltham, MA, USA). SYBER green master mix was used to amplify and quantify target gene expression. The prepared reaction mix samples were applied in real-time PCR (StepOne™ Real-Time PCR System, Applied Biosystems, Foster City, CA, USA). The relative gene expression of target genes was quantified versus the corresponding housekeeping gene (*GAPDH*). The relative quantification of genes was calculated and normalized to GAPDH gene expression by using the ΔΔCt (2^−ΔΔCt^) equation [45]. Primer sequences (cat #12594100, Thermo Fisher Scientific, Waltham, MA, USA) are listed in Table S1.

### 2.14. Statistical Analysis

Statistical analysis was carried out using Statistical Package for the Social Sciences (SPSS) 20.0 software. Before *p*-value calculations, the normality and homogeneity of measured data were evaluated using the Kruskal–Wallis and Levene’s tests, respectively. The statistical significance was calculated using one-way analysis of variance (ANOVA) with Tukey’s post hoc comparison test. Significance was indicated by a * when a *p*-value ≤ 0.05 and the sample size (*n*) was triplicate according to the resource equation method.

## 3. Results and Discussion

### 3.1. Characterization of Free MSNs

**Figure 1:**
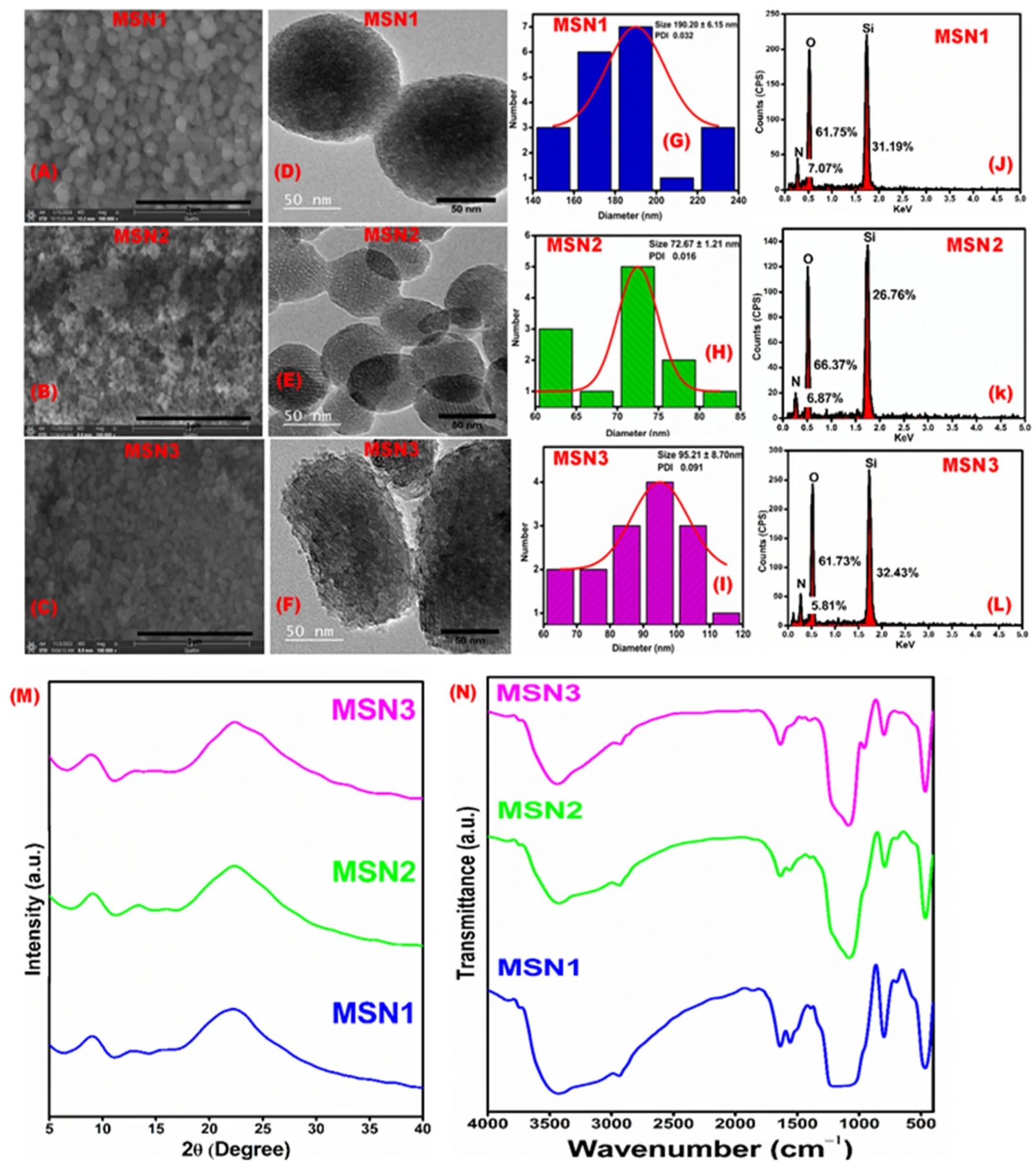
Characterization of MSNs: SEM (**A**–**C**), TEM micrographs (**D**–**F**), particle size distribution histograms (**G**–**I**), EDX spectra (**J**–**L**), XRD patterns (**M**), and FTIR spectra (**N**).

Controlled modulation of the synthesis conditions enabled the fabrication of three distinct amino-functionalized mesoporous silicate nanoparticles (MSNs) with a precisely tailored size and morphology. MSN1, synthesized via a conventional sol–gel process, exhibited large spherical particles, with an average diameter of 190.26 ± 6.15 nm and a narrow size distribution (PDI ≈ 0.032) (Figure 1A,D,G), which is consistent with previous reports [46]. In contrast, hydrothermal synthesis promoted rapid nucleation and condensation, yielding a smaller and highly homogeneous spherical MSN2 (72.67 ± 1.21 nm, PDI ≈ 0.016) (Figure 1B,E,H), which is in agreement with earlier studies [47]. Further modulation of precursor addition kinetics induced anisotropic growth, producing rod-shaped MSN3 (95.21 ± 8.70 nm, PDI ≈ 0.091) (Figure 1C,F,I), which is consistent with the anisotropic condensation mechanisms reported previously [48,49]. These findings highlight the extreme sensitivity of nanostructure formation to subtle synthetic variations, a fundamental hallmark of nanotechnology that necessitates systematic optimization and rigorous physicochemical characterization.

Amino functionalization resulted in a slight increase in particle size relative to the corresponding bare MSNs, which is in line with our previous observations [26] and literature reports describing surface grafting and mesopore modification [50,51,52,53]. SEM–EDX analysis confirmed the formation of silica frameworks (Si:O ≈ 1:2) and verified successful amine incorporation through the detection of nitrogen (~5.7%) (Figure 1J–L). XRD patterns revealed a broad amorphous halo centered at ~22°, along with a weak quartz reflection at 2θ ≈ 8–9°, confirming the predominantly amorphous nature of the synthesized MSNs (Figure 1M) [54,55]. FTIR spectra further validated surface amination, displaying characteristic N–H bending bands at 1560–1562 and 1640–1643 cm^−1^, alongside typical Si–O–Si vibrations (Figure 1N) [56].

Importantly, surface amination confers a positive surface charge through protonated amine groups, which is widely recognized to enhance colloidal stability, minimize nanoparticle aggregation, and promote cellular uptake via electrostatic interactions with negatively charged biological membranes [57,58,59,60]. Moreover, the obtained particle size range (≈70–190 nm) lies within the optimal window for biomedical applications, favoring prolonged circulation, enhanced cellular internalization, and efficient intracellular drug delivery [61]. Overall, these physicochemical attributes are expected to significantly improve the biological performance of CV, as nanoparticle size, morphology, and surface charge are key determinants of bioavailability, intracellular trafficking, and therapeutic efficacy. The ability to generate distinct MSN architectures through minor synthetic modifications further underscores the critical importance of rational nanoparticle design in optimizing nanocarrier-based therapeutic systems.

### 3.2. Characterization of CV-MSNs

**Figure 2:**
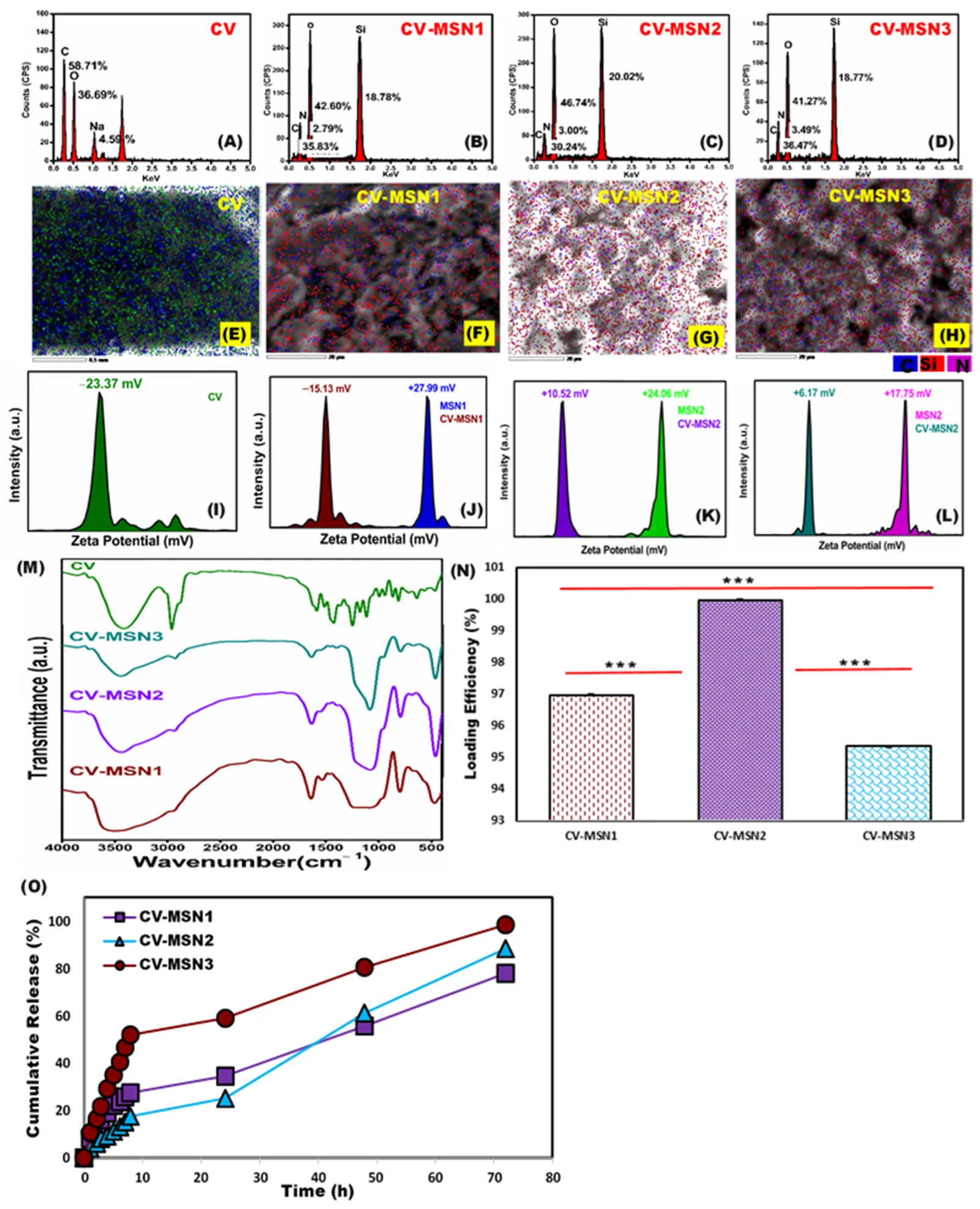
Characterization of free CV and CV-MSNs: SEM-EDX (**A**–**D**), SEM-MAPS (**E**–**H**), Zeta potentials (**G**–**I**), EDX spectra (**H**–**L**), FTIR spectra (**M**), loading effeciency (**N**); and (**O**) cumulative release profiles. Data are shown as means ± SD (*n* = 3). *** Significant at *p* < 0.001. Scale bars for all map images are 20 um execpt in CVmap (0.5 mm).

The successful incorporation of CV into NH_2_-functionalized MSNs was confirmed through complementary physicochemical characterization techniques (Figure 2). EDX analysis of free CV revealed predominant carbon and oxygen contents of 58.71% and 36.69%, respectively, with trace sodium (4.59%). Upon CV loading, a pronounced increase in carbon signal intensity was observed across all MSN formulations, accompanied by a homogeneous carbon distribution in the corresponding elemental maps (Figure 2E–H), providing direct evidence of CV deposition on the nanoparticle surfaces. These findings indicate efficient surface coverage and a uniform dispersion of CV within the MSN framework.

Zeta potential measurements further corroborated CV incorporation (Figure 2I–L). The synthesized MSN1, MSN2, and MSN3 exhibited positive surface charges of +27.99, +24.06, and +17.75 mV, respectively, due to the presence of protonated surface amino groups. Following CV loading, these values decreased markedly to −15.13, +10.52, and +6.17 mV, respectively, reflecting the partial neutralization and masking of surface amines by the negatively charged CV colloidal dispersion under the measurement and loading conditions, such as dispersion in distilled water, rather than being attributed to the intrinsic charge of individual carvacrol molecules (≈−23.37 mV) [62]. This charge reversal and attenuation confirm strong interfacial interactions between CV and the functionalized silica surface, which are critical for stabilizing the nano-formulations and enhancing drug–carrier affinity. However, CV is neutral under normal conditions of its oil form, owing to its pKa 10.2–10.5.

The differences in the magnitude of the zeta potential change among MSN1, MSN2, and MSN3 may be attributed to differences in particle size, morphology, pore architecture, surface area, pore accessibility, and the distribution of surface amino groups, all of which influence the extent and mode of CV incorporation. Consequently, although MSN1 and MSN2 exhibited similar initial zeta potential values, their distinct structural characteristics resulted in different surface charge changes after CV loading [63].

FTIR spectroscopy provided a molecular-level insight into the nature of these interactions (Figure 2M). The spectrum of free CV displayed characteristic absorption bands at 3417 and 1428 cm^−1^ (O–H stretching and bending), 2960 and 812 cm^−1^ (C–H stretching and bending), and 1586 and 1249 cm^−1^ (C=C and C–O–C stretching), respectively [57]. Upon incorporation into MSNs, a notable increase in the intensity of the 1640–1643 cm^−1^ band was observed, indicating interactions between the NH_2_ groups of MSNs and the C=C moieties of CV. Additionally, enhanced Si–O–Si bending vibrations in the 456–471 cm^−1^ region suggest strengthened host–guest interactions within the silica framework, likely mediated by hydrogen bonding and confinement effects. The attenuation of O–H band intensity further reflects partial surface coverage of silanol groups by hydrophobic CV molecules. Collectively, these results confirm the efficient incorporation of CV while preserving the mesoporous structure and surface chemistry of the MSNs.

### 3.3. CV Loading Efficiency

Quantitative evaluation of loading efficiency, as shown in Figure S2, revealed exceptionally high CV encapsulation (>90%) across all MSN formulations (Figure 2N), reflecting the strong affinity between the phenolic and hydrophobic functionalities of CV and the NH_2_-/silanol-rich MSN surfaces [58,59]. Among the formulations, MSN2 achieved the highest loading efficiency (99.98 ± 0.02%), followed by MSN1 (96.98 ± 0.01%) and MSN3 (≈95.35 ± 0.015%). These data were validated by analytical validation parameters (regression equation, correlation coefficient (R^2^), LOD, and LOQ) calculated in Table S2. These results demonstrate the effectiveness of the employed coupling protocol, which is notably simple, rapid, and readily scalable, making it particularly suitable for large-scale synthesis and translational research. In contrast to more sophisticated and technically demanding loading strategies, this facile approach is more amenable to upscaling and reproducibility, especially in early-stage R&D and industrial manufacturing contexts.

Importantly, this study represents the first reported formulation of CV-loaded MSNs, addressing critical limitations associated with CV, including poor aqueous solubility, volatility, and limited bioavailability. Encapsulation within mesoporous silica matrices not only markedly improves loading efficiency but also provides structural protection, enhances colloidal stability, and enables passive targeting through the enhanced permeability and retention (EPR) effect.

The increased loading performance of MSN2 might be attributed to its spherical morphology, which offers shorter internal diffusion pathways and enhanced pore accessibility [60]. Although some studies have reported higher drug loading capacities for rod-shaped MSNs due to their increased surface-area-to-volume ratios [64,65], the present findings indicate that pore accessibility and internal diffusion length play more dominant roles in governing CV loading behavior. To validate this hypothesis, future investigations employing the Brunauer–Emmett–Teller (BET) technique to quantitatively assess pore size distribution and molecular diffusion kinetics are warranted.

### 3.4. CV Release Profile and Kinetic Behavior

Figure 2O depicts the cumulative release profiles of CV from MSN1, MSN2, and MSN3 in a saline–ethanol medium (80:20, *v*/*v*) over 72 h at 37 °C, representing a near-neutral physiological environment. A mixture of normal saline and ethanol (80:20, *v*/*v*) was used because CV is poorly soluble in aqueous media, and this mixture increased the solubility of CV in saline for accurate CV quantification. Overall, rod-shaped MSNs (CV-MSN3) showed a faster release of CV than spherical counterparts (CV-MSN1 and CV-MSN2). This may be due to the extended interconnected channel system.

Release kinetics were investigated by fitting the obtained experimental data to zero-order, first-order, Higuchi, Hixson–Crowell, and Korsmeyer–Peppas kinetic models (Figures S3–S5). Based on the largest regression coefficients (R2), the cumulative CV release from MSNs was governed by the Higuchi model, reflecting release mainly by diffusion. Furthermore, a non-Fickian (anomalous) mechanism was confirmed by the release exponent (*n*) calculated from the Korsmeyer–Peppas model, ranging between 0.45 and 0.89. This indicated that CV release from the synthesized MSNs occurred by diffusion and desorption through the matrix (Table 2) [66], which is consistent with a previous study [67]. The sustained release observed was aligned with an earlier study reporting that the mesoporous system gives MSNs the ability to control release kinetics and enhance structural stability [68].

The release rates were evaluated by the calculated K from the Korsmeyer–Peppas model, which were 4.34, 6.60, and 9.95%·h^−0.5^ for CV-MSN1, CV-MSN2, and CV-MSN3, respectively. This indicates that CV release was faster in the case of MSN3 compared to MSN2 and MSN1. A preliminary study mentioned that release occurred by diffusion via the mesoporous channels due to the reduced matrix interaction and increased pore accessibility [69].

### 3.5. In Vitro Oocysticidal Results

#### 3.5.1. Evaluation of *C. parvum* Oocyst Count

In vitro evaluation of *C. parvum* oocysts revealed a concentration- and time-dependent reduction in viability following incubation with free CV and CV-loaded MSNs (Figure S6). Free CV induced moderate oocysticidal activity, reducing oocyst viability to 16.34 ± 1.70% at 2 mg/mL after 96 h, with LC50 values decreasing six-fold from 0.58 mg/mL at 48 h to 0.10 mg/mL at 96 h, whereas significantly lower activity was detected at 24 h. Coupling CV to MSNs markedly enhanced its efficacy in a morphology- and formulation-dependent manner. CV-MSN1 and CV-MSN3 reduced oocyst viability to 5.65 ± 3.35% and 16.8 ± 0.98%, respectively, after 96 h, with LC50 declining from 0.66 to 0.088 mg/mL (CV-MSN1) and from 0.90 to 0.09 mg/mL (CV-MSN3). Remarkably, CV-MSN2 exhibited the most potent and sustained activity, lowering viability to 4.62 ± 1.12% at 0.25 mg/mL after 96 h and reducing LC50 from 0.11 to 0.07 mg/mL, outperforming free CV and all other nanoformulations (Table S3).

#### 3.5.2. Evaluation of *C. parvum* Oocyst Damage

**Figure 3:**
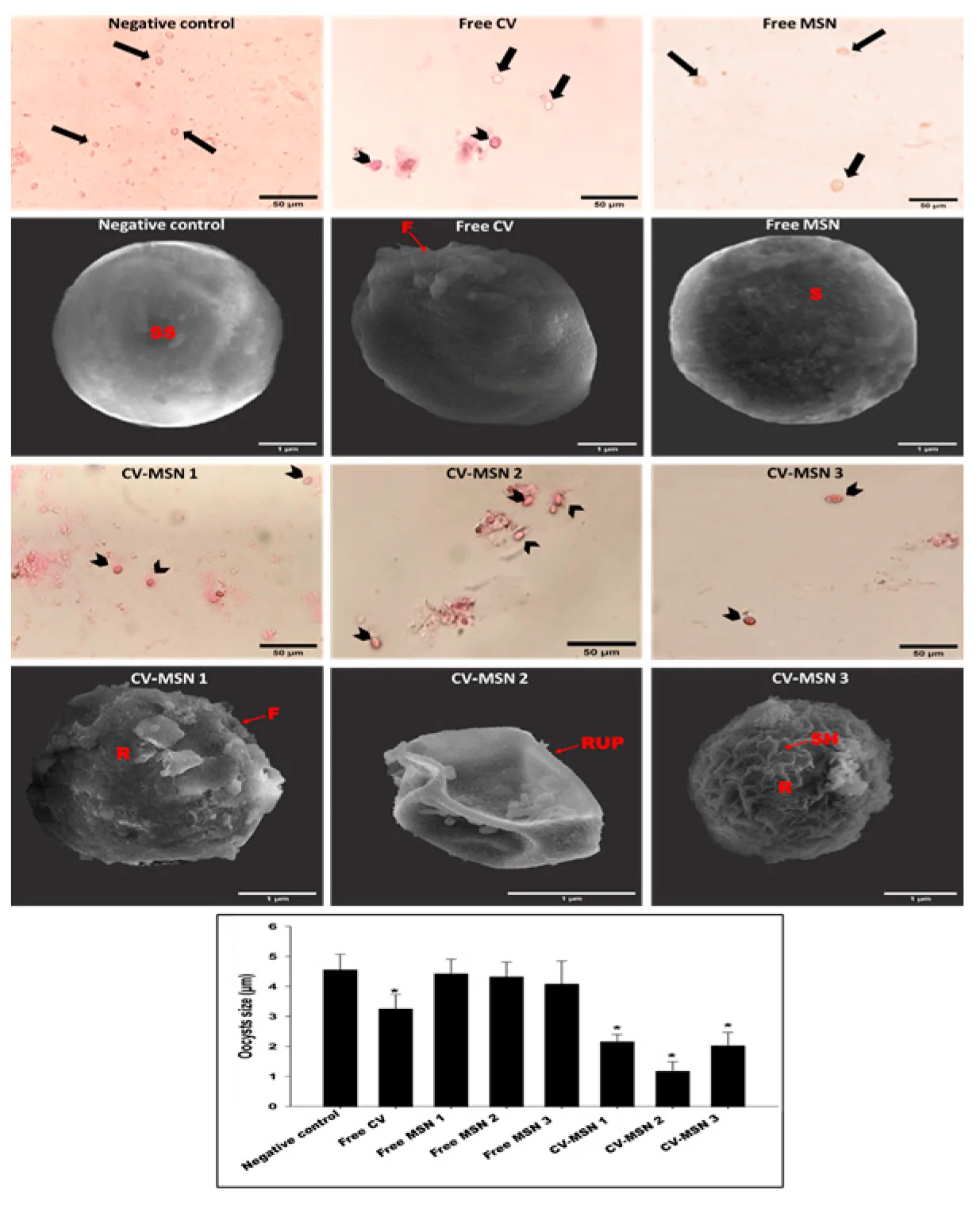
Photo- and SEM micrographs of stained *C. parvum* oocysts with 0.1% eosin stain after 96 h treatment with 2 mg/mL of the tested materials and morphometric changes in *Cryptosporidium* oocysts’ size graph. (viable *Cryptosporidium* oocysts; unstained (bold arrow), non-viable; stained red (arrowhead), (SS) smooth and spherical, (F) surface fissure, (S) spherical oocyst covered with NPs, (R) rough oocyst, (RUP) complete rupture of oocyst wall, and (SH) oocyst wall shrinkage. A plot showing the morphometric changes in *Cryptosporidium* oocysts sizes after treatment. Scale bar = 50 µm (photomicrographs) and 1 µm (SEM micrographs). Data are shown as means ± SD. * Significant at *p* < 0.05 against negative control.

Optical and SEM analyses confirmed these observations; normal *C. parvum* oocysts exhibited round to oval shapes with a smooth surface and an average size of 4.56 ± 0.51 µm (Figure 3 and Figure S7). Incubation with free MSNs caused no significant changes in oocyst morphology or size (4.09 ± 0.75 to 4.42 ± 0.47 µm; *p* > 0.05), confirming their inert nature, consistent with reports describing minimal interaction between silica nanoparticles and viable *Cryptosporidium* oocysts [70].

In contrast, free CV induced a notable reduction in oocyst size (3.26 ± 0.47 µm), accompanied by surface fissures, indicating partial structural damage. Earlier studies reported only moderate anti-cryptosporidial activity of oregano essential oil and its major constituent, CV, largely based on in vitro models. Initial work showed that CV-containing oregano oil reduced *C. parvum* infection in HCT-8 cells, although sporozoite invasion was not specifically evaluated [20]. A subsequent study demonstrated that neither crude oregano oil nor isolated CV inhibited sporozoite invasion in the same cell line [71]. Other studies suggested that CV exerts indirect anti-cryptosporidial effects through mechanisms such as induction of oxidative stress, inhibition of phospholipases, and modulation of Ca^2+^ signaling. Definitive molecular targets remained unclear [21]. More recent studies revealed that CV and oregano derivatives may inhibit calcium-dependent protein kinase-1 (CDPK1), thereby disrupting Ca^2+^-dependent signaling pathways essential for parasite invasion and viability [72]. Consistently, oregano oil and CV suppressed *C. baileyi* and *C. galli* oocyst infection in HCT-8 cells [73], while CV and thymol reduced *Cryptosporidium* infectivity and oocyst shedding by interfering with parasite growth, development, invasion, and metabolic activity [22,74]. Collectively, these findings indicate a moderate inhibitory efficacy of free CV, with reported inhibition rates of approximately 45% against *C. parvum* oocysts.

CV-loaded MSNs markedly enhanced oocysticidal effects in a formulation-dependent manner, with CV-MSN1-treated *Cryptosporidium* oocysts exhibiting multiple surface fissures and a significant size decrease to 2.17 ± 0.23 µm, and CV-MSN3-treated *Cryptosporidium* oocysts showing pronounced wall shrinkage and a reduced size to 2.03 ± 0.43 µm. Strikingly, CV-MSN2 caused the most severe structural alterations, including complete cell wall rupture and a four-fold reduction in oocyst size to 1.19 ± 0.29 µm (Figure 3). These observations indicate that nanoformulation of CV not only amplifies its anti-cryptosporidial activity but also enhances its direct physical disruption of oocyst integrity. Furthermore, the observed *C. parvum* oocyst may be due to the sustained release of CV from MSNs that maintains its therapeutic effect for an extended period. Taken together with the LC50 and viability data, CV-MSN2 consistently demonstrated increased potency, establishing it as the most promising formulation for further in vivo and mechanistic studies. This observation is consistent with previous studies demonstrating that spherical NPs exhibit higher cellular uptake than rod-shaped counterparts [75]. Moreover, it was reported that small-sized spherical nanoparticles are preferentially internalized by biological barriers in general, and *Cryptosporidium* oocysts in particular [76]. A third contributing factor identified in this study is the higher L.E.% and enhanced CV retention, as indicated by the lower release rate constant (K) of CV-MSN2 compared with the other CV-MSN formulations.

### 3.6. In Vivo Results

#### 3.6.1. Effect on Total Body Weight

**Figure 4:**
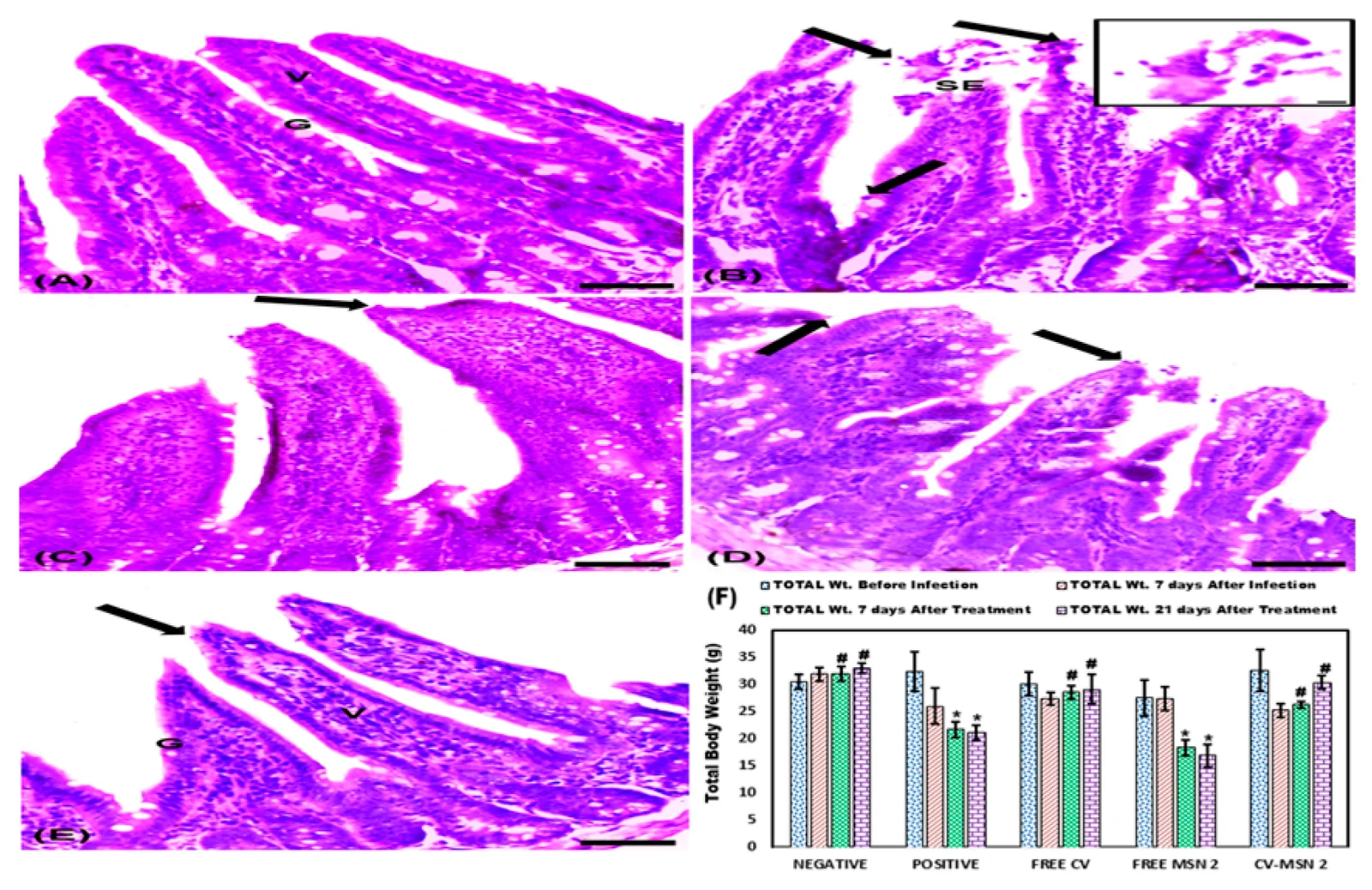
Microscopic photographs of H&E-stained ileal tissue of *C. parvum*-infected mice: (**A**) Negative control showing normal intestinal architecture had long, slender, and well-organized villi (V) with an intact epithelial lining composed of enterocytes and interspersed goblet cells (G). (**B**) Positive control demonstrates blunted and atrophied villi, with sloughing epithelium (SE), disintegration, and loss of typical villus morphology. A huge number of *C. parvum* parasitic stages (arrows) appeared, adhering to the apical surface of enterocytes. Dense infiltration of inflammatory cells within the lamina propria was found. Inset = 10 µm. (**C**) The free CV-treated group exhibited restoration of normal villus architecture, reduced inflammatory infiltration, and improved epithelial integrity but was still blunted with a moderate number of *C. parvum* parasitic stages (arrow). (**D**) The free MSNs2-treated group showed marked villus atrophy with blunting, epithelial disruption, and dense inflammatory cell infiltration, with a remarkable appearance of *C. parvum* parasitic stages (arrows). (**E**) The CV-MSN2-treated group appeared to provide the greatest histological improvement, with long normal villi (V) and goblet cells (G) most closely resembling the negative control. Damaged and very few *C. parvum* parasitic stages (arrows) appeared. Scale bar = 100 µm. (**F**) Total body weight changes after treatment. Data are shown as means ± SD. * Significant at *p* < 0.05 against negative control. ^#^ Significant at *p* < 0.05 against positive control.

*C. parvum* infection exerted severe systemic toxicity in mice, which is consistent with its well-established intestinal tropism and extra-intestinal dissemination. Infected (positive control) mice exhibited a pronounced body weight loss, declining from 30.02 ± 2.15 g pre-infection to 21.12 ± 1.43 g at 21 days post-infection, reflecting chronic diarrhea and malabsorption. A comparable or greater weight loss was observed in the free MSN2-treated mice, indicating a lack of therapeutic benefit. In contrast, free carvacrol (CV) significantly improved body weight to 28.57 ± 1.22 g after 7 days and 29.10 ± 2.71 g after 21 days of treatment, while CV-MSN2 treatment resulted in near-complete restoration of body weight (30.33 ± 1.19 g), closely matching the negative controls, highlighting its increased efficacy (Figure 4).

#### 3.6.2. Effect on Histopathological Changes

Histopathological evaluation corroborated these findings. Infection induced severe ileal damage, characterized by villous atrophy, epithelial sloughing, inflammatory cell infiltration, and abundant Cryptosporidium oocysts, confirming the intestine as the primary target organ [77]. Similar lesions persisted in free MSN2-treated mice. Free CV partially ameliorated intestinal injury, as evidenced by moderate villous regeneration and reduced inflammation. Notably, CV-MSN2 treatment achieved the most pronounced histological recovery, restoring elongated, well-organized villi with abundant goblet cells, closely resembling normal architecture (Figure 4). These results align with the observed recovery in body weight and underscore the enhanced therapeutic performance of the nano-formulated CV.

Extra-intestinal organs exhibited parallel trends. *C. parvum* infection caused marked hepatic degeneration, including central vein hemorrhage, sinusoidal dilation, nuclear pyknosis, and inflammatory infiltration, accompanied by significant two-fold elevations in serum SGPT (43.63 ± 0.55 U/L) and SGOT (288 ± 1 U/L) levels. Compared to the positive control, free MSN2 treatment failed to mitigate these alterations, with nonsignificant changes in levels of SGPT (30.23 ± 3.87 U/L) and SGOT (205.67 ± 7.37 U/L), whereas free CV treatment moderately improved hepatic architecture and enzyme profiles (SGPT: 16.33 ± 3.37 U/L; SGOT: 44.1 ± 6.49 U/L). However, the decrease in liver enzymes following free CV treatment was not within the normal physiological range, as indicated by statistical significance compared to the negative controls, indicating recovery from hepatic injury. Strikingly, CV-MSN2 treatment restored normal hepatic organization with intact sinusoids and near-baseline transaminase levels (SGPT: 20.67 ± 1.15 U/L; SGOT: 104.3 ± 0.52 U/L), indicating its therapeutic benefits (Figure S8). Similarly, pulmonary inflammation manifested as bronchial infiltration and alveolar collapse in infected and free MSN2 groups, while CV-MSN2 treatment preserved well-expanded alveoli with minimal inflammation (Figure S9).

#### 3.6.3. Effect on Kidney Functions

Evaluating renal clearance and nephrotoxicity through kidney function analysis is a critical step in validating the safety of newly developed nano-formulations and therapeutic agents. Renal function analysis further supported the safety and efficacy of CV-MSN2. Infection markedly elevated serum urea and creatinine levels (urea, 384.23 ± 9.92 mg/dL; creatinine, 2.36 ±1.46 mg/dL), compared to the negative controls (urea, 68.42 ± 2.81 mg/dL; creatinine, 0.64 ± 0.17 mg/dL). Similarly, mice treated with free MSN2 exhibited a significant increase (*p* < 0.05) in the levels of blood urea (359.56 ± 6.89 mg/dL) and creatinine (2.16 ± 0.58 mg/dL) compared to the negative controls, indicating a lack of renal protection. In contrast, mice treated with free CV revealed normal levels of blood urea (57.34 ± 1.32 mg/dL) and creatinine (0.56 ± 0.04 mg/dL), compared to the negative controls. Furthermore, CV-MSN2-treated mice maintained normal kidney function biomarker levels (urea, 62.19 ± 2.57 mg/dL; creatinine, 1.08 ± 0.88 mg/dL) that were not significantly different from the negative control group (Figure S16).

Collectively, these findings demonstrate that CV-MSN2 confers robust therapeutic benefits against *C. parvum* infection, surpassing free CV and bare nanoparticles. This enhanced in vivo performance is consistent with previous reports on CV-rich botanicals, which reduced oocyst shedding, improved histopathology, and normalized biochemical parameters [16,22,73]. Notably, the present study provides the first evidence that CV-MSN2 confers a marked therapeutic benefit in vivo, as demonstrated by substantial restoration of histopathological architecture and normalization of biochemical markers compared with the positive control. Although silica nanoparticles alone have been reported to partially reduce oocyst shedding and moderately improve histopathology, heterogeneity in their physicochemical properties limits direct comparison [78].

Differences between in vitro and in vivo nanoparticle performance have been previously attributed to biological interactions unique to the in vivo environment. Protein corona formation has been shown to alter nanoparticle surface properties, cellular uptake, and biological activity, potentially explaining discrepancies between bare nanoparticles tested in vitro and their in vivo counterparts [79]. In in silica nanoparticles, protein corona adsorption was reported to enhance nanoparticle exocytosis, which may compromise drug delivery efficiency [80]. Conversely, nano-suspensions have been shown to adhere to intestinal mucosa and prolong gastrointestinal residence time during *Cryptosporidium* treatment, a critical in vivo advantage that is absent from in vitro systems [81]. These factors collectively support the enhanced therapeutic performance of CV-MSN2 observed in vivo.

### 3.7. Network Pharmacology Results

#### 3.7.1. PPI Network Analysis

Network pharmacology analysis was used to investigate the possible pathway crosstalk between the proteins involved in the inflammation and oxidative stress responses. Figure S10 showed that the predicted PPI model was composed of 20 nodes and 37 edges, with an average node degree of 3.7 and a high clustering coefficient (0.654). The observed number of interactions was significantly higher than the expected number (six interactions; PPI enrichment *p* < 1.0 × 10^−16^). Some proteins, including NPTN, RC3H, UBXN, and WNTSA, showed no interaction in the constructed model; however, their role in GIT inflammation was reported in previous studies [82]. Table S4 confirmed the importance of TLR4, STAT-3, NF-ĸB, NOS2, and JAK-1, with the help of pro-inflammatory cytokines, particularly IL-6 and IL-1β, in the constructed model. The predicted interactions of proteins involved in the constructed model are shown in Table S5. Thus, PPI analysis revealed a highly interconnected network with higher observed interactions, indicating that the selected proteins are functionally associated and participate in coordinated inflammatory and oxidative stress signaling pathways rather than representing a random gene set [83].

#### 3.7.2. GO Enrichment Analysis

GO enrichment analysis was conducted to investigate the biological significance of the identified hub genes in the PPI network in terms of biological processes (BP), cellular components (CC), and molecular functions (MF). Furthermore, the central role of these identified genes was in orchestrating host inflammatory and oxidative stress responses during *Cryptosporidium* infection.

As shown in Figure S11, the main BP terms were immune and inflammatory responses, including responses to type I interferon, cytokine production, and nitric oxide biosynthetic production. This indicates elevated oxidative stress and IL-8 production, which is known to occur in intestinal damage due to cryptosporidiosis [4,84,85]. The main CC terms included perinuclear region of the cytoplasm, receptor complex, the phagocytic cup, and peroxisomal matrix, which are closely related to immune signaling, pathogen recognition, and redox reactions involved in inflammatory pathways [86,87]. Also, the main MF terms showed that the hub genes were strongly associated with inflammatory and oxidative reactions, including nitric oxide synthase activity, tetrahydrobiopterin binding, arachidonic acid binding, lipopolysaccharide binding, and prostaglandin receptor activity [88].

#### 3.7.3. KEGG Pathway Enrichment Analysis

KEGG pathway enrichment analysis was used to investigate the biological pathways involved in the hub genes or proteins. Figure S11D visualized the top 20 signaling pathways that explained inflammatory processes, including HIF-1 signaling, lipid and atherosclerosis, PPAR signaling, and arginine metabolism. Also, several infectious pathways were enriched, including toxoplasmosis, amoebiasis, leishmaniasis, and tuberculosis. These results indicated involvement of hub genes mainly in oxidative stress related to protozoan infections [89].

Overall, these findings indicate that *Cryptosporidium* infection activates a coordinated inflammatory–oxidative stress network involving cytokine signaling, nitric oxide production, lipid mediator regulation, and HIF-1-dependent pathways, which may represent critical therapeutic targets for alleviating intestinal injury and inflammation.

### 3.8. Computational Results

#### 3.8.1. Molecular Docking of Free CV and CV-MSNs

Molecular docking was performed to assess the impact of mesoporous silica nanoparticle loading on the binding affinity of CV toward key inflammatory targets, including iNOS, JAK-1, STAT-3, TLR4, and p38-MAPK. Docking calculations were conducted using the MolDock scoring function, which accounts for hydrogen bonding, electrostatic and steric interactions, and internal ligand strain energy [90]. Based on established criteria, MolDock scores between −40 and −80 indicate moderate binding affinity, whereas values more negative than −120 correspond to very strong ligand–protein interactions [91]. Binding stability was further evaluated using hydrogen bond energy (H-bond) and internal energy of the docked pose (E_int_), reflecting intermolecular stabilization and ligand conformational strain, respectively [92]. An approximately 5 Å silica nanocluster was used as a simplified model of MSNs rather than the entire experimental MSNs (≈70 nm) to investigate the molecular interactions of CV and the silica surface with target proteins at the atomic level as a qualitative mechanistic insight.

Table 3 shows that CV-MSN exhibited stronger binding affinities with target proteins compared with free CV, indicated by the more negative MolDock scores.

#### TLR4/p38-MAPK Proteins

Docking in TLR4 (PDB ID: 3FXI) confirmed a strong binding affinity with CV-MSNs, with MolDock scores exceeding −180 compared with moderate affinity for free CV (−71). MSN loading enabled broader residue engagement and improved conformational stabilization, as reflected by more favorable E_int_ values. Furthermore, CV-MSNs displayed stable interactions with Lys561, Leu511, and Pro514, indicating increased accommodation within the TLR4 binding pocket, as shown in Figure S12A–D.

Docking in p38-MAPK (PDB ID: 3ZSG) with CV-MSN (−242) demonstrated a 3-fold increase in the negative MolDock score compared to free CV (−70), with stable interactions with Arg57, Lys152, Arg70, Asn155, Lys53, and Gly170 inside p38-MAPK. Meanwhile, free CV showed hydrogen bonds and steric interactions with Ala51, Leu104, Lys53, Val52, Val38, Thr106, and Leu75, as visualized in Figure S12E–H.

#### iNOS Protein

Docking in iNOS (PDB ID: 4NOS) revealed a 4-fold increase (−242) in the MolDock score of CV-MSN compared with free CV (−60) due to strong hydrogen bonds and steric interactions with amino acid residues, including Gln263, Tyr347, Arg266, Asn354, Arg388, Tyr491, Thr121, Pro350, Arg381, Tyr373, Met120, Trp463, Val352, Gly371, Ala351, Ala353, Ala282, Asp382, and Glu377, accompanied by a strongly negative E_int_ value (−102 kcal/mol). This reflected enhanced internal stabilization of the nano-assembly rather than ligand distortion. In contrast, limited steric interactions were formed in the case of free CV with Tyr491, as presented in Figure S12I–L.

#### JAK-1/STAT-3 Proteins

Docking in JAK-1 (PDB ID: 3EYG) demonstrated a 2-fold increase in the negative MolDock score from −57 in the case of free CV to −131 in CV-MSNs by the formation of hydrogen bonds and favorable steric interactions within Glu883, Ser963, Lys911, Leu881, Gly882, Glu966, and Arg1007. Meanwhile, free CV interacted with Val889 through steric interactions and formed a hydrogen bond with Glu883, as described in Figure S12M–P.

Similar trends were observed in the docking results of STAT-3 (PDB ID: 3CWG), which depicted a 2-fold increase in the MolDock score in CV-MSN (−109) compared to free CV (−60), with broader residue engagement and improved conformational stabilization, as reflected by more favorable E_int_ values. Figure S12Q–T displayed stable multiple interactions between CV-MSN and Lys574, Asp334, Lys340, Asn466, Lys642, Tyr575, Ile467, Met470, His332, Glu616, Cys468, and Asp570, while free CV formed fewer interactions with Lys561, Leu511, and Pro514.

Overall, docking confirmed that nano-formulation resulted in stronger accommodation of CV inside the active sites of target proteins with internal stabilization rather than ligand distortion compared with free CV, particularly in iNOS and p38-MAPK. This aligns with previous studies demonstrating the anti-inflammatory effect of CV through modulation of proteins such as Glutathione Peroxidase (GPX), NADPH oxidase homolog 1 (Nox1), Cyclooxygenase-2 (COX-2), TNF-α, and iNOS [25,93,94]. Additionally, molecular docking revealed that encapsulation of a pregabalin derivative in engineered MSNs enhanced its binding affinity compared to pregabalin alone [95].

#### 3.8.2. DFT Results for Global Reactivity Descriptors (GRDs)

The anti-inflammatory performance of CV was elucidated after loading onto MSNs from the electronic point of view by using DFT calculations. This provided insight into their electronic reactivity, stability, electrophilicity, and nucleophilicity.

Table S6 shows that there was a slight increase in the HOMO energy of CV after loading onto MSNs from −7.30 eV to −7.01 eV, indicating an electron-donating rather than electron-accepting tendency. Meanwhile, the LUMO energy of CV-MSNs exhibited a marginal shift to −6.86 eV compared to free CV (−4.60 eV), as shown in Figure S13. This leads to an 18-fold decrease in the HOMO–LUMO gap (ΔE) after loading onto MSNs, which ensures enhanced chemical reactivity. Additionally, the global hardness (µ) revealed a 19-fold decrease for CV-MSNs, while the global softness (S) increased by 18-fold. The chemical potential (µ) was shifted from −5.95 to −6.94 eV, reflecting a strong electronic tendency toward biological targets. The nucleophilicity (N) demonstrated a moderate enhancement from 1.94 eV to 2.23 eV after loading onto MSNs [96]. These findings confirmed the surface polarization of CV after loading onto MSNs that increases chemical reactivity while stabilizing conformation and redox homeostasis [97,98,99].

This was consistent with previous studies that calculated GRDs to evaluate the inflammatory activity of some phytochemicals [100,101]. However, the antioxidant activity of CV was investigated using DFT, ignoring the anti-inflammatory activity [102,103]. Although preliminary studies were interested in exploring the anti-inflammatory and antioxidant activities of NPs, like γ-alumina nanoparticles, silicon nanotubes, silver- or gold-loaded silica nanostructures, and carbon nanotubes, MSNs were not included in similar studies [104,105].

Thus, this study explains the role of MSNs in tuning the anti-inflammatory and antioxidant performance of CV by in silico methods. The mechanistic understanding of this is illustrated through the observed steric and electronic modifications.

Building on these computational insights, the anti-inflammatory activity of CV-MSN2 was further validated in vivo through analysis of key signaling proteins, including TLR4, p38-MAPK, NF-κB P65, iNOS, JAK1, and STAT3, alongside pro-inflammatory cytokines (TNF-α, IL-1β, IL-6) by using immunohistochemistry, qRT-PCR, and antioxidant mediators (MDA, SOD, GSH, and CAT) by ELISA.

### 3.9. Anti-Inflammatory Mechanisms Against C. parvum

Inflammation induced by *C. parvum* infection arises from the coordinated activation of interconnected innate immune pathways rather than isolated signaling events. However, the molecular crosstalk governing these pathways remains incompletely defined. The present study provides an integrated analysis of the TLR4/p38-MAPK, NF-κB-p65/TNF-α/IL-1β, iNOS, and IL-6/JAK-1/STAT-3 axes, delineating how loading of CV onto MSNs reshapes inflammatory signaling at both the transcriptional and translational levels.

#### 3.9.1. Effect on TLR4/p38-MAPK Pathway

TLR4 is considered the protein that initiates the inflammatory events accompanied by *C. parvum* infection by activating NF-κB-p65 and p38-MAPK. Positive control mice exhibited a four- and six-fold upregulation in TLR4 and p38-MAPK protein expression, respectively, compared to the negative controls. These results were aligned with studies reporting that *Cryptosporidium* infection significantly activated TLR4, NF-ĸB, and p38-MAPK [8,99].

**Figure 5:**
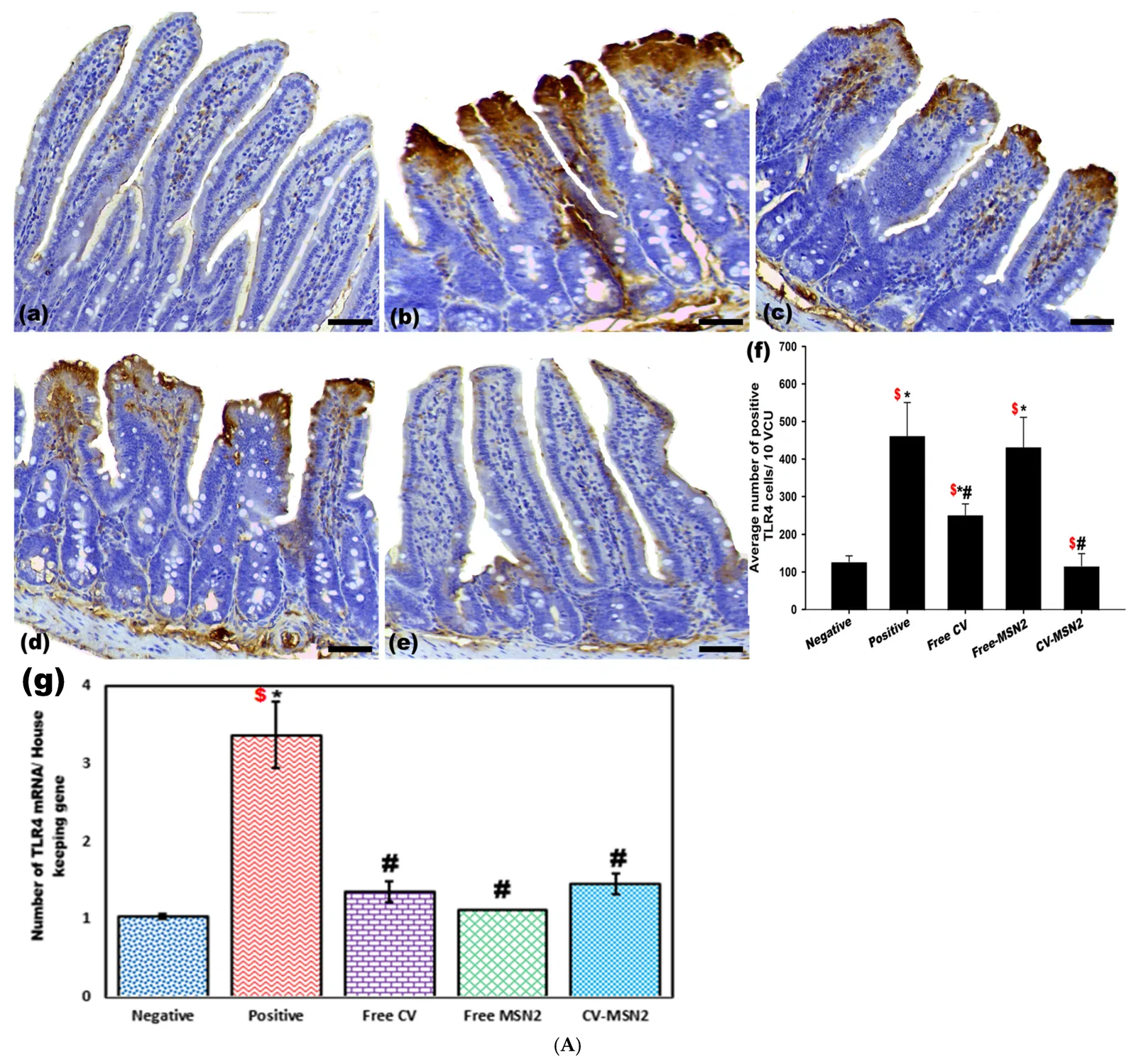

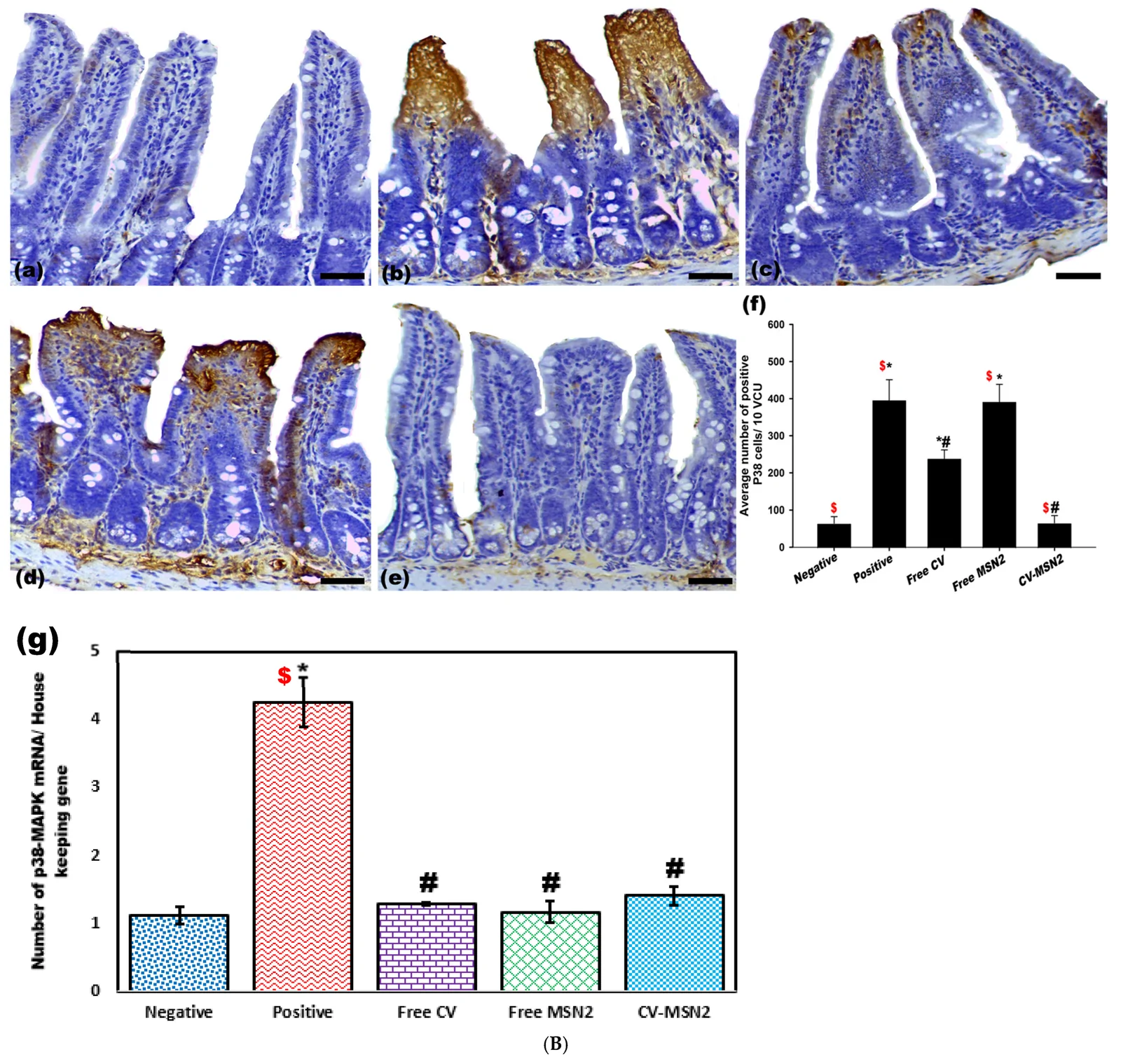
(**A**) IHC photographs of cytoplasmic and cytomembrane TLR4 protein expression in ileal tissue of *C. parvum*-infected mice: (**a**) Negative control. (**b**) Positive control. (**c**) Free CV. (**d**) Free MSN2. (**e**) CV-MSN2-treated groups. Scale bar = 100 µm. (**f**) IHC scoring of TLR4-positive stained cells. (**g**) TLR4 gene expression determined by qRT-PCR. Data are shown as means ± SD. * Significant at *p* < 0.05 against negative control, ^#^ significant at *p* < 0.05 against positive control, and ^$^ significant at *p* < 0.05 against free CV. (**B**) IHC photographs of cytoplasmic and nuclear p38-MAPK protein expression in ileal tissue of *C. parvum*-infected mice: (**a**) Negative control. (**b**) Positive control. (**c**) Free CV. (**d**) Free MSN2. (**e**) CV-MSN2-treated groups. Scale bar = 100 µm. (**f**) IHC scoring of p38-MAPK-positive stained cells. (**g**) p38-MAPK gene expression determined by qRT-PCR. Data are shown as means ± SD. * Significant at *p* < 0.05 against negative control, ^#^ significant at *p*< 0.05 against positive control, and ^$^ significant at *p* < 0.05 against free CV.

Compared to positive controls, Free MSN2-treated mice showed negligible effects at the levels of TLR4 and p38-MAPK protein, with a decrease in the mRNA transcription of TLR4 and p38-MAPK to 1.11 ± 0.004 and 1.17 ± 0.15, respectively. In contrast, free CV treatment resulted in a two-fold decrease in the protein expression of TLR4 and p38-MAPK, with a significant downregulation in mRNA expression of TLR4 to 1.34 ± 0.13 and p38-MAPK to 1.29 ± 0.02, compared to positive control mice. The best inhibitory activity in TLR4 and p38-MAPK expression was observed after CV-MSN2, with four- and six-fold downregulation in protein expression, with a significant decrease in their mRNA transcription (TLR4: 1.45 ± 0.13; p38-MAPK: 1.41 ± 0.14) (Figure 5A,B). These results are consistent with preliminary studies that exhibited the inhibitory activity of CV in TLR4, inhibiting p38-MAPK activation, and limits downstream cytokine production [106].

#### 3.9.2. Effect of NF-ĸB-P65/TNF-α/IL-1β Pathway

**Figure 6:**
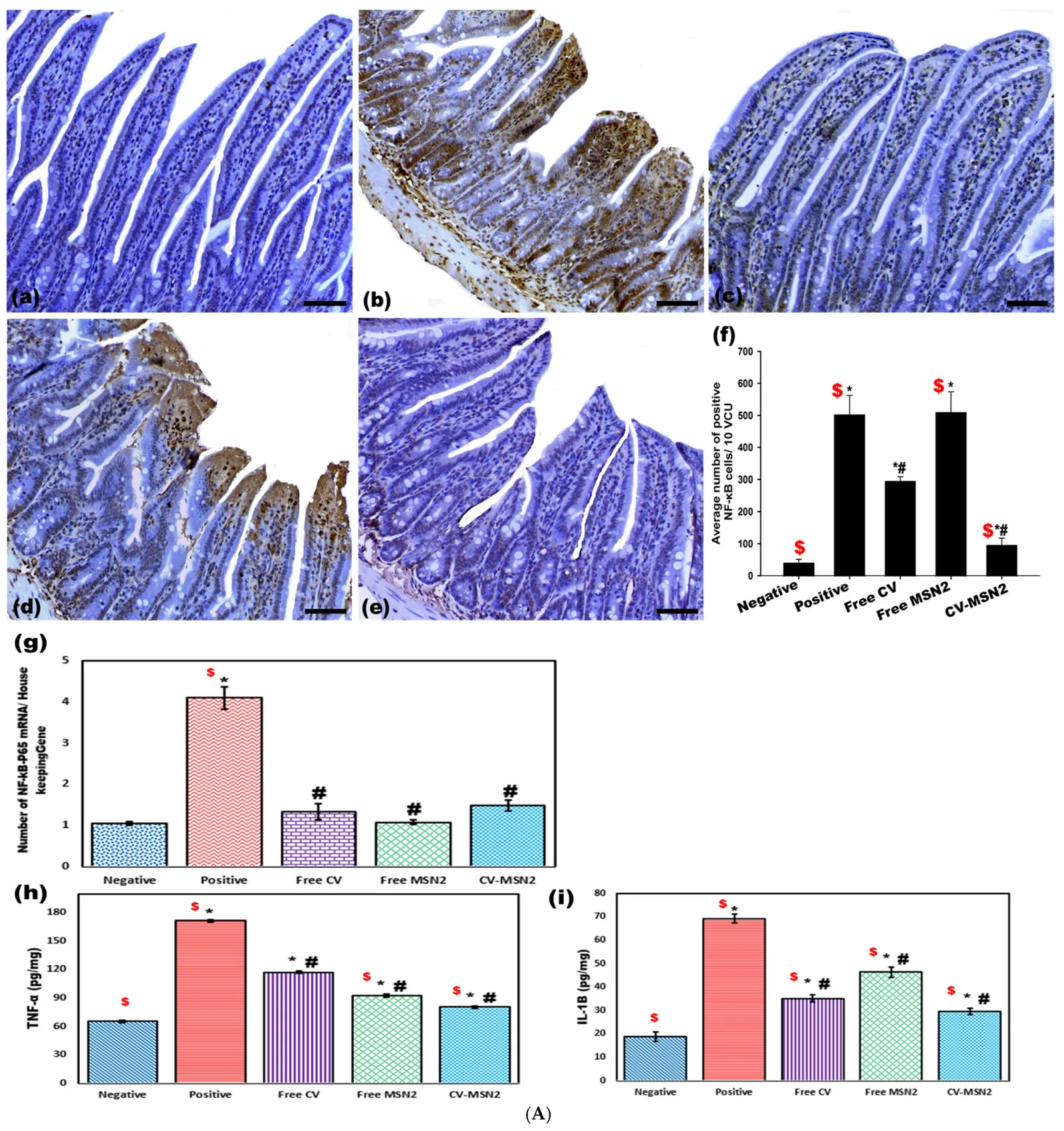

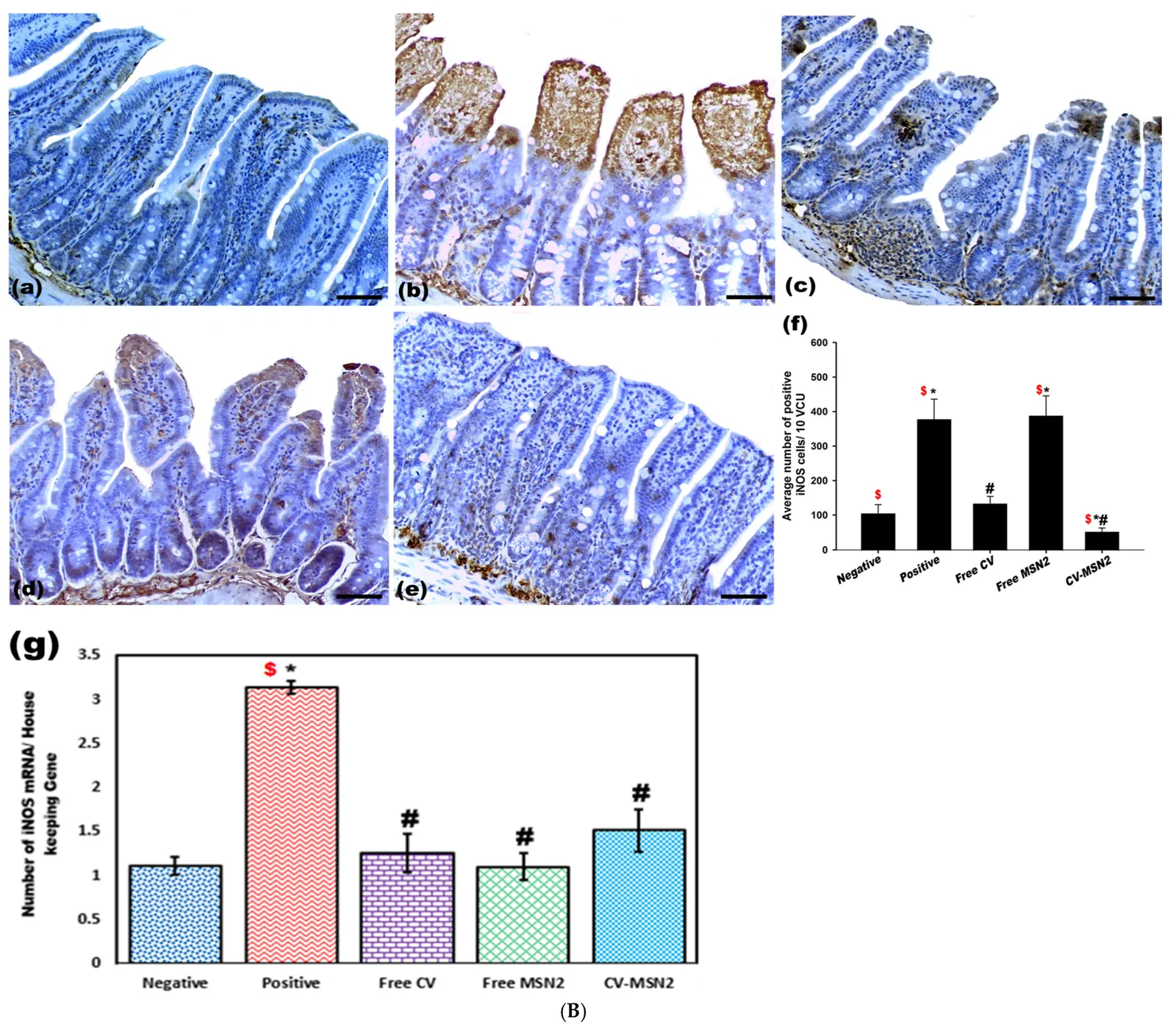
(**A**) IHC photographs of cytoplasmic and nuclear NF-κB-P65 protein expression in ileal tissue of *C. parvum*-infected mice: (**a**) Negative control. (**b**) Positive control. (**c**) Free CV. (**d**) Free MSN2. (**e**) CV-MSN2-treated groups. Scale bar = 100 µm. (**f**) IHC scoring of NF-κB-positive stained cells. (**g**) NF-κB-P65 gene expression by qRT-PCR. ELISA results of TNF-α (**h**) and IL-1β (**i**). Data are shown as means ± SD. * Significant at *p* < 0.05 against negative control, ^#^ significant at *p* < 0.05 against positive control, and ^$^ significant at *p* < 0.05 against free CV. (**B**) IHC photographs of cytoplasmic and nuclear iNOS protein expression in ileal tissue of *C. parvum*-infected mice: (**a**) Negative control showed minimal iNOS immunoreactivity. (**b**) Positive control exhibited strong brown iNOS immunostaining, primarily in the villus epithelium. (**c**) The free CV-treated group showed moderate iNOS expression, localized mainly to the basal and crypt regions. (**d**) The treated-MSN2 group displayed distinct iNOS staining in the villus tips and crypts. (**e**) The CV-MSN2-treated group revealed markedly diminished iNOS immunoreactivity, approaching the negative control level. Scale bar = 100 µm. (**f**) IHC scoring of iNOS-positive stained cells. (**g**) iNOS gene expression by qRT-PCR. Data are shown as means ± SD. * Significant at *p* < 0.05 against negative control, ^#^ significant at *p* < 0.05 against positive control, and ^$^ significant at *p* < 0.05 against free CV.

NF-κB-p65 acts as the main inflammatory protein that activates TNF-α, IL-1β, and iNOS. As shown in Figure 6A, positive controls showed seven-fold upregulation in NF-κB-p65 protein expression and its gene expression to 4.10 ± 0.28 mRNA. Also, free MSN2 treatment caused no significant change at the protein level while reducing its gene expression to 1.71 ± 0.05 mRNA, compared to positive controls. While free CV-treated mice demonstrated a two-fold decrease at the protein level and a three-fold decrease at the gene level. Interestingly, CV-MSN2 treatment exerted a five-fold decrease in NF-κB-p65 protein expression with concurrent transcriptional downregulation (1.49 ± 0.13 mRNA).

Positive controls exhibited a significant elevation in TNF-α to 171.6 ± 1.35 pg/mg and IL-1β to 69.2 ± 1.8 pg/mg, with respect to negative controls, similar to previous studies [107,108]. Free MSN2 treatment significantly decreased the levels of blood TNF-α (92.5 ± 1.4 pg/mg) and IL-1β (46.3 ± 2.2 pg/mg), which was consistent with preliminary studies that confirmed the cytokine attenuation related to MSN-mediated ROS scavenging [109,110]. A stronger inhibitory effect was observed after free CV treatment (TNF-α: 117.2 ± 1.22 pg/mg; IL-1β: 35.1 ± 1.5 pg/mg); however, CV-MSN2 treatment resulted in the most effective reduction in TNF-α (80.3 ± 1.44 pg/mg) and IL-1β (29.6 ± 1.4 pg/mg) (Figure 6A). These results highlighted the strongest NF-Κb-P65 inhibitory effect, with a significant recovery in serum levels of TNF-α and IL-1β following *C. parvum* infection.

#### 3.9.3. Effect on iNOS Expression Pathway

As a downstream effector of NF-κB signaling, iNOS contributes to excessive nitric oxide production and oxidative tissue injury [88]. *C. parvum* infection induced a significant four-fold increase in iNOS protein and gene expression. Free MSN2 caused no significant change in iNOS protein levels despite transcriptional suppression (1.10 ± 0.16 mRNA), whereas free CV elicited a three-fold reduction in protein expression with decreased gene expression (1.26 ± 0.22 mRNA). Remarkably, CV-MSN2 achieved a seven-fold reduction in iNOS protein expression alongside significant transcriptional downregulation (1.52 ± 0.24 mRNA) (Figure 6B), consistent with previous reports describing CV-mediated inhibition of iNOS activity and reactive nitrogen species generation [111].

#### 3.9.4. Effect on IL-6/JAK-1/STAT-3 Pathway

This pathway is activated to induce chronic inflammation through sustained NF-κB-p65 release [112,113]. Positive control mice showed eight-fold upregulation in protein expression of JAK-1 and STAT-3, accompanied by increased gene expression (JAK-1: 4.08 ± 0.45; STAT-3: 3.71 ± 0.24 mRNA). Meanwhile, free MSN2-treated mice showed minimal changes at the protein level, unlike their gene transcription (JAK-1: 1.13 ± 0.07; STAT-3: 1.11 ± 0.10 mRNA). Free CV treatment exerted a decrease in JAK-1/STAT-3 signaling by approximately two-fold, whereas CV-MSN2 produced the strongest suppression, decreasing their protein levels by six- and four-fold, respectively, with corresponding gene downregulation of JAK-1 to 1.49 ± 0.10 mRNA and STAT-3 to 1.37 ± 0.16 mRNA.

Similarly, mice infected with *C. parvum* showed a three-fold increase in IL-6 levels in serum, compared to negative controls. CV-MSN2 achieved the maximal inhibitory effect, an approximately four-fold decrease, while free CV revealed a two-fold decrease compared with positive controls. However, the weakest inhibitory effect in decreasing IL-6 compared with positive controls was observed after free MSN2 treatment, a one-fold decrease, as shown in Figures S14 and S15.

In general, CV-MSN2 succeeded in significantly increasing the anti-inflammatory activity of CV at the protein level compared to free CV. Consequently, this led to a decrease in pro-inflammatory cytokines, counteracting *C. parvum* infection. Free MSN2 showed a minimal influence at the protein level, with comparable changes at the gene level of the inflammatory proteins, reducing the release of pro-inflammatory cytokines. This behavior can be explained by earlier studies showing that silica NPs were effectively influenced at the gene level without affecting the protein levels of target inflammatory proteins [114,115]. Previous studies explained this antagonist behavior due to nanoparticle-induced endoplasmic reticulum stress and activation of the unfolded protein response that restricts de novo protein synthesis [116]. Also, silica NPs were reported to have a decreased translational effect on cytokine or COX-2 protein production [117,118].

Consequently, these findings confirmed the inert nature of bare MSNs at the protein level of expression, whereas CV-MSN2 exhibited the best anti-inflammatory efficacy at the protein and gene levels of expression.

#### 3.9.5. Antioxidant Mechanisms Against *C. parvum*

As shown in Figure S16, *C. parvum* infection induced a significant increase in MDA levels (25.5 ± 0.15 nmol/g), with a significant decrease in antioxidant enzymes, including SOD (57.8 ± 1.8 U/g), GSH (0.47 ± 0.06 nmol/g), and CAT (2.0 ± 0.4 U/g), compared to negative control mice (MDA: 6.7 ± 0.1 nmol/g; SOD: 143.1 ± 1.3 U/g; GSH: 2.46 ± 0.08 nmol/g; CAT: 7.3 ± 0.22 U/g). This was aligned with earlier studies that reported the stimulatory effect of *C. parvum* infection on ROS production, leading to decreased antioxidants and increased lipid peroxidation [119,120].

Treatment with free MSN2 resulted in a one-fold decrease in MDA level (17.8 ± 0.32 nmol/g) and minimal increase in SOD (77.4 ± 1.6 U/g), GSH (1.12 ± 0.02 nmol/g), and CAT (3.8 ± 0.26 U/g) compared with positive controls. This can be due to the adsorption of ROS onto silanol in MSNs [121,122]; however, this was reported at higher doses [123].

Compared to positive controls, free CV-treated mice exhibited a two-fold decrease in MDA levels (13.1 ± 0.2 nmol/g) with a two-fold increase in serum levels of SOD (102.2 ± 1.7 U/g), GSH (1.63 ± 0.03 nmol/g), and CAT (5.1 ± 0.35 U/g) activity, which is in agreement with previous reports [124]. Interestingly, CV-MSN2 exhibited the most pronounced protective effect, achieving the greatest suppression of lipid peroxidation (MDA: 11.2 ± 0.25 nmol/g) and better recovery in antioxidant enzymes close to negative controls (SOD: 119.6 ± 1.4 U/g; GSH: 2.17 ± 0.04 nmol/g; CAT: 5.9 ± 0.15 U/g). Generally, the results confirmed that *C. parvum* infection disturbed the redox homeostasis, causing severe oxidative stress. Meanwhile, CV loaded onto MSN2 showed the best antioxidant homeostasis due to the ROS-buffering capacity of MSNs, which increase the antioxidant activity of CV. Compared to free CV, CV-MSN2 significantly enhanced antioxidant activity following *C. parvum* infection, indicating a greater restoration of antioxidant status toward normal levels.

Collectively, the therapeutic performance of the CV-MSN formulation was reported from the overall findings, including sustained drug release, enhanced anti-parasitic activity, improved histopathological outcomes, and favorable biochemical safety.

## 4. Conclusions

This study employed cryptosporidiosis as a novel inflammatory model to conduct an integrated experimental and in silico evaluation of CV loaded onto optimized MSNs. Primarily, MSN2 was chosen as the best NPs based on the oocysticidal in vitro test. Preliminary in silico analysis was carried out to identify key inflammatory protein targets for subsequent in vivo validation based on network pharmacology, molecular docking, and DFT calculations. In vivo techniques were conducted using IHC, ELISA, and qRT-PCR to elucidate the mechanistic crosstalk between inflammatory and antioxidant pathways. Mechanistically, CV-MSN2 exhibited the best anti-inflammatory effect against TLR4/p38-MAPK/NF-κB/iNOS and IL-6/JAK-1/STAT-3 signaling pathways and significantly suppressed the synthesis of pro-inflammatory cytokines (TNF-α, IL-6, and IL-1β). In parallel, the antioxidant performance of CV-MSN2 exceeded that of free CV through the upregulation of antioxidant defenses (SOD, GSH, and CAT) and a decrease in lipid peroxidation, thereby limiting damage due to oxidative stress.

However, the synthesized CV-MSNs lacked active targeting, which constitutes a limitation of this study. The release kinetics experiment was performed only to evaluate the CV release from the MSN formulation, and no comparison was conducted with free CV, which could provide additional information about the influence of nanoparticle formulations. It is recommended to include a comparison between a nitazoxanide-treated control group and the developed nano-formulation so as to establish its relative efficacy to current standard therapy and clinical relevance. Future investigations should investigate active targeting moieties to achieve greater tissue selectivity and optimize pharmacological outcomes. Furthermore, comprehensive storage stability studies under different environmental conditions based on intensity-weighted particle size distributions should be conducted in future investigations to establish the shelf life and pharmaceutical colloidal stability and homogeneity of the developed formulation.

Overall, the present study demonstrated the therapeutic potential of a CV-MSN nano-formulation following *C. parvum* infection. This opens the door for using NPs for nanomedicine integration in clinically relevant conditions and translation into human applications.

## Figures and Tables

**Table 1:** Treatment protocol of the in vivo study.

Group No.	Group Name	Treatment
A	Negative control	Non-infected, untreated
B	Positive control	Infected, untreated
C	Free CV control	Infected, treated with CV in olive oil (80 mg/Kg)
D	Free MSN2 control	Infected, treated with MSN2 in normal saline (440 mg/Kg)
E	CV-MSN2	Infected, treated with CV-MSN2 in normal saline (440 mg/Kg)

**Table 2:** Kinetic release parameters (K and n) and regression coefficients (R^2^) of CV release from MSNs fitted to different release models.

	R^2^					K (%/h^0.5^)	n
	Zero Order	First Order	Higuchi	Hixson	Korsmeyer Peppas		
CV-MSN1	0.9305	0.9564	**0.9717**	0.9596	0.6744	4.34	0.7513
CV-MSN2	0.9196	0.955	**0.9648**	0.9554	0.8645	6.60	0.4365
CV-MSN3	0.8068	0.9028	**0.9418**	0.9405	0.6553	9.95	0.7404

**Table 3:** Docking parameters from the interaction of free CV and CV-MSNs with TLR4, p38-MAPK, iNOS, JAK-1 and STAT-3.

Target Protein	Free CV			CV-MSN		
	MOLDOCK Score	H Bond	E_int_	MOLDOCK Score	H Bond	E_int_
TLR4	−71.71	−2.86	9.30	−183.02	−1.88	−114.29
p38-MAPK	−70.38	−2.93	7.15	−242.53	−9.90	−109.91
iNOS	−60.95	0	7.48	−232.42	−5.24	−102.20
JAK-1	−57.46	−1.66	7.39	−131.73	−3.91	−106.27
STAT-3	−60.09	−2.50	7.15	−109.05	−5.06	−100.82

## Data Availability

Data will be available upon request from the corresponding author.

## Supplementary Materials

The following supporting information can be downloaded at: https://www.mdpi.com/article/10.3390/pharmaceutics18091065/s1.

## Glossary

APTES	(3-Aminopropyl)triethoxysilane
CV	Carvacrol
*C. parvum*	*Cryptosporidium parvum*
DMF	N,N-Dimethylformamide
MSN	Mesoporous silicate nanoparticles
MTT	(3-(4,5-dimethylthiazol-2-yl)-2,5-diphenyltetrazolium bromide)
TEOS	Tetraethyl orthosilicate

## References

[1] Santos J.V.I., Silva W.I., Neto B.F.L., Feitosa T.F., Vilela V.L.R. (2025). Epidemiology of Human Cryptosporidiosis in Brazil: A Systematic Review Highlighting *Cryptosporidium parvum*. Trop. Med. Infect. Dis..

[2] Helmy Y.A., Hafez H.M.J.M. (2022). Cryptosporidiosis: From prevention to treatment, a narrative review. Microorganisms.

[3] Ashigbie P.G., Shepherd S., Steiner K.L., Amadi B., Aziz N., Manjunatha U.H., Spector J.M., Diagana T.T., Kelly P. (2021). Use-case scenarios for an anti-Cryptosporidium therapeutic. PLoS Neglected Trop. Dis..

[4] Saraav I., Huang W., Jung J., Xu R., Fu Y., McDonald K., Schriefer L.A., Rodgers R., Baldridge M.T., Newberry R.D. (2025). Early life infection with *Cryptosporidium parvum* induces inflammatory responses to dietary antigens. Gut Microbes.

[5] Rodrigues E., Pallett M.A., Straker L.C., Mkandawire T.T., Sala K., Collinson L., Sateriale A. (2025). Cryptosporidium modifies intestinal microvilli through an exported virulence factor. Cell Host Microbe.

[6] Pardy R.D., Wallbank B.A., Striepen B., Hunter C.A. (2024). Immunity to Cryptosporidium: Insights into principles of enteric responses to infection. Nat. Rev. Immunol..

[7] Yin J., Huang J., Zhou P., Li L., Zheng Q., Fu H. (2025). The role of TLR4/NF-kB signaling axis in pneumonia: From molecular mechanisms to regulation by phytochemicals. Naunyn-Schmiedeberg’s Arch. Pharmacol..

[8] Lu Y., Zhang X., Guan Z., Ji R., Peng F., Zhao C., Gao W., Gao F. (2025). Molecular pathogenesis of Cryptosporidium and advancements in therapeutic interventions. Parasite.

[9] Fathy E.A.E.-a., Abdel-Gaber S.A.-W., Ibrahim M.F.G., Thabet K., Waz S. (2024). Downregulation of IL-1β/p38 mitogen activated protein kinase pathway by diacerein protects against kidney ischemia/reperfusion injury in rats. Cytokine.

[10] Ontawong A., Duangjai A., Vaddhanaphuti C.S., Amornlerdpison D., Pengnet S., Kamkaew N. (2023). Chlorogenic acid rich in coffee pulp extract suppresses inflammatory status by inhibiting the p38, MAPK, and NF-κB pathways. Heliyon.

[11] Zhang B. (2025). Investigating the Anti-Inflammatory Mechanism of the Adiponectin Receptor Agonist, ALY688, in Iron Overload-Induced Macrophage Activation. https://hdl.handle.net/10315/43297.

[12] Swain J., Mohanty C., Bajoria A.A., Patnaik S., Gahlawat A.W., Nikhil K., Mohapatra S.R. (2025). Deciphering the metabolic landscape of colorectal cancer through the lens of AhR-mediated intestinal inflammation. Discov. Oncol..

[13] Hassanein E.H., Sayed A.M., El-Ghafar O.A.A., Omar Z.M., Rashwan E.K., Mohammedsaleh Z.M., Kyung S.Y., Park J.H., Kim H.S., Ali F.E.M. (2023). Apocynin abrogates methotrexate-induced nephrotoxicity: Role of TLR4/NF-κB-p65/p38-MAPK, IL-6/STAT-3, PPAR-γ, and SIRT1/FOXO3 signaling pathways. Arch. Pharmacal Res..

[14] Mączka W., Twardawska M., Grabarczyk M., Wińska K. (2023). Carvacrol—A natural phenolic compound with antimicrobial properties. Antibiotics.

[15] Masri S., Mohd N., Abu Kasim N.H., Razali M. (2025). Mechanistic Insight into the Antioxidant and Antimicrobial Activities of Palm Oil-Derived Biomaterials: Implications for Dental and Therapeutic Applications. Int. J. Mol. Sci..

[16] Taha N.M., Zalat R.S., Khaled E., Elmansory B.M. (2023). Evaluation of the therapeutic efficacy of some essential oils in experimentally immunosuppressed mice infected with *Cryptosporidium parvum*. J. Parasit. Dis..

[17] Zhang Y., Long Y., Yu S., Li D., Yang M., Guan Y., Zhang D., Wan J., Liu S., Shi A. (2021). Natural volatile oils derived from herbal medicines: A promising therapy way for treating depressive disorder. Pharmacol. Res..

[18] Zhang D., Gan R.-Y., Ge Y.-Y., Yang Q.-Q., Ge J., Li H.-B., Corke H. (2018). Research progress on the antibacterial mechanisms of carvacrol: A mini review. Bioact. Compd. Health Dis..

[19] Nurzyńska-Wierdak R. (2023). Phenolic compounds from new natural sources—Plant genotype and ontogenetic variation. Molecules.

[20] Gaur S., Kuhlenschmidt T.B., Kuhlenschmidt M.S., Andrade J.E. (2018). Effect of oregano essential oil and carvacrol on *Cryptosporidium parvum* infectivity in HCT-8 cells. Parasitol. Int..

[21] Gaur S., Kuhlenschmidt T.B., Kuhlenschmidt M.S., Andrade J.E. (2016). Oregano essential oil and carvacrol reduce *Cryptosporidium parvum* infectivity of HCT-8 cells. FASEB J..

[22] Kara E., Yasa Duru S., Gökpinar S., Duru Ö., Sevin S., Şenel Y., Kaya U. (2023). Investigation of the prophylactic and therapeutic effectiveness of oral thyme extract in rats experimentally infected with *Cryptosporidium parvum*. Veter. Res. Commun..

[23] Tusar M.T.T., Munna M.M.R., Ahmed M.H., Rahman M.M., Fatema K., Islam K.M., Ali S. (2025). Integrative Network Pharmacology, Molecular Docking, and Dynamics Simulation Guided Discovery of Anethole, Carvacrol, Carnosol, Nicotine, and Paeonol as Potential Therapeutics for Parkinson’s Disease. Cell Biochem. Biophys..

[24] Cao G., Liu J., Liu H., Chen X., Yu N., Li X., Xu F. (2023). Integration of network pharmacology and molecular docking to analyse the mechanism of action of oregano essential oil in the treatment of bovine mastitis. Vet. Sci..

[25] Badr A.M., El-Orabi N.F., Mahran Y.F., Bayoumy N.M., Hagar H., Elmongy E.I., Atawia R.T. (2023). In vivo and In silico evidence of the protective properties of carvacrol against experimentally-induced gastric ulcer: Implication of antioxidant, anti-inflammatory, and antiapoptotic mechanisms. Chem. Interact..

[26] Rashad M.M., El-Kemary M., Gaber S.A.A. (2025). Rod-shaped and Spherical Mesoporous Silica Nanoparticles for Doxorubicin Delivery for an Advanced Anticancer Activity. J. Drug Deliv. Sci. Technol..

[27] Beyaz H., Kavaz D., Rizaner N. (2025). Chitosan nanoparticle encapsulation of thymus capitatus essential oil: In vitro release, antioxidant, antibacterial activity and cytotoxicity in MDA-MB-231 cells. Pharm. Dev. Technol..

[28] Henriksen S.A., Pohlenz J.F.L. (1981). Staining of cryptosporidia by a modified Ziehl-Neelsen technique. Acta Vet. Scand..

[29] Arrowood M.J. (2002). In vitro cultivation of Cryptosporidium species. Clin. Microbiol. Rev..

[30] Elez R.M.M.A., Attia A.S.A., Tolba H.M.N., Anter R.G.A., Elsohaby I. (2023). Molecular identification and antiprotozoal activity of silver nanoparticles on viability of *Cryptosporidium parvum* isolated from pigeons, pigeon fanciers and water. Sci. Rep..

[31] Haridevamuthu B., Murugan R., Seenivasan B., Meenatchi R., Pachaiappan R., Almutairi B.O., Arokiyaraj S., Kathiravan M.K., Arockiaraj J. (2024). Synthetic azo-dye, Tartrazine induces neurodevelopmental toxicity via mitochondria-mediated apoptosis in zebrafish embryos. J. Hazard. Mater..

[32] Ismaeil H., Mira N.M., El-Ashram S., Thagfan F.A., Mubaraki M.A., Dkhil M.A., Meryk A., Kasem S.M. (2025). Activity of Coriandrum sativum methanolic leaf extracts against Eimeria papillata: A combined in vitro and in silico approach. Open Chem..

[33] Tunçel S., Gokçimen A., Kalkan N.S. (2025). Staining Tissues With Dyes Obtained From Safran, Curcumin and Henna Extract Instead of Hematoxylin and Eosin Used in Routine Tissue Staining. J. Surg. Care.

[34] Bathla D., Vindal V. (2026). Protein interaction network analysis: STRING and CYTOSCAPE. Harnessing Genomic Tools for Crop Improvement.

[35] Wu J., Zhang Y., Dong X., Wang K., Sun B. (2026). Experimental validation of cytokine-cytokine receptor interaction pathway related gene signatures and molecular subtypes in rheumatoid arthritis. Chin. J. Tissue Eng. Res..

[36] Najar M.A., Balaya Rex D.A., Modi P.K., Suchitha G., Amrutha S., Keshava Prasad T., Dagamajalu S.J. (2026). Phosphoproteomic profiling reveals signaling pathways modulated by *Annona muricata* leaf extract in oral adenosquamous carcinoma cells. bioRxiv.

[37] Khmil N.V. (2026). Effect of ribosomal protein mutation on macrolide binding affinity in Staphylococcus aureus: A molecular docking study. Low Temp. Phys..

[38] DeBoyace K., Bookwala M., Zhou D., Buckner I.S., Wildfong P.L. (2024). Understanding the Influence of API Conformations on Amorphous Dispersion Formation Potential Predictions using the R3m Molecular Descriptor. Mol. Pharm..

[39] Issa A.A., Kamel M.D., El-Sayed D.S. (2024). Depicted simulation model for removal of second-generation antipsychotic drugs adsorbed on Zn-MOF: Adsorption locator assessment. J. Mol. Model..

[40] Shoukat W., Hussain M., Ali A., Shafiq N., Chughtai A.H., Shakoor B., Moveed A., Shoukat M.N., Milošević M., Mohany M. (2025). Design, synthesis, characterization and biological screening of novel thiosemicarbazones and their derivatives with potent antibacterial and antidiabetic activities. J. Mol. Struct..

[41] Hafezi A., Khamar Z. (2025). Molecular Docking of Silymarin as a Ligand for Aromatase Enzyme and HIV-1 Reverse Transcriptase Using Molegro Virtual Docker. J. Microbiota.

[42] Farjallah M. (2025). DFT study on geometries, electronic structures and electronic absorption of Naphthalene. Eur. Phys. J. B.

[43] Shui X., Wang Y., Luo Y., Liang X., Chen T., Xia Y., Wei Q., Liao L. (2025). Protocol for the purification and culture of primary retinal ganglion cells and development of common pathological models. Histochem. J..

[44] Cecerska-Heryć E., Engwert W., Michałów J., Marciniak J., Birger R., Serwin N., Heryć R., Polikowska A., Goszka M., Wojciuk B. (2025). Oxidative stress markers and inflammation in type 1 and 2 diabetes are affected by BMI, treatment type, and complications. Sci. Rep..

[45] Hashemipetroudi S.H., Rezaei A., Kuhlmann M. (2025). Validation of Reference Genes for Accurate RT-qPCR Normalization in Aeluropus littoralis Under Drought, Cold, and ABA Treatments. Agronomy.

[46] Han Y., Zhang L., Yang W. (2024). Synthesis of mesoporous silica using the Sol–Gel approach: Adjusting architecture and composition for novel applications. Nanomaterials.

[47] Purwaningsih H., Pratiwi V.M., Purwana S.A.B., Nurdiansyah H., Rahmawati Y., Susanti D. (2018). Fabrication of Mesoporous Silica Nanoparticles by Sol Gel Method Followed Various Hydrothermal Temperature.

[48] Chirra S., Gujjula S.R., Siliveri S., Goskula S., Andugula C.M., Ponnala V.K., Narayanan V. (2025). An Investigation of the Characterization and Drug Delivery Potential of Ibuprofen Using Modified High Surface Area Porous Materials. Silicon.

[49] Kragten D.D., Fedeyko J.M., Sawant K.R., Rimer J.D., Vlachos D.G., Lobo R.F., Tsapatsis M. (2003). Structure of the silica phase extracted from silica/(TPA) OH solutions containing nanoparticles. J. Phys. Chem. B.

[50] Talavera-Pech W.A., Esparza-Ruiz A., Quintana-Owen P., Vilchis-Nestor A.R., Carrera-Figueiras C., Ávila-Ortega A. (2016). Effects of different amounts of APTES on physicochemical and structural properties of amino-functionalized MCM-41-MSNs. J. Sol-Gel Sci. Technol..

[51] Taghavi S., Shahnani M., Rafati H. (2023). Preparation and characterization of tamoxifen loaded silica and NH2 functionalized mesoporous silica nanoparticles as delivery systems against MCF-7 breast cancer cells. Iran. J. Basic Med. Sci..

[52] Rashidi L., Ganji F., Dezfoulian F. (2022). Amine-functionalized and non-functionalized mesoporous silica nanoparticles as the delivery system for tertiary butylhydroquinone (TBHQ). J. Porous Mater..

[53] He Y., Luo L., Liang S., Long M., Xu H. (2017). Amino-functionalized mesoporous silica nanoparticles as efficient carriers for anticancer drug delivery. J. Biomater. Appl..

[54] Sen K., Mondal N.K. (2020). Facile fabrication of amino-functionalized silicon flakes for removal of organophosphorus herbicide: In silico optimization. Water Conserv. Sci. Eng..

[55] Semeshko O., Hredzak S., Matik M., Briančin J., Dutkova E., Melnyk I. (2025). Innovative utilization of quartz: Creating nanosilica adsorbents for dye removal and norfloxacin sensing applications. J. Mater. Chem..

[56] Ahmed M.S., Hussein N.N., Sulaiman G.M., Khan R.A., Mohammed H.A. (2025). Piperacillin-loaded amine functionalized mesoporous silica nanoparticles: A new frontier in combating multidrug-resistant pathogenic bacteria through value-added piperacillin antibiotic. J. Drug Deliv. Sci. Technol..

[57] Di Matteo A., Stanzione M., Orlo E., Russo C., Lavorgna M., Buonocore G.G., Isidori M. (2026). Biodegradable films enriched with thymol and carvacrol with antioxidant and antibacterial properties to extend cheese shelf-life. Food Control.

[58] Teng G., Yao H., Wang H., Zhang S., Meng Y., Zhao M., Yang Y., Zhang C. (2026). Essential oil-loaded amine-modified HNTs composites for sustained release and enhanced antibacterial performance in root rot control. Appl. Clay Sci..

[59] Esquivel-Castro T.A., Robledo-Trujillo G., Oliva J., Rosu H., Rodríguez-González V. (2023). A functional SiO_2_-TiO_2_ mesoporous assembly designed for the controlled release of carvacrol. Appl. Surf. Sci. Adv..

[60] Qiu P., Ma B., Hung C.-T., Li W., Zhao D. (2019). Spherical mesoporous materials from single to multilevel architectures. Acc. Chem. Res..

[61] Ejigah V., Owoseni O., Bataille-Backer P., Ogundipe O.D., Fisusi F.A., Adesina S.K. (2022). Approaches to improve macromolecule and nanoparticle accumulation in the tumor microenvironment by the enhanced permeability and retention effect. Polymers.

[62] Fabra M.J., Flores-López M.L., Cerqueira M.A., de Rodriguez D.J., Lagaron J.M., Vicente A.A. (2016). Layer-by-layer technique to developing functional nanolaminate films with antifungal activity. Food Bioprocess Technol..

[63] Seifi S., Ghaee A. (2025). Fabrication and evaluation of pectin-gelatin skin tissue engineering scaffolds containing amoxicillin trihydrate-loaded UiO-66-NH2 nanoparticles. Int. J. Biol. Macromol..

[64] Fang W., Yu K., Zhang S., Jiang L., Zheng H., Huang Q., Li F. (2024). Shape matters: Impact of mesoporous silica nanoparticle morphology on anti-tumor efficacy. Pharmaceutics.

[65] Kingchok S., Pornsuwan S. (2020). Comparison of spherical and rod-like morphologies of SBA-15 for enzyme immobilization. J. Porous Mater..

[66] Şahin F.C., Şimşek C., Erbil C. (2025). Sulfobetaine/Alginate/Chitosan Supported Hybrid N-Isopropylacrylamide Hydrogels: Composition-Dependent Diffusion/Compression Properties and Theophylline/Diclofenac Sodium/Ciprofloxacin Release Kinetics. J. Appl. Polym. Sci..

[67] Jin X., Wang Q., Sun J., Panezai H., Bai S., Wu X. (2017). Dual (pH-and temperature-) stimuli responsive nanocarrier with bimodal mesoporous silica nanoparticles core and copolymer shell for controlled ibuprofen-releasing: Fractal feature and diffusion mechanism. Microporous Mesoporous Mater..

[68] Keawchaoon L., Yoksan R. (2011). Preparation, characterization and in vitro release study of carvacrol-loaded chitosan nanoparticles. Colloids Surf. B Biointerfaces.

[69] Ioniță S., Popescu R.-C., Irimescu I.N., Deaconu M., Tarbă N., Matei C., Mihailescu M., Savu D.-I., Berger D. (2024). Role of mesoporous silica functionalized with boronic acid derivative in targeted delivery of doxorubicin and co-delivery of doxorubicin and resveratrol. Microporous Mesoporous Mater..

[70] Walker M.J., Montemagno C.D. (1999). Sorption of *Cryptosporidium parvum* oocysts in aqueous solution to metal oxide and hydrophobic substrates. Environ. Sci. Technol..

[71] Woolsey I.D., Zeller W.E., Blomstrand B.M., Øines Ø., Enemark H.L. (2022). Effects of selected condensed tannins on *Cryptosporidium parvum* growth and proliferation in HCT-8 cell cultures. Exp. Parasitol..

[72] Alawfi B.S. (2025). Antiprotozoal Activity of Plant Extracts and their Bioactive Compounds against Cryptosporidium of Zoonotic Concern. Pak. Vet. J..

[73] Tanghort M., Chefchaou H., Mzabi A., Moussa H., Chami N., Chami F., Remmal A. (2019). Oocysticidal effect of essential oils (EOs) and their major components on *Cryptosporidium baileyi* and *Cryptosporidium galli*. Int. J. Poult. Sci..

[74] Dominguez-Uscanga A., Aycart D.F., Li K., Witola W.H., Laborde J.E.A. (2021). Anti-protozoal activity of Thymol and a Thymol ester against *Cryptosporidium parvum* in cell culture. Int. J. Parasitol. Drugs Drug Resist..

[75] Hao N., Li L., Tang F. (2017). Roles of particle size, shape and surface chemistry of mesoporous silica nanomaterials on biological systems. Int. Mater. Rev..

[76] Murugan K., Choonara Y.E., Kumar P., Bijukumar D., du Toit L.C., Pillay V. (2015). Parameters and characteristics governing cellular internalization and trans-barrier trafficking of nanostructures. Int. J. Nanomed..

[77] Nava M.G. (2024). Understanding the Molecular Mechanisms Controlling Gametogenesis and Virulence in *Cryptosporidium parvum*. Master’s Thesis.

[78] Metawae A.G., Bayoumy A.M., Ali I.R., Hammam O.A., Temsah K.A.T.A. (2021). Efficacy of nitazoxanide alone or loaded with silica nanoparticle for treatment of cryptosporidiosis in immunocompetent hosts. Int. J. Med. Arts.

[79] Docter D., Bantz C., Westmeier D., Galla H.J., Wang Q., Kirkpatrick J.C., Nielsen P., Maskos M., Stauber R.H. (2014). The protein corona protects against size-and dose-dependent toxicity of amorphous silica nanoparticles. Beilstein J. Nanotechnol..

[80] Dietz L., Simon J., Speth K.R., Landfester K., Mailänder V. (2025). Plasma protein corona on silica nanoparticles enhances exocytosis. Biomater. Sci..

[81] Sun Y., Chen D., Pan Y., Qu W., Hao H., Wang X., Liu Z., Xie S. (2019). Nanoparticles for antiparasitic drug delivery. Drug Deliv..

[82] Noble C.L., Abbas A.R., Cornelius J., Lees C.W., Ho G.-T., Toy K., Modrusan Z., Pal N., Zhong F., Chalasani S. (2008). Regional variation in gene expression in the healthy colon is dysregulated in ulcerative colitis. Gut.

[83] Tsare E.-P.G., Klapa M.I., Moschonas N.K. (2024). Protein–protein interaction network-based integration of GWAS and functional data for blood pressure regulation analysis. Hum. Genom..

[84] Gewaid H., Bowie A.G. (2024). Regulation of type I and type III interferon induction in response to pathogen sensing. Curr. Opin. Immunol..

[85] Atallah M.A., El-Kowrany S.I., Afifi O.K., Elkaliny H.H., Younis S.S., Gamea G.A. (2025). In Vivo Study on the Anti-Parasitic Effect of Omeprazole on *Cryptosporidium parvum* in Mice. Parasite Immunol..

[86] Fee B.E., Fee L.R., Menechella M., Affeldt B., Sprouse A.R., Bounini A., Alwarawrah Y., Molloy C.T., Ilkayeva O.R., Prinz J.A. (2024). Type I interferon signaling and peroxisomal dysfunction contribute to enhanced inflammatory cytokine production in IRGM1-deficient macrophages. J. Biol. Chem..

[87] Sarkar C., Lipinski M.M. (2024). Role and Function of Peroxisomes in Neuroinflammation. Cells.

[88] Kim M.E., Lee J.S. (2025). Advances in the regulation of inflammatory mediators in nitric oxide synthase: Implications for disease modulation and therapeutic approaches. Int. J. Mol. Sci..

[89] Ravi, Singh J. (2025). Redox imbalance and hypoxia-inducible factors: A multifaceted crosstalk. FEBS J..

[90] Agnihotri S., Kushwah V., Dhaked R.K. (2025). Ligand-based in silico Approach for Identifying Potent Antidotes Against Botulinum Neurotoxin Serotype A, B, E, and F. J. Curr. Toxicol. Venomics.

[91] Bello Roba B., Umar Bello A. (2025). In Silico Investigation of Antioxidant Compounds: A Comprehensive Analysis Using Docking simulations, ADME, DFT, and QSAR analysis. J. Med. Nanomater. Chem..

[92] Salarkia E., Mehdipoor M., Molaakbari E., Khosravi A., Sazegar M.R., Salari Z., Rad I., Dabiri S., Joukar S., Sharifi I. (2023). Exploring mesoporous silica nanoparticles as oral insulin carriers: In-silico and in vivo evaluation. Heliyon.

[93] Díaz-Cervantes E., Monjaraz-Rodríguez A., Aguilera-Granja F. (2022). Anti-Inflammatory Nanocarriers Based on SWCNTs and Bioactive Molecules of Oregano: An In Silico Study. Nanomanufacturing.

[94] Jamali T., Kavoosi G., Jamali Y., Mortezazadeh S., Ardestani S.K. (2021). In-vitro, in-vivo, and in-silico assessment of radical scavenging and cytotoxic activities of Oliveria decumbens essential oil and its main components. Sci. Rep..

[95] Mehmood Y., Rizvi S.M., Ijaz M., Shahzadi T., Shahid H., Ahmed S., Rasul A., Iqbal J., Almehizia A.A., Bouallegue A. (2025). A novel pregabalin functionalized salicylaldehyde derivative loaded mesoporous silica nano scaffold: A prospective carrier for targeting inflammatory cytokine storm. BMC Biotechnol..

[96] Naghavi F., Morsali A., Bozorgmehr M.R. (2019). Molecular mechanism study of surface functionalization of silica nanoparticle as an anticancer drug nanocarrier in aqueous solution. J. Mol. Liq..

[97] Das R., Vigneresse J.-L., Chattaraj P.K. (2013). Redox and Lewis acid–base activities through an electronegativity-hardness landscape diagram. J. Mol. Model..

[98] Filhol J.-S., Doublet M.-L. (2014). Conceptual surface electrochemistry and new redox descriptors. J. Phys. Chem. C.

[99] Muñoz-Caro T., Lendner M., Daugschies A., Hermosilla C., Taubert A. (2015). NADPH oxidase, MPO, NE, ERK1/2, p38 MAPK and Ca^2+^ influx are essential for *Cryptosporidium parvum*-induced NET formation. Dev. Comp. Immunol..

[100] Asogwa F.C., Apebende C.G., Ugodi G.W., Ebo P., Louis H., Ikeuba A.I., Asogwa C.J., Gber T.E., Ikot I.J., Owen A.E. (2023). Anti-inflammatory, immunomodulatory and DFT evaluation of the reactivity indexes of phytochemicals isolated from Harungana madagascariensis. Chem. Afr..

[101] Unsal V., Ercan L., Calıskan C.G. (2025). Determination of bioactive and anti-inflammatory molecules of Thymbra spicata L. from Mardin by GC–MS and LC–Orbitrap HRMS: A DFT, molecular docking, ADMET, biological target and activity study. BMC Complement. Med. Ther..

[102] de Oliveira A.S., Llanes L.C., Nunes R.J., Nucci-Martins C., de Souza A.S., Palomino-Salcedo D.L., Dávila-Rodríguez M.J., Ferreira L.L., Santos A.R., Andricopulo A.D. (2021). Antioxidant activity, molecular docking, quantum studies and in vivo antinociceptive activity of sulfonamides derived from carvacrol. Front. Pharmacol..

[103] Boulebd H. (2019). DFT study of the antiradical properties of some aromatic compounds derived from antioxidant essential oils: C–H bond vs. O–H bond. Free Radic. Res..

[104] Zamani M., Delfani A.M., Jabbari M. (2018). Scavenging performance and antioxidant activity of γ-alumina nanoparticles towards DPPH free radical: Spectroscopic and DFT-D studies. Spectrochim. Acta Part A Mol. Biomol. Spectrosc..

[105] Surendar A., Sadulla S., Fajer A.N., Abbas M., Najafi M. (2019). Anti-inflammatory activity of synthesized diarylpentenedione derivatives and their drug delivery with silicon-nanotube (7,7). Curr. Sci..

[106] Liang X., Wang Y., Li T., Li P., Jiang G. (2025). Mechanistic Study on the Alleviation of Endometritis in Mice Through Inhibition of NF-κB and MAPK Signaling Pathways by Berberine and Carvacrol. Microorganisms.

[107] Lean I.-S., McDonald V., Pollok R.C. (2002). The role of cytokines in the pathogenesis of Cryptosporidium infection. Curr. Opin. Infect. Dis..

[108] Zhang G., Zhang Y., Niu Z., Wang C., Xie F., Li J., Zhang S., Qi M., Jian F., Ning C. (2020). Vectors, *Cryptosporidium parvum* upregulates miR-942-5p expression in HCT-8 cells via TLR2/TLR4-NF-κB signaling. Parasites Vectors.

[109] Sivamaruthi B.S., Thangaleela S., Kesika P., Suganthy N., Chaiyasut C. (2023). Mesoporous silica-based nanoplatforms are theranostic agents for the treatment of inflammatory disorders. Pharmaceutics.

[110] Xiong Y., Zhang Y., Zhou C., Yu T. (2023). ROS scavenging Manganese-loaded mesoporous silica nanozymes for catalytic anti-inflammatory therapy. Adv. Powder Technol..

[111] Somensi N., Rabelo T.K., Guimarães A.G., Quintans-Junior L.J., de Souza Araújo A.A., Moreira J.C.F., Gelain D.P. (2019). Carvacrol suppresses LPS-induced pro-inflammatory activation in RAW 264.7 macrophages through ERK1/2 and NF-kB pathway. Int. Immunopharmacol..

[112] Wang T., He C. (2020). TNF-α and IL-6: The link between immune and bone system. Curr. Drug Targets.

[113] Malemud C. (2010). Differential activation of JAK enzymes in rheumatoid arthritis and autoimmune disorders by pro-inflammatory cytokines: Potential drug targets. Int. J. Interf. Cytokine Mediat. Res..

[114] Christen V., Camenzind M., Fent K. (2014). Silica nanoparticles induce endoplasmic reticulum stress response, oxidative stress and activate the mitogen-activated protein kinase (MAPK) signaling pathway. Toxicol. Rep..

[115] Yang H., Zhang Y., Li W., Lao C., Li M., Zheng Y. (2017). Altered microRNA expression profiles in lung damage induced by nanosized SiO_2_. Bioengineered.

[116] Panas A., Marquardt C., Nalcaci O., Bockhorn H., Baumann W., Paur H.-R., Mülhopt S., Diabaté S., Weiss C. (2013). Screening of different metal oxide nanoparticles reveals selective toxicity and inflammatory potential of silica nanoparticles in lung epithelial cells and macrophages. Nanotoxicology.

[117] Winfree R.L., Seto M., Dumitrescu L., Menon V., De Jager P., Wang Y., Schneider J., Bennett D.A., Jefferson A.L., Hohman T.J. (2023). TREM2 gene expression associations with Alzheimer’s disease neuropathology are region-specific: Implications for cortical versus subcortical microglia. Acta Neuropathol..

[118] Bencze J., Szarka M., Kóti B., Seo W., Hortobágyi T.G., Bencs V., Módis L.V., Hortobágyi T. (2021). Comparison of semi-quantitative scoring and artificial intelligence aided digital image analysis of chromogenic immunohistochemistry. Biomolecules.

[119] Mahran O.M., Rateb M.H., Abouel-Hassan L., Abd Allah E.A. (2020). Oxidative Stress Biomarkers and Pathological Alterations Induced by Cryptosporidim Infection in Buffalo Calves at Assiut Governorate, Egypt. J. Adv. Vet. Res..

[120] Tekin S., Seven E. (2022). Assessment of serum catalase, reduced glutathione, and superoxide dismutase activities and malondialdehyde levels in keratoconus patients. Eye.

[121] Dong W., Su J., Chen Y., Xu D., Cheng L., Mao L., Gao Y., Yuan F. (2022). Characterization and antioxidant properties of chitosan film incorporated with modified silica nanoparticles as an active food packaging. Food Chem..

[122] Huang X., Zhuang J., Teng X., Li L., Chen D., Yan X., Tang F. (2010). The promotion of human malignant melanoma growth by mesoporous silica nanoparticles through decreased reactive oxygen species. Biomaterials.

[123] Hozayen W.G., Mahmoud A.M., Desouky E.M., El-Nahass E.-S., Soliman H.A., Farghali A.A. (2019). Cardiac and pulmonary toxicity of mesoporous silica nanoparticles is associated with excessive ROS production and redox imbalance in Wistar rats. Biomed. Pharmacother..

[124] Xu L., Yang X., Liu X.-T., Li X.-Y., Zhu H.-Z., Xie Y.-H., Wang S.-W., Li Y., Zhao Y. (2024). Carvacrol alleviates LPS-induced myocardial dysfunction by inhibiting the TLR4/MyD88/NF-κB and NLRP3 inflammasome in cardiomyocytes. J. Inflamm..

