# Supplementary material for: Therapeutic Efficacy of Carvacrol-Loaded Mesoporous Silicate Nanoparticles Against Cryptosporidiosis
Source: Pharmaceutics. 2026 Aug 26;18(9):1065. doi: 10.3390/pharmaceutics18091065 (PMC13610952; doi:10.3390/pharmaceutics18091065)
Supplement: Supplementary file 1 [file pharmaceutics-18-01065-s001.zip › pharmaceutics-4461960-supplementary.pdf]

Therapeutic Efficacy of Carvacrol—Loaded Mesoporous Silicate Nanoparticles Against Cryptosporidiosis

Moataz M. Rashad [1,2,*], Shaimaa M. Kasem [3], Khaled E. El-Kelany [1,4] and Sara A. Abdel Gaber [2,5*]

[1] Institute of Nanoscience and Nanotechnology, Kafrelsheikh University, Kafrelsheikh 33516, Egypt
[2] Nanomedicine Department, Institute of Nanoscience and Nanotechnology, Kafrelsheikh University, Kafrelsheikh 33516, Egypt
[3] Zoology Department, Faculty of Science, Kafrelsheikh University, Kafrelsheikh 33516, Egypt
[4] Chemistry Department, Faculty of Science,University of Sadat City, Sadat City 32897, Egypt
[5] Clinical Pharmacy Department, Faculty of Pharmacy, Egypt-Japan University of Science and Technology, New Borg El-Arab, Alexanderia, Egypt
* Correspondence: motaz_pg031@nano.kfs.edu.eg (M.M.R.); sara_mohamed@nano.kfs.edu.eg (S.A.A.G.)

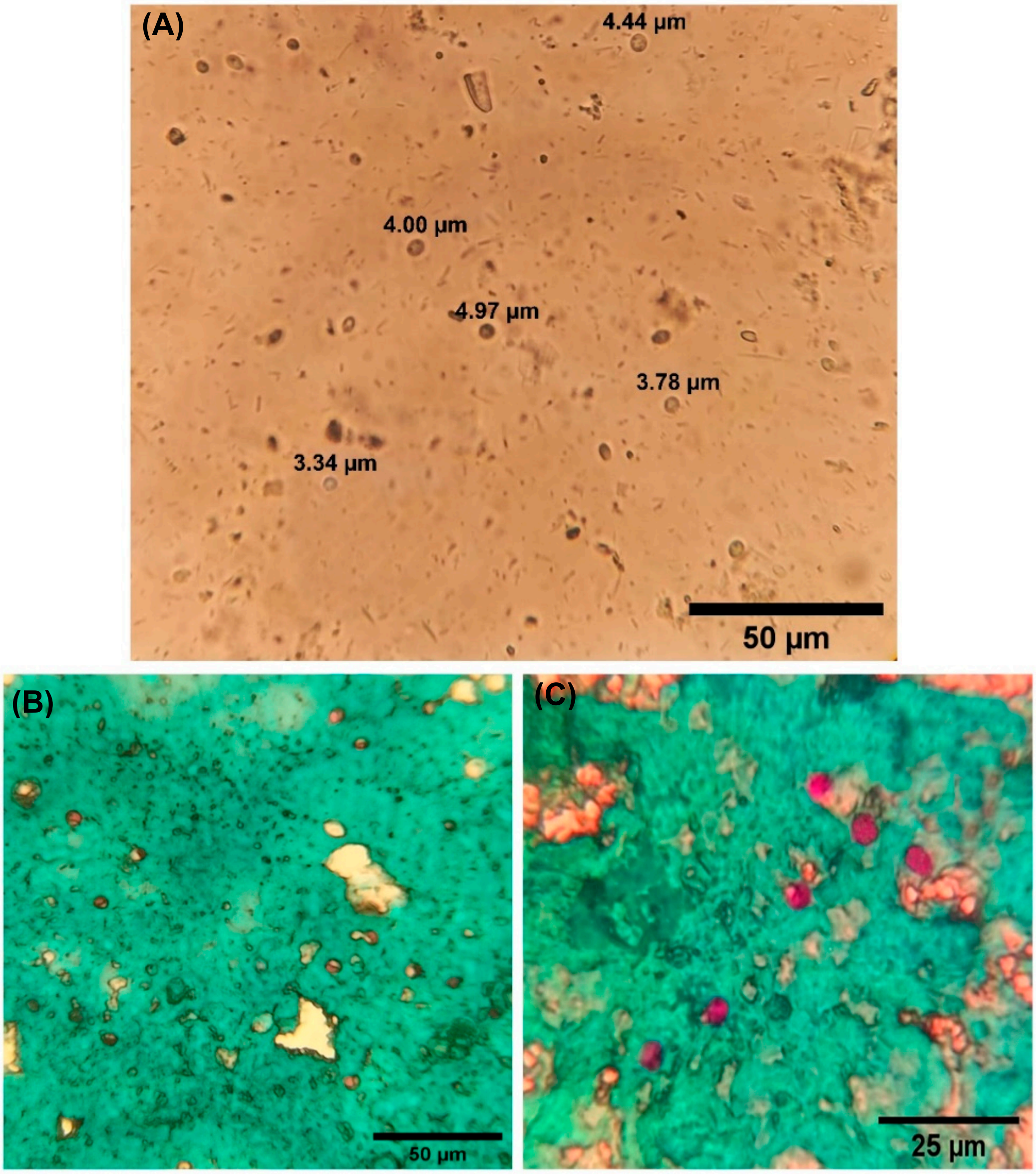

Figure S1. Characterization of unstained *C. parvum oocysts* in a direct wet mount. Scale bar: 50 µm (A) & Stained *C. parvum* oocysts with modified Ziehl Neelsen. Oocysts are round to oval in shape with a pinkish red color encountered with a blue green background. **(B)** low magnification, Scale bar: 50 µm and **(C)** high magnification, Scale bar: 25 µm.

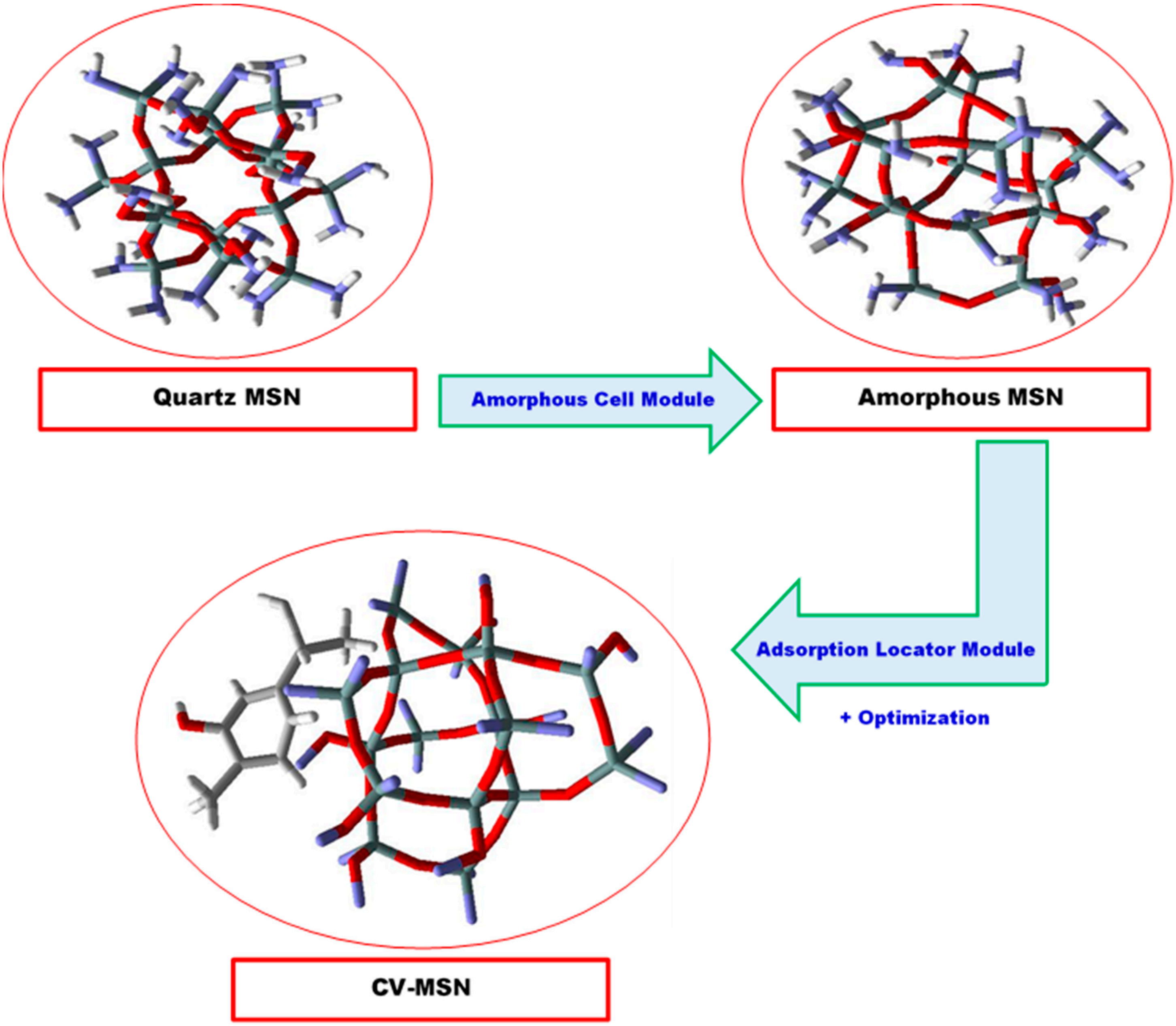

Scheme S1. schematic figure showing ligand preparation steps

Table S1. Primers sequence of all studied genes in qRT-PCR.

Target Gene	Forward sequence	Reverse sequence	Accession NO.
iNOS	ATGGACCAGTATAAGGCAAGC	GCTCTGGATGAGCCTATATTG	NM_010927.4
NFκB P65	CTGCGATACCTTAATGACAGCG	AATTTGGCTTCCTTTCTTGGCT	NM_001276711.1
JAK-1	ACCAGGCAAGATCCAGACAT	TCTTCAGGAACCGCTTTTCA	NM_013567.1
STAT3	CCCGTACCTGAAGACCAAG	CGAGGTCAGATCCATGTCAA	NM_213659.4
TLR4	CCGTCACCACATACTGCCTTTA	GCAGTTTGGACTATTGAAATACGAAA	NM_001109668.1
P38-MAPK	CACAGCACCTCAGCAATGAT	AGGCCTATCTTCCCAGGAAA	NM_001357115.1
GAPDH	TGTGTCCGTCGTGGATCTGA	CCTGCTTCACCACCTTCTTGAT	NM_001411845.1

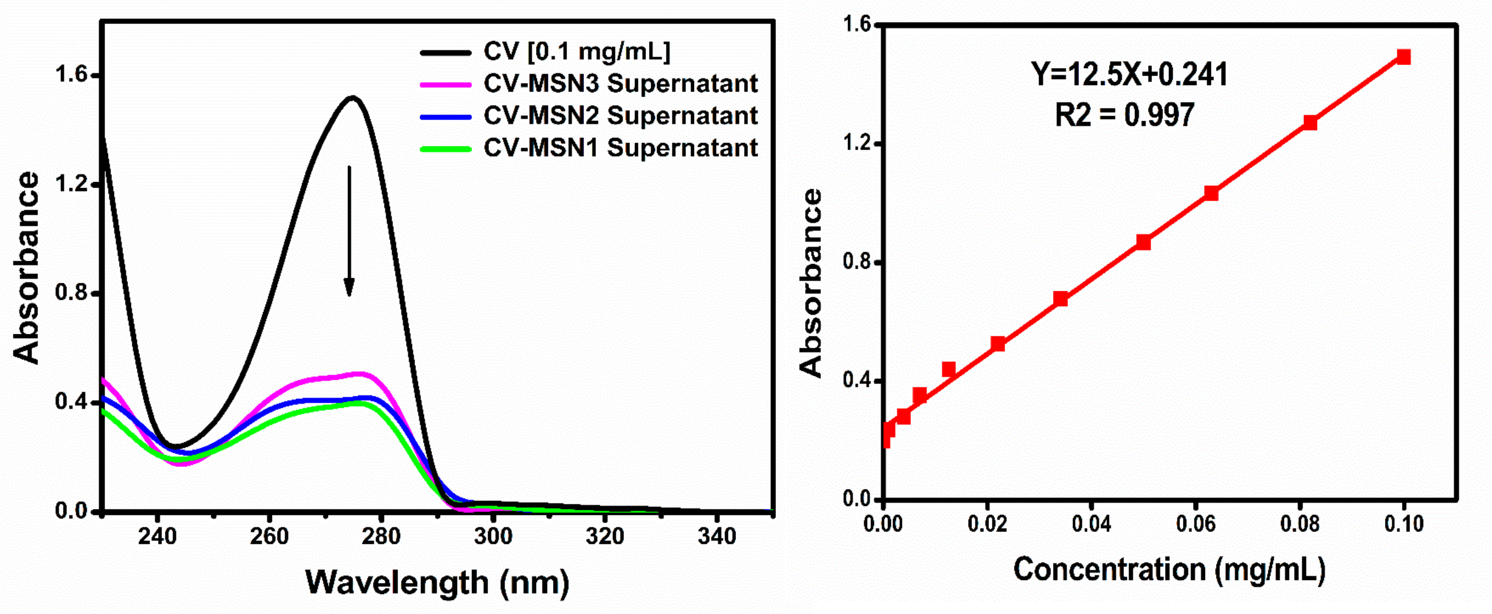

Figure S2. Loading efficiency experiment; (A) UV-vis spectra of free CV and supernatants collected from CV-MSN formulations and (B) Calibration curve of known concentrations of CV.

Table S2. The analytical validation parameters of UV-vis spectra of CV and calibration curve for loading efficiency calculation

Parameter	The calculated value
Regression Equation	Y=12.5X+0.241
Correlation Coefficient (R2)	0.997
Slope	12.5
Intercept	0.241 ± 0.00158
Limit of Detection (LOD)	0.417 µg/mL
Limit of quantification (LOQ)	1.26 µg/mL

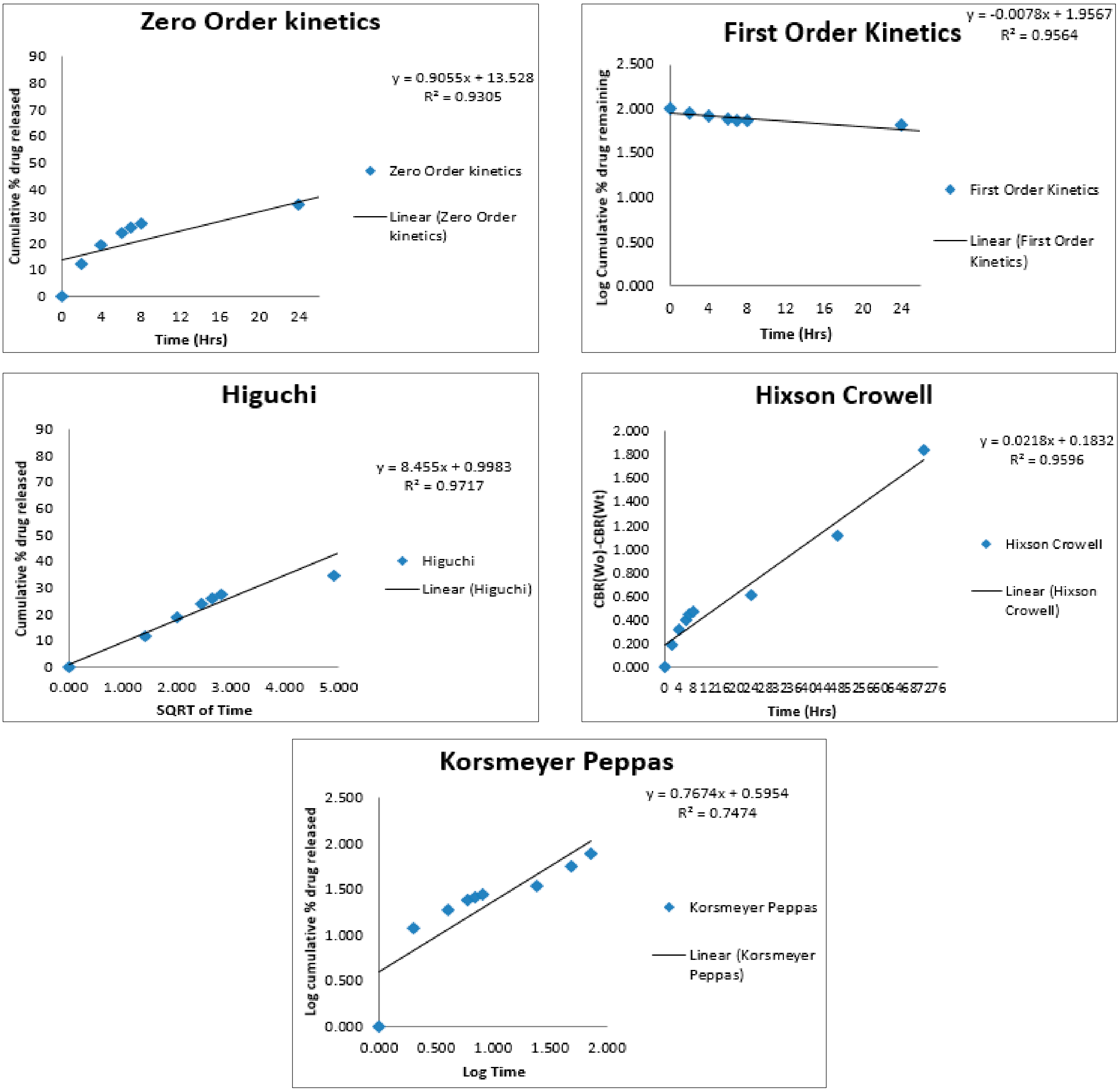

Figure S3. Drug release kinetics plots of CV-MSN1

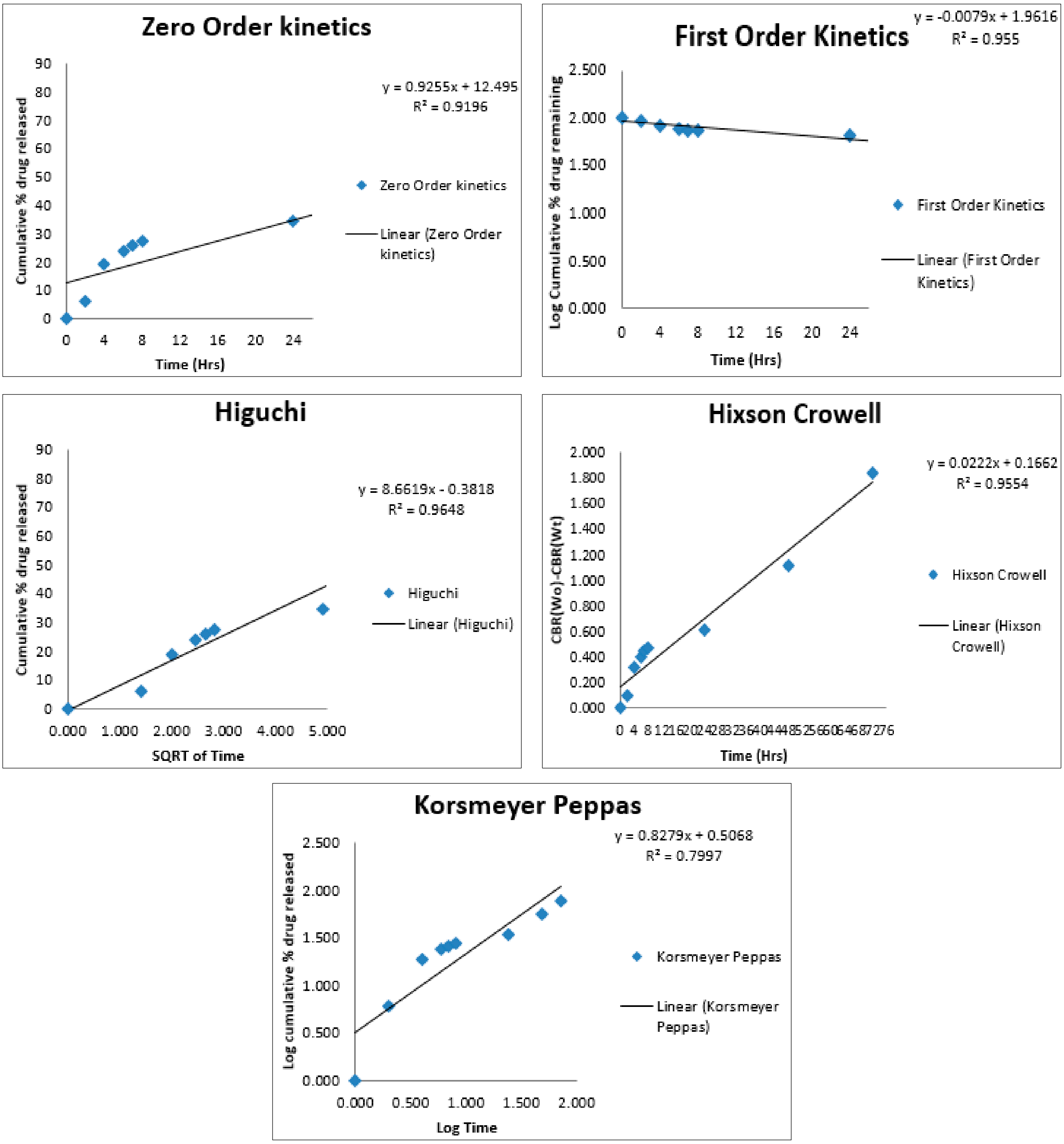

Figure S4. Drug release kinetics plots of CV-MSN2

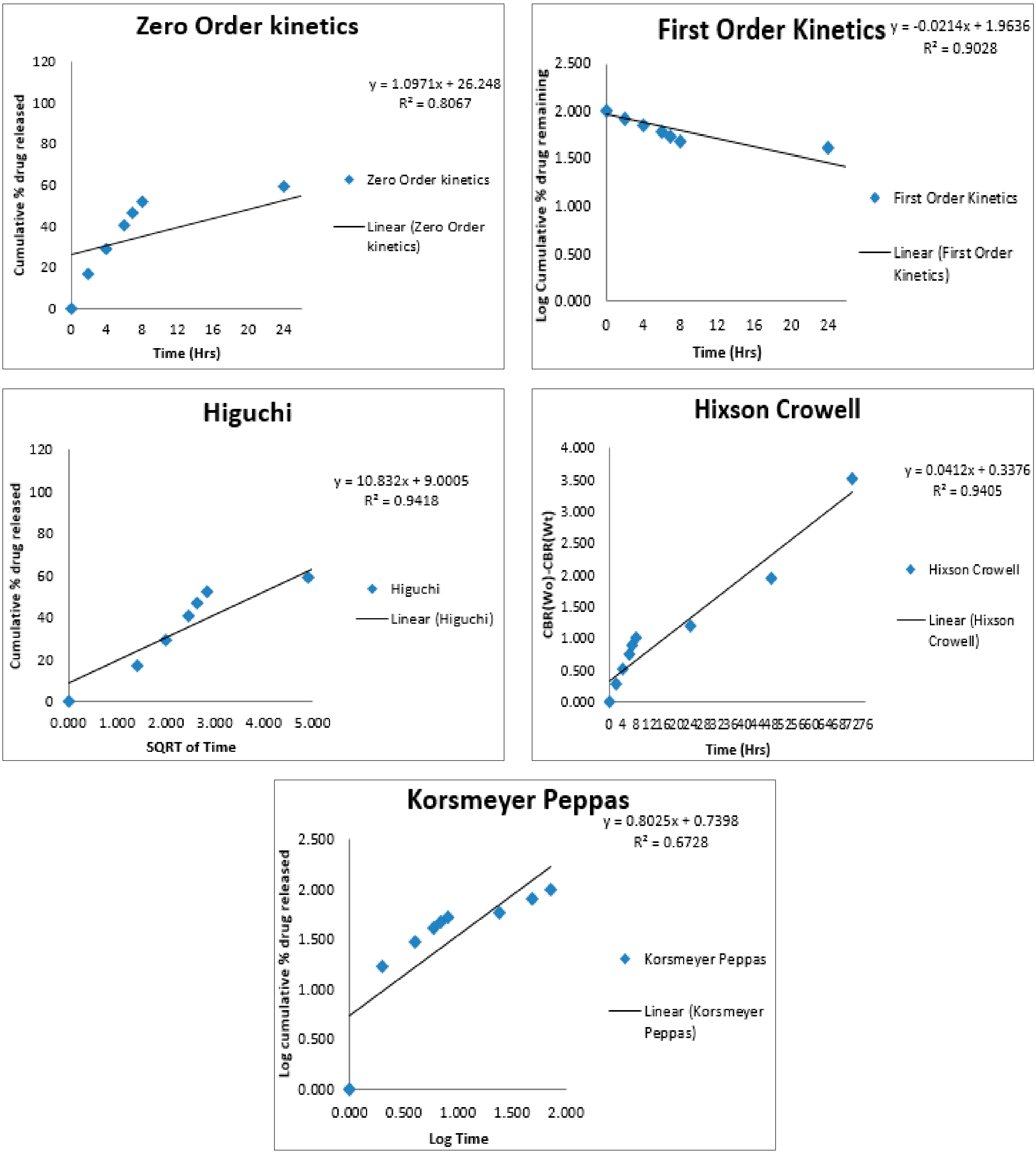

Figure S5. Drug release kinetics plots of CV-MSN3

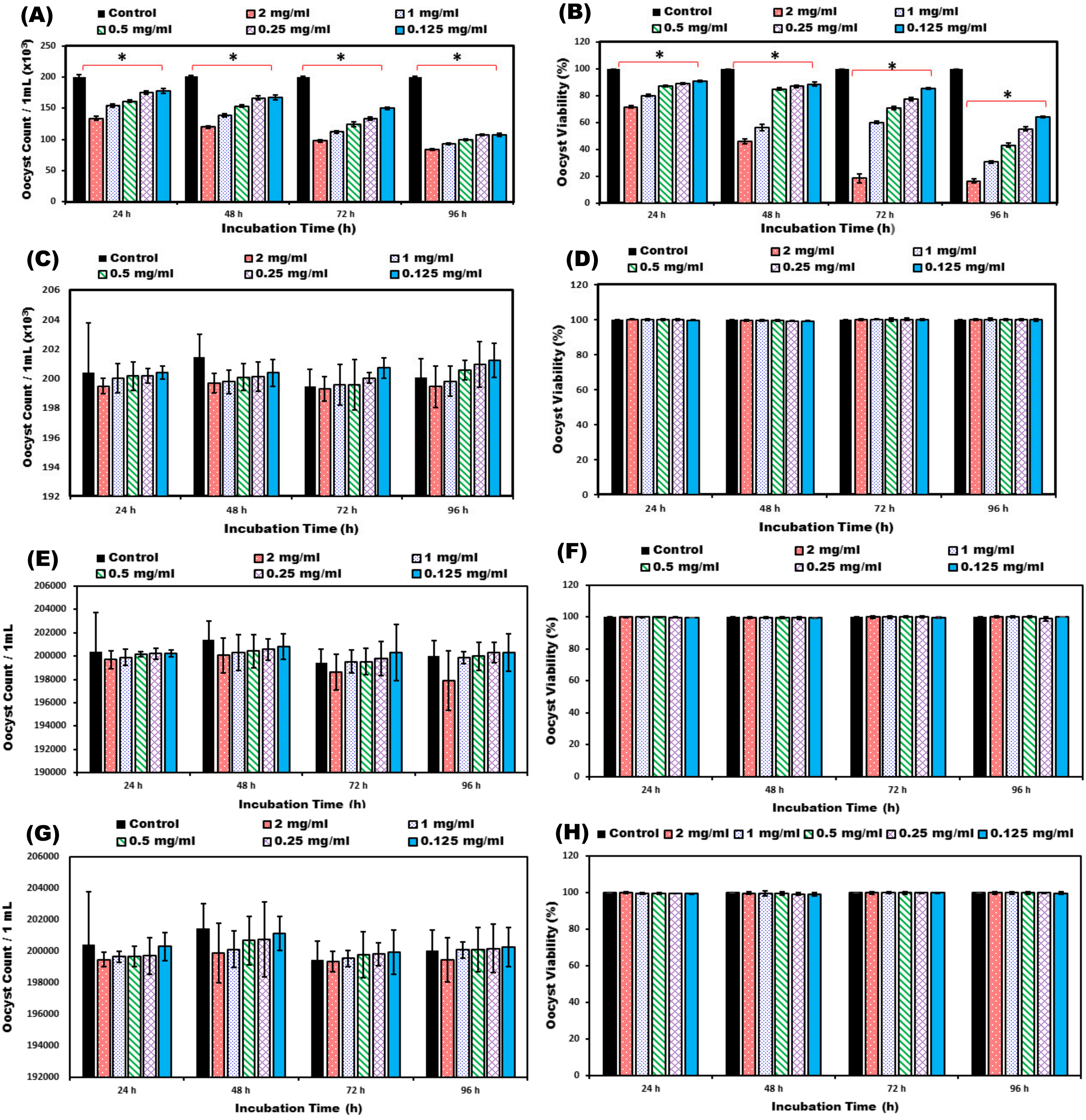
(A)
Control
2 mg/ml
1 mg/ml
0.5 mg/ml
0.25 mg/ml
0.125 mg/ml
Oocyst Count / 1mL (x10^3)
24 h
48 h
72 h
96 h
Incubation Time (h)
(B)
Oocyst Viability (%)
Incubation Time (h)
(C)
Oocyst Count / 1mL (x10^3)
Incubation Time (h)
(D)
Oocyst Viability (%)
Incubation Time (h)
(E)
Oocyst Count / 1mL
Incubation Time (h)
(F)
Oocyst Viability (%)
Incubation Time (h)
(G)
Oocyst Count / 1 mL
Incubation Time (h)
(H)
Oocyst Viability (%)
Incubation Time (h)

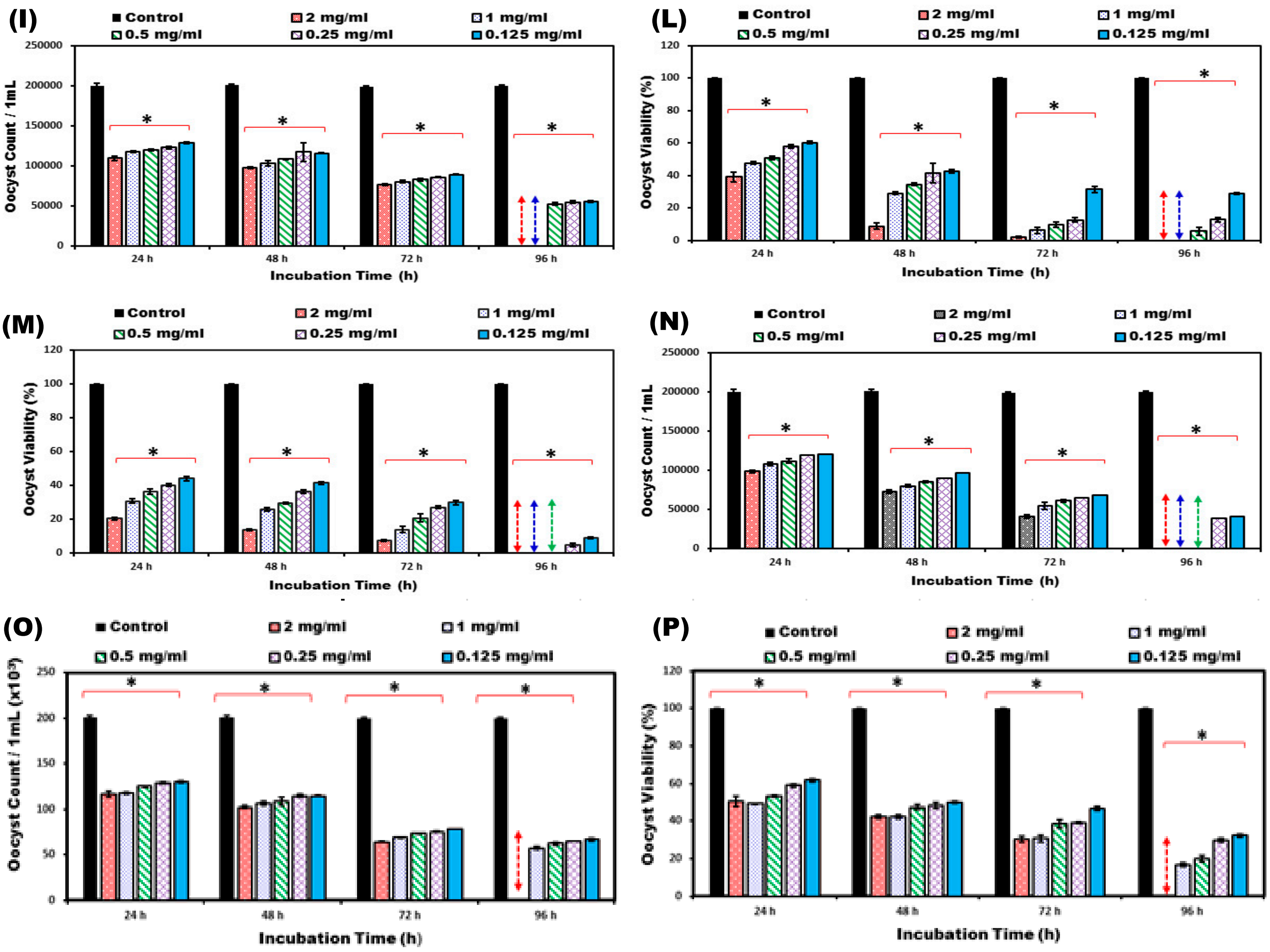

Figure S6. Oocyst count and viability percentages after incubation of free CV (A-B), free MSN1 (C-D), free MSN2 (E-F), free MSN3 (G-H), CV-MSN1 (I-L), CV-MSN2 (M-N) and CV-MSN3 (O-P). Data are shown as means ± SD. *Significant at P< 0.05 against Negative control.

Table S3. The calculated LC50 during the *in vitro* anti-oocysticidal study by non-linear regression analysis

Substance	LC50 (mg/mL)			
	**24 h**	**48 h**	**72 h**	**96 h**
Free CV	**NA**	**0.58**	**0.24**	**0.10**
Free MSN1	**NA**			
Free MSN2	**NA**			
Free MSN3	**NA**			
CV-MSN1	**0.66**	**0.11**	**0.09**	**0.088**
CV-MSN2	**0.11**	**0.10**	**0.09**	**0.07**
CV-MSN3	**0.90**	**0.13**	**0.12**	**0.09**

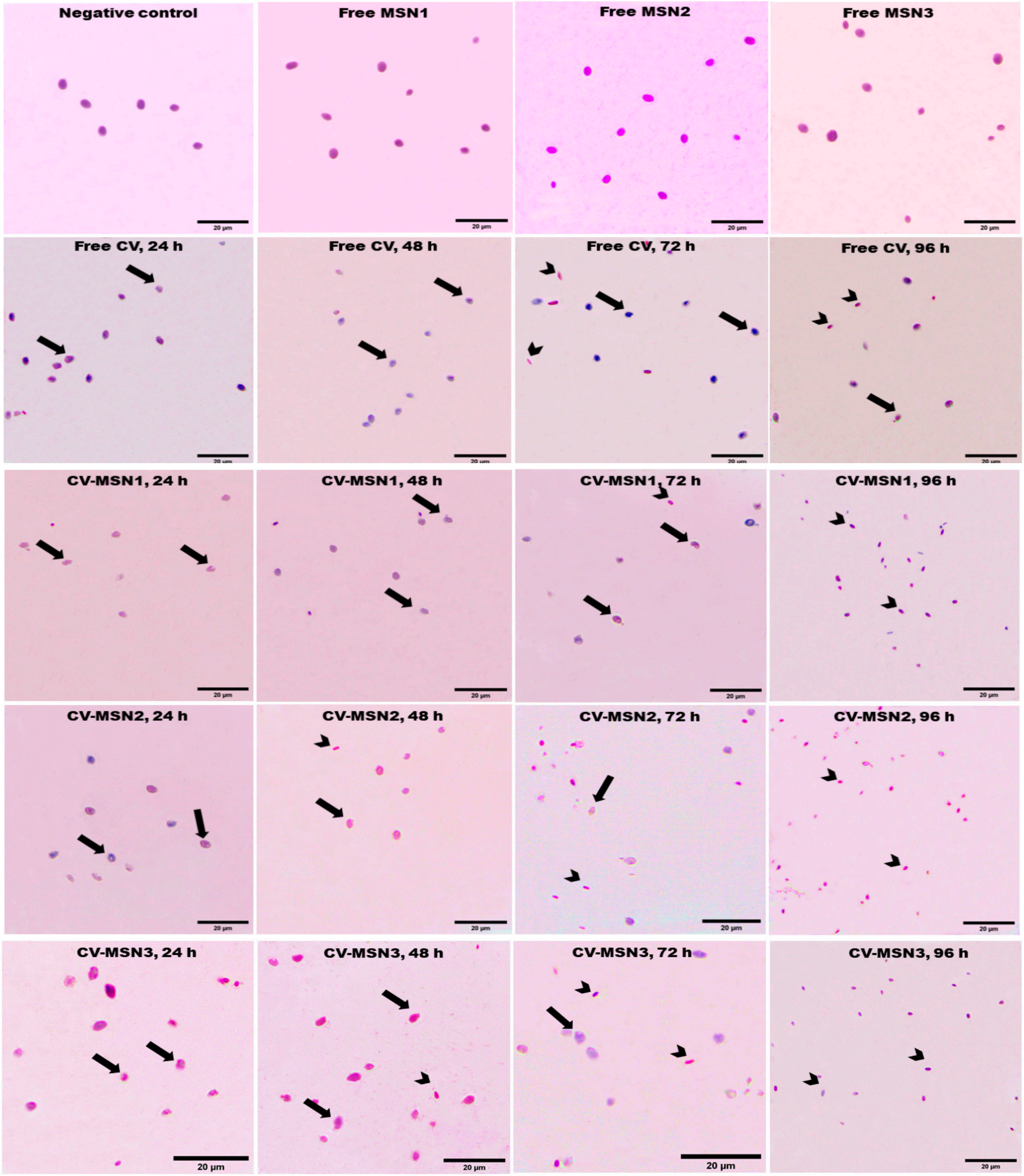

Figure S7. Photomicrographs of *C. parvum* oocysts treated with 2 mg/ml of all formulations for 96 h. arrows; damaged oocysts, arrowheads; liberated sporozoites. Scale Bar= 20 µm.

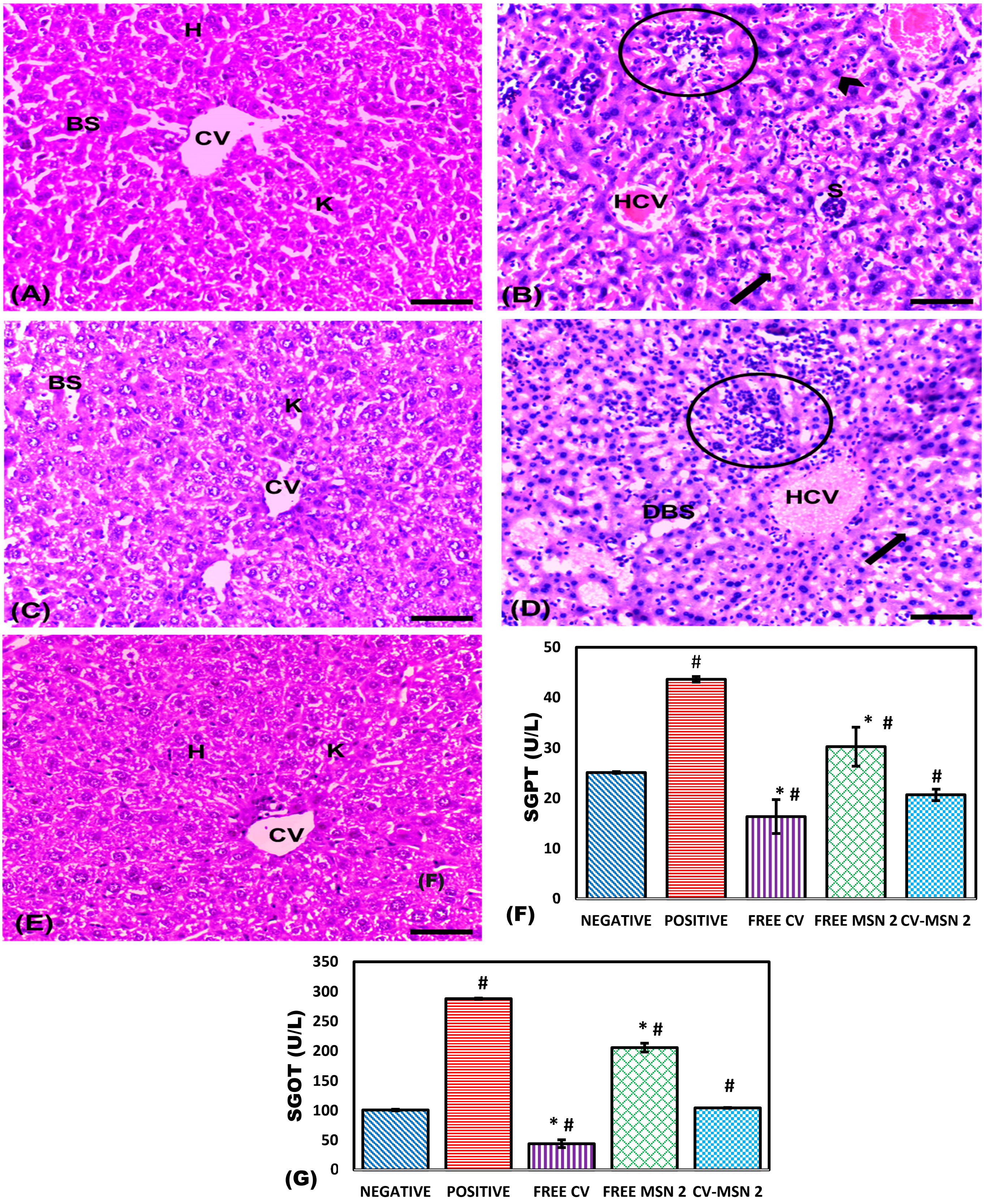
H
BS
CV
K
(A)
HCV
S
(B)
BS
K
CV
(C)
DBS
HCV
(D)
H
K
CV
(F)
(E)
SGPT (U/L)
50
40
30
20
10
0
#
* #
* #
#
NEGATIVE
POSITIVE
FREE CV
FREE MSN 2
CV-MSN 2
(F)
SGOT (U/L)
350
300
250
200
150
100
50
0
#
* #
* #
#
NEGATIVE
POSITIVE
FREE CV
FREE MSN 2
CV-MSN 2
(G)

Figure S8. Microscopic photographs of H&E stained hepatic tissue of *C. parvum* infected mice. **(A)** Negative control showing normal hepatic architecture with well-arranged hepatocytes (H) around a central vein (CV). Intact blood sinusoids (BS) were clearly visible between the hepatocyte plates lined by endothelial and Kupffer cells (K). **(B)** Positive control exhibiting hepatocellular degeneration characterized by cell swelling (S), cytoplasmic vacuolation (arrow), and nuclear pyknosis (arrowhead). Hemorrhaged central vein (HCV) and focal inflammatory cell infiltrates (circle) were also seen. **(C)** Free CV treated group demonstrating moderate restoration of hepatic structure with reduced inflammatory infiltration. **(D)** Free MSN2 treated group indicating damaged hepatocytes with hemorrhaged central vein (HCV) and focal inflammatory cell infiltrates (circle) and dilated blood sinusoids (DBS). **(E)** CV-MSN2 treated group displaying semi-normal architecture, with well-organized hepatocytes (H), intact sinusoids, and a normal-appearing central vein (CV) and Kupffer cells (K). Scale bar = 100 µm. (F-G) liver functions analysis after treatment. Data are shown as means ± SD. *Significant at $P< 0.05$ against Negative control. $^\#$Significant at $P< 0.05$ against Positive control.

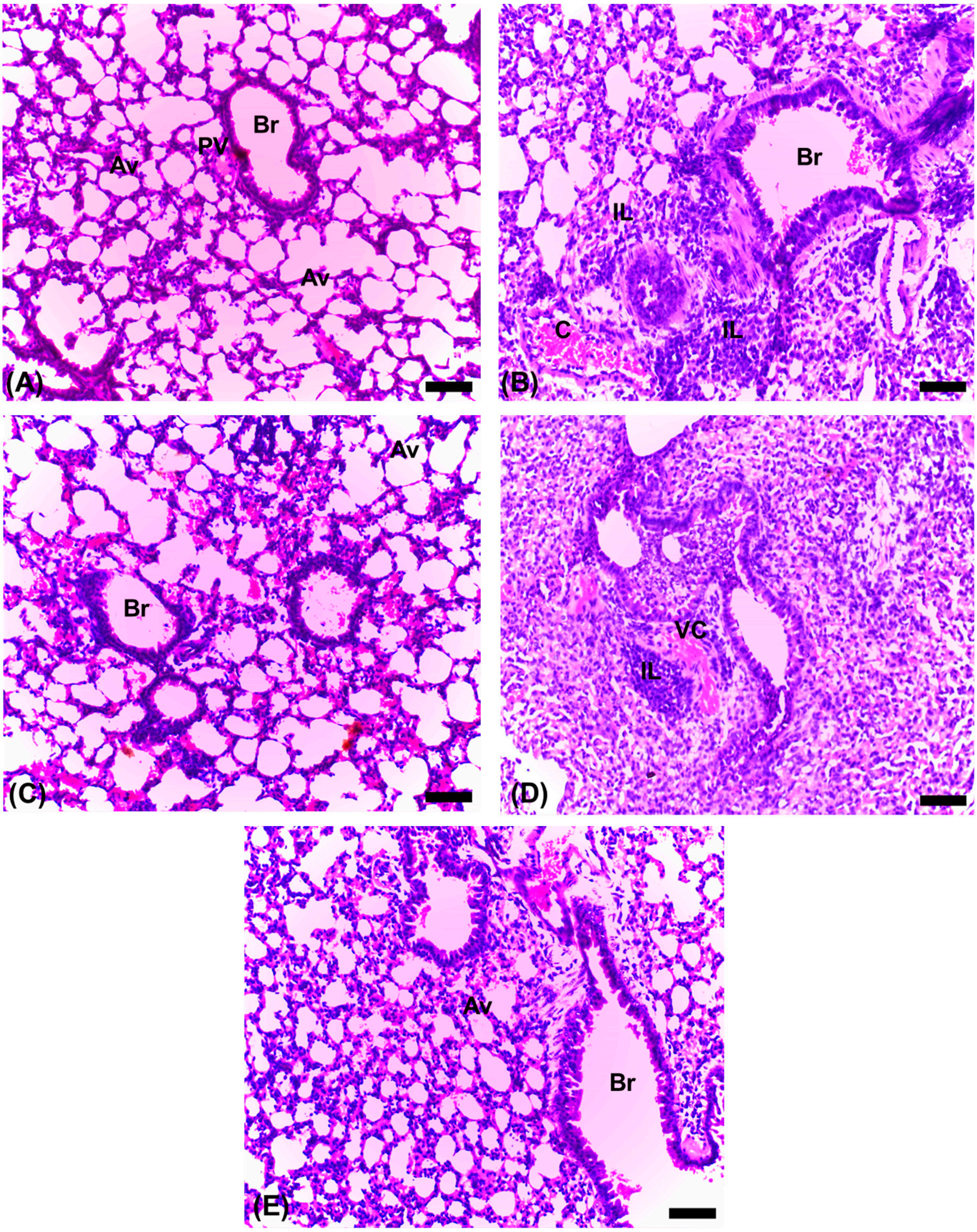

Figure S9. Microscopic photographs of H&E stained lung tissue of *C. parvum* infected mice. **(A)** Negative control revealing intact bronchiolar (Br) structure with epithelial lining. The alveoli (Av) and alveolar spaces appear mostly open and well aerated. Pulmonary vessel (PV) was also seen. **(B)** Positive control showing dense inflammatory infiltration filling with lymphocytes (IL) and expanded interstitial and peribronchiolar regions. The bronchiole appeared hyperplastic and thickened, with appeared congested (C) regions. Several bronchiolar structures appear narrowed or partially

obstructed by the surrounding inflammation. **(C)** Free CV treated group showing substantially preserved pulmonary architecture, characterized by well-expanded alveolar spaces and only moderate interstitial thickening**. (D)** Free MSN2 treated group demonstrating pronounced inflammatory infiltration with lymphocytes (IL), with dense peribronchiolar inflammation and infiltrating the adjacent alveolar spaces as well as vascular congestion (VC). Surrounding tissue demonstrated interstitial expansion, cellular infiltration, and areas of alveolar collapse or reduced aeration were seen. **(E)** CV-MSN2 treated group indicating markedly improved lung architecture with opened and well aerated alveoli and alveolar spaces, and only mild interstitial thickening. The bronchiolar structure was intact, and there was no obvious heavy inflammatory infiltration. Scale bar = 100 µm.

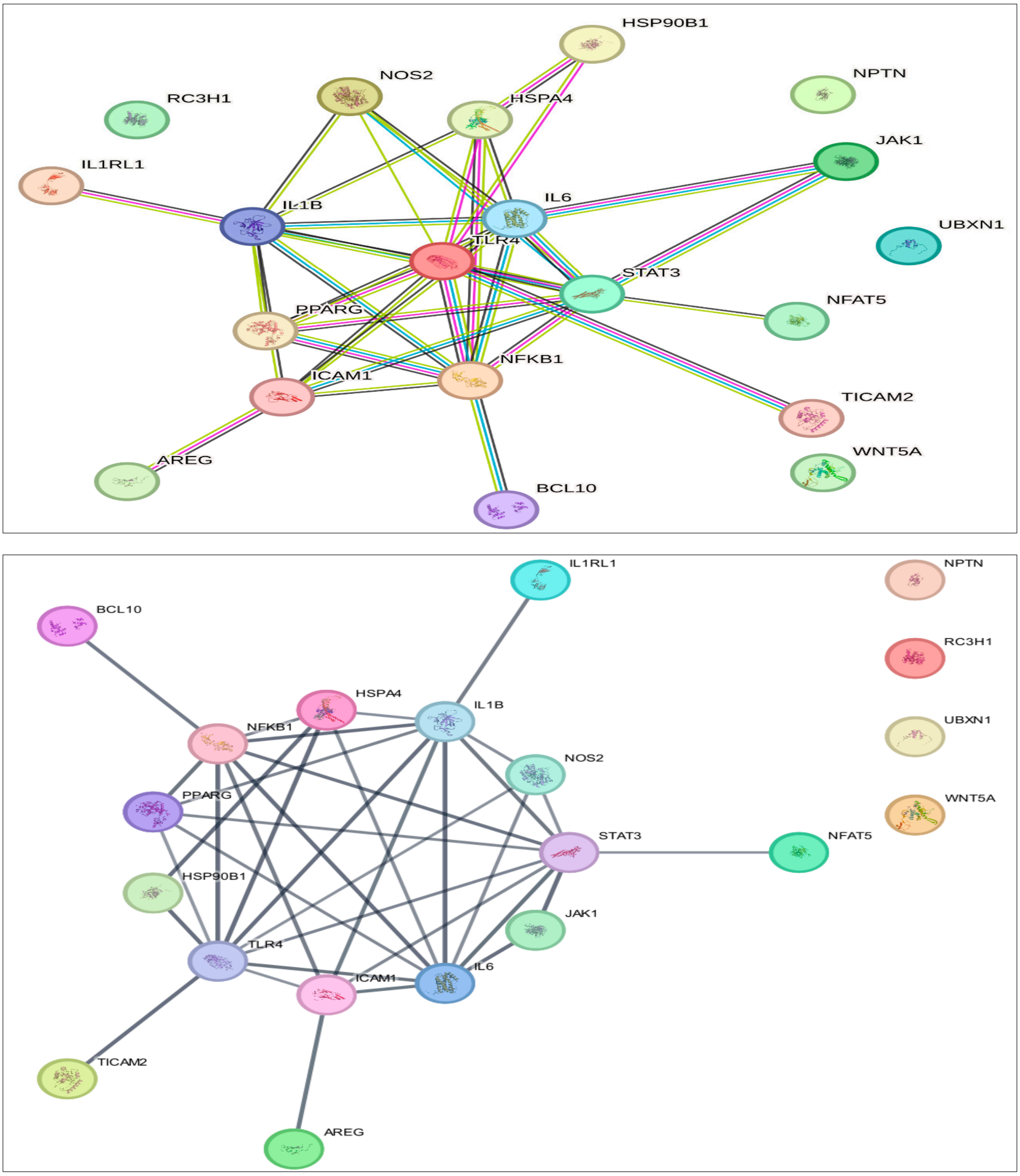

Figure S10. PPI network of the target proteins. (A) Network showing interactions among proteins with confidence score ≥ 0.700 created by The STRING database. (B) Circular representation of the same network visualized by cytoscape 3.10.4. Spherical nodes represent target proteins, and grey lines indicate protein-protein interactions. Line thickness corresponds to the confidence or probability of each interaction. The disconnected nodes represent the proteins have no interactions in the PPI network.

Table S4. Top 10 in network STRING network ranked by Degree method

Rank	Name	Gene Symbol	Degree Score
1	9606.ENSP00000363089	TLR4	10
2	9606.ENSP00000385675	IL-6	9
2	9606.ENSP00000263341	IL-1β	9
2	9606.ENSP00000264657	STAT-3	9
5	9606.ENSP00000226574	NF-κB	8
6	9606.ENSP00000264832	ICAM-1	6
7	9606.ENSP00000302961	HSPA4	5
7	9606.ENSP00000287820	PPARG	5
9	9606.ENSP00000327251	NOS2	4
10	9606.ENSP00000499900	JAK-1	2

Table S5. Main predicted target interaction

Node1	Node2	Node1 accession	Node2 accession	Combined score
AREG	ICAM1	ENSP00000379097	ENSP00000264832	0.947
BCL10	NFKB1	ENSP00000498104	ENSP00000226574	0.929
HSP90B1	HSPA4	ENSP00000299767	ENSP00000302961	0.99
HSP90B1	TLR4	ENSP00000299767	ENSP00000363089	0.988
HSPA4	HSP90B1	ENSP00000302961	ENSP00000299767	0.99
HSPA4	IL1B	ENSP00000302961	ENSP00000263341	0.776
HSPA4	IL6	ENSP00000302961	ENSP00000385675	0.79
HSPA4	NFKB1	ENSP00000302961	ENSP00000226574	0.857
HSPA4	TLR4	ENSP00000302961	ENSP00000363089	0.996
ICAM1	AREG	ENSP00000264832	ENSP00000379097	0.947
ICAM1	IL1B	ENSP00000264832	ENSP00000263341	0.912
ICAM1	IL6	ENSP00000264832	ENSP00000385675	0.947
ICAM1	NFKB1	ENSP00000264832	ENSP00000226574	0.903
ICAM1	STAT3	ENSP00000264832	ENSP00000264657	0.832
ICAM1	TLR4	ENSP00000264832	ENSP00000363089	0.768
IL1B	HSPA4	ENSP00000263341	ENSP00000302961	0.776
IL1B	ICAM1	ENSP00000263341	ENSP00000264832	0.912
IL1B	IL1RL1	ENSP00000263341	ENSP00000233954	0.892
IL1B	IL6	ENSP00000263341	ENSP00000385675	0.996
IL1B	NFKB1	ENSP00000263341	ENSP00000226574	0.996
IL1B	NOS2	ENSP00000263341	ENSP00000327251	0.803
IL1B	PPARG	ENSP00000263341	ENSP00000287820	0.858
IL1B	STAT3	ENSP00000263341	ENSP00000264657	0.938
IL1B	TLR4	ENSP00000263341	ENSP00000363089	0.986
IL1RL1	IL1B	ENSP00000233954	ENSP00000263341	0.892
IL6	HSPA4	ENSP00000385675	ENSP00000302961	0.79
IL6	ICAM1	ENSP00000385675	ENSP00000264832	0.947
IL6	IL1B	ENSP00000385675	ENSP00000263341	0.996
IL6	JAK1	ENSP00000385675	ENSP00000499900	0.996
IL6	NFKB1	ENSP00000385675	ENSP00000226574	0.994
IL6	NOS2	ENSP00000385675	ENSP00000327251	0.781
IL6	PPARG	ENSP00000385675	ENSP00000287820	0.82
IL6	STAT3	ENSP00000385675	ENSP00000264657	0.984
IL6	TLR4	ENSP00000385675	ENSP00000363089	0.973
JAK1	IL6	ENSP00000499900	ENSP00000385675	0.996
JAK1	STAT3	ENSP00000499900	ENSP00000264657	0.999
NFAT5	STAT3	ENSP00000338806	ENSP00000264657	0.748
NFKB1	BCL10	ENSP00000226574	ENSP00000498104	0.929
NFKB1	HSPA4	ENSP00000226574	ENSP00000302961	0.857
NFKB1	ICAM1	ENSP00000226574	ENSP00000264832	0.903
NFKB1	IL1B	ENSP00000226574	ENSP00000263341	0.996
NFKB1	IL6	ENSP00000226574	ENSP00000385675	0.994
NFKB1	PPARG	ENSP00000226574	ENSP00000287820	0.972
NFKB1	STAT3	ENSP00000226574	ENSP00000264657	0.962
NFKB1	TLR4	ENSP00000226574	ENSP00000363089	0.993
NOS2	IL1B	ENSP00000327251	ENSP00000263341	0.803

NOS2	IL6	ENSP00000327251	ENSP00000385675	0.781
NOS2	STAT3	ENSP00000327251	ENSP00000264657	0.749
NOS2	TLR4	ENSP00000327251	ENSP00000363089	0.754
PPARG	IL1B	ENSP00000287820	ENSP00000263341	0.858
PPARG	IL6	ENSP00000287820	ENSP00000385675	0.82
PPARG	NFKB1	ENSP00000287820	ENSP00000226574	0.972
PPARG	STAT3	ENSP00000287820	ENSP00000264657	0.826
PPARG	TLR4	ENSP00000287820	ENSP00000363089	0.738
STAT3	ICAM1	ENSP00000264657	ENSP00000264832	0.832
STAT3	IL1B	ENSP00000264657	ENSP00000263341	0.938
STAT3	IL6	ENSP00000264657	ENSP00000385675	0.984
STAT3	JAK1	ENSP00000264657	ENSP00000499900	0.999
STAT3	NFAT5	ENSP00000264657	ENSP00000338806	0.748
STAT3	NFKB1	ENSP00000264657	ENSP00000226574	0.962
STAT3	NOS2	ENSP00000264657	ENSP00000327251	0.749
STAT3	PPARG	ENSP00000264657	ENSP00000287820	0.826
STAT3	TLR4	ENSP00000264657	ENSP00000363089	0.846
TICAM2	TLR4	ENSP00000415139	ENSP00000363089	0.999
TLR4	HSP90B1	ENSP00000363089	ENSP00000299767	0.988
TLR4	HSPA4	ENSP00000363089	ENSP00000302961	0.996
TLR4	ICAM1	ENSP00000363089	ENSP00000264832	0.768
TLR4	IL1B	ENSP00000363089	ENSP00000263341	0.986
TLR4	IL6	ENSP00000363089	ENSP00000385675	0.973
TLR4	NFKB1	ENSP00000363089	ENSP00000226574	0.993
TLR4	NOS2	ENSP00000363089	ENSP00000327251	0.754
TLR4	PPARG	ENSP00000363089	ENSP00000287820	0.738
TLR4	STAT3	ENSP00000363089	ENSP00000264657	0.846
TLR4	TICAM2	ENSP00000363089	ENSP00000415139	0.999

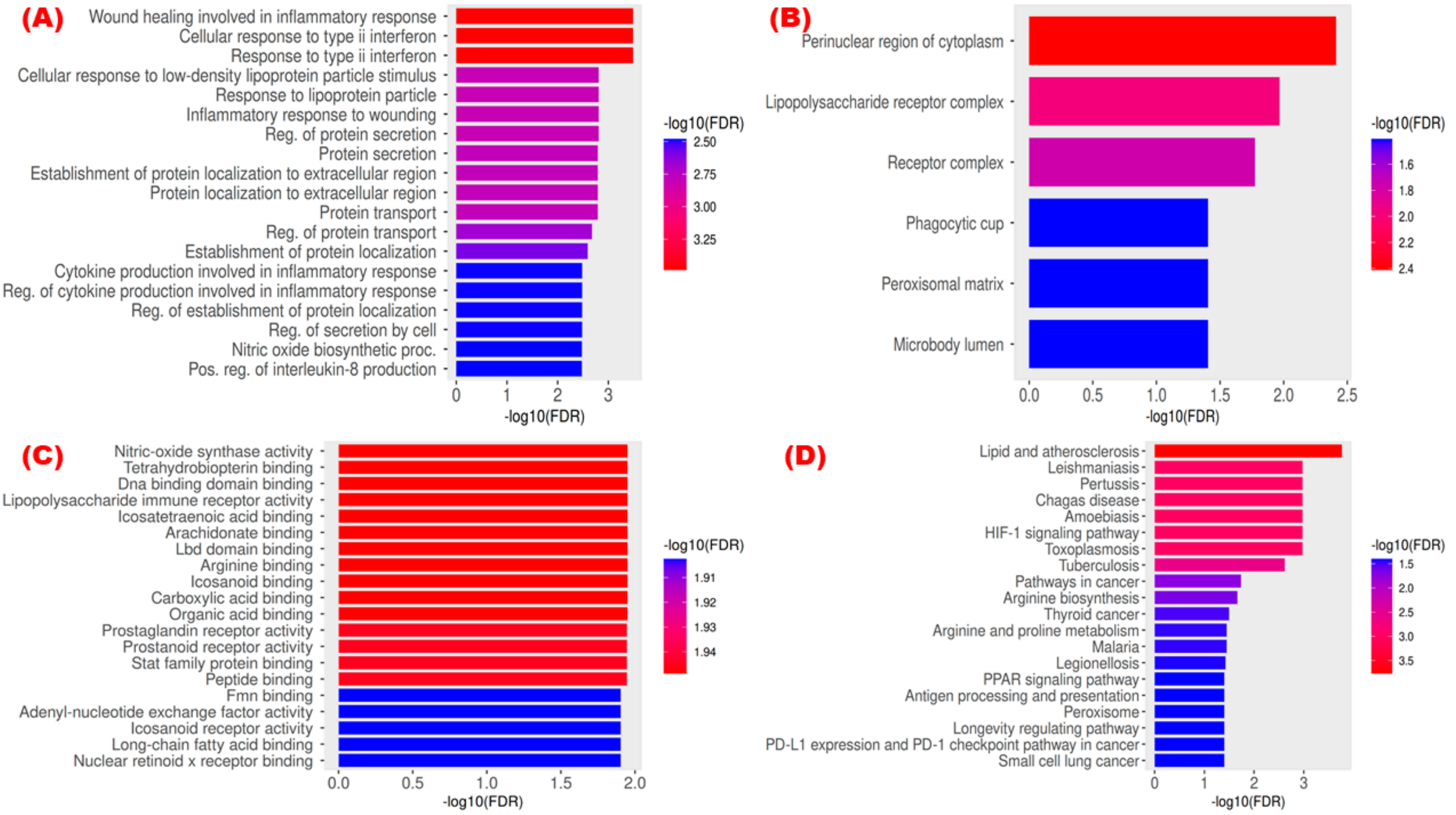

Figure S11. GO ontology analysis of Hub genes/proteins; (A) Biological processes (BP), (B) Cellular components (CC) and (C) Molecular functions (MF) & (D) KEGG significant pathways.

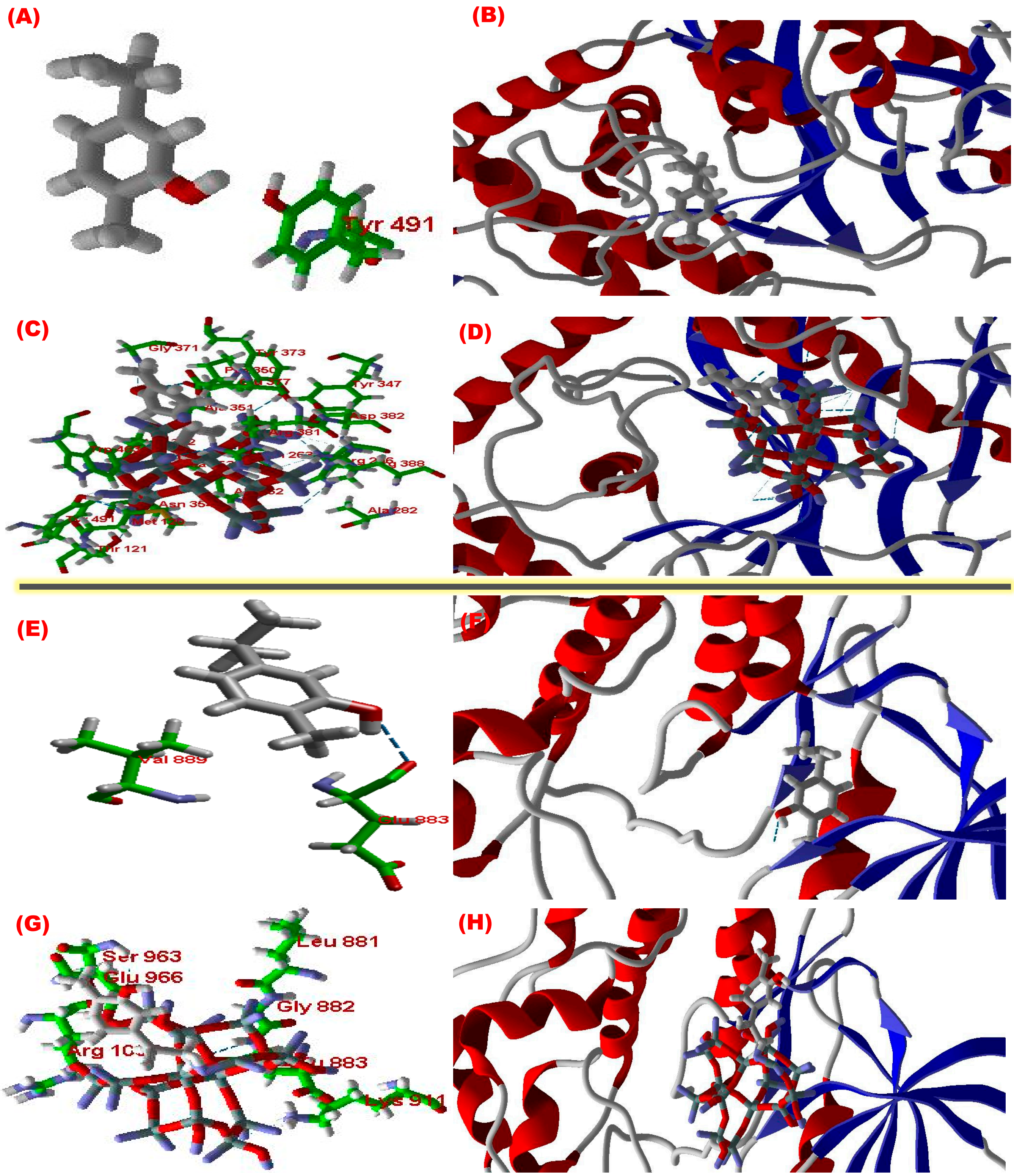
(A)
Tyr 491
(B)
(C)
Gly 371
Tyr 373
Tyr 347
Asp 382
Arg 381
Ala 282
Asn 354
Thr 121
(D)
(E)
Val 889
Glu 883
(G)
Ser 963
Glu 966
Leu 881
Gly 882
(H)

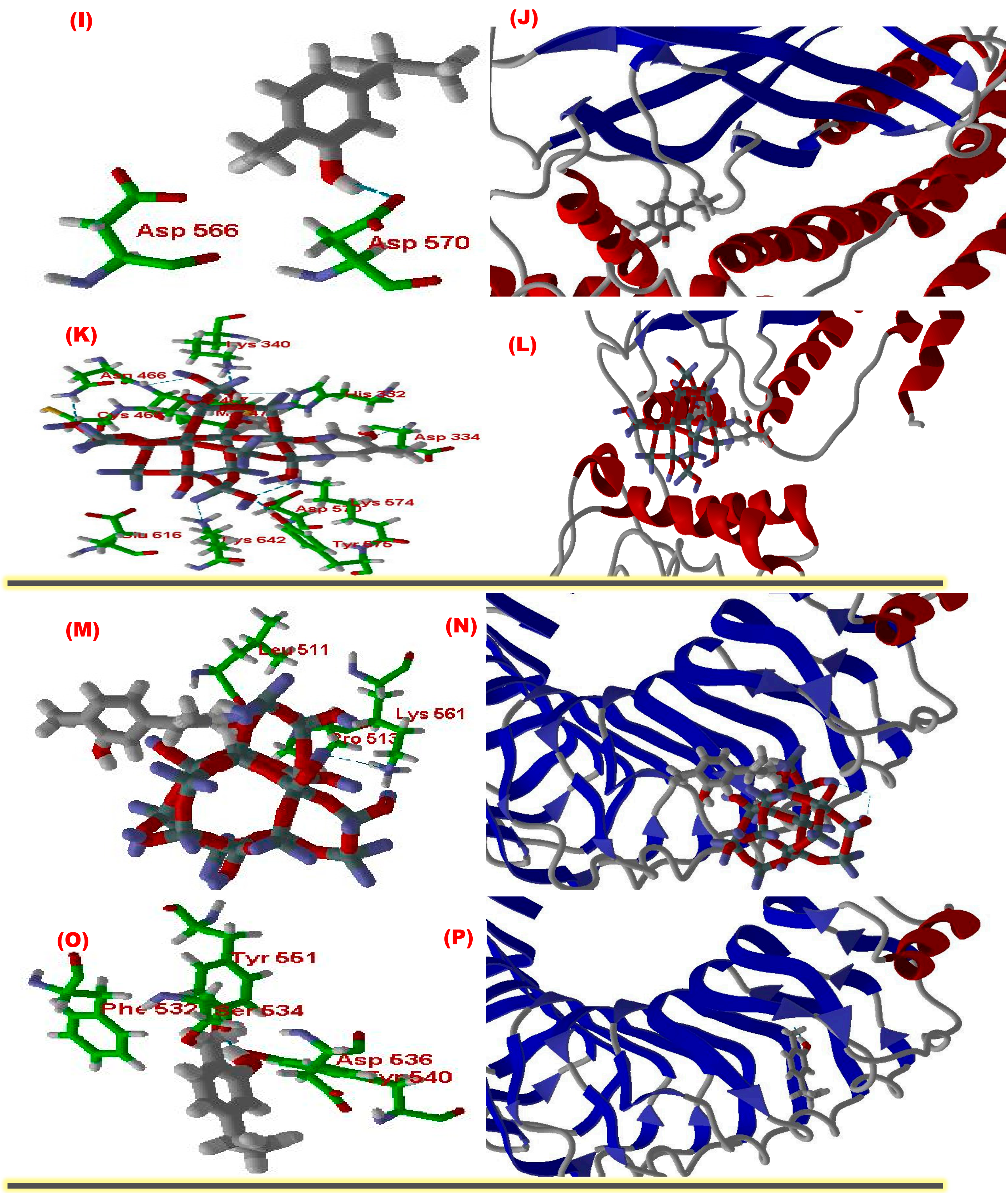

(I)
Asp 566
Asp 570
(J)
(K)
Lys 340
Asn 466
Asp 334
Lys 574
(L)
(M)
Leu 511
Lys 561
(N)
(O)
Tyr 551
Asp 536
(P)

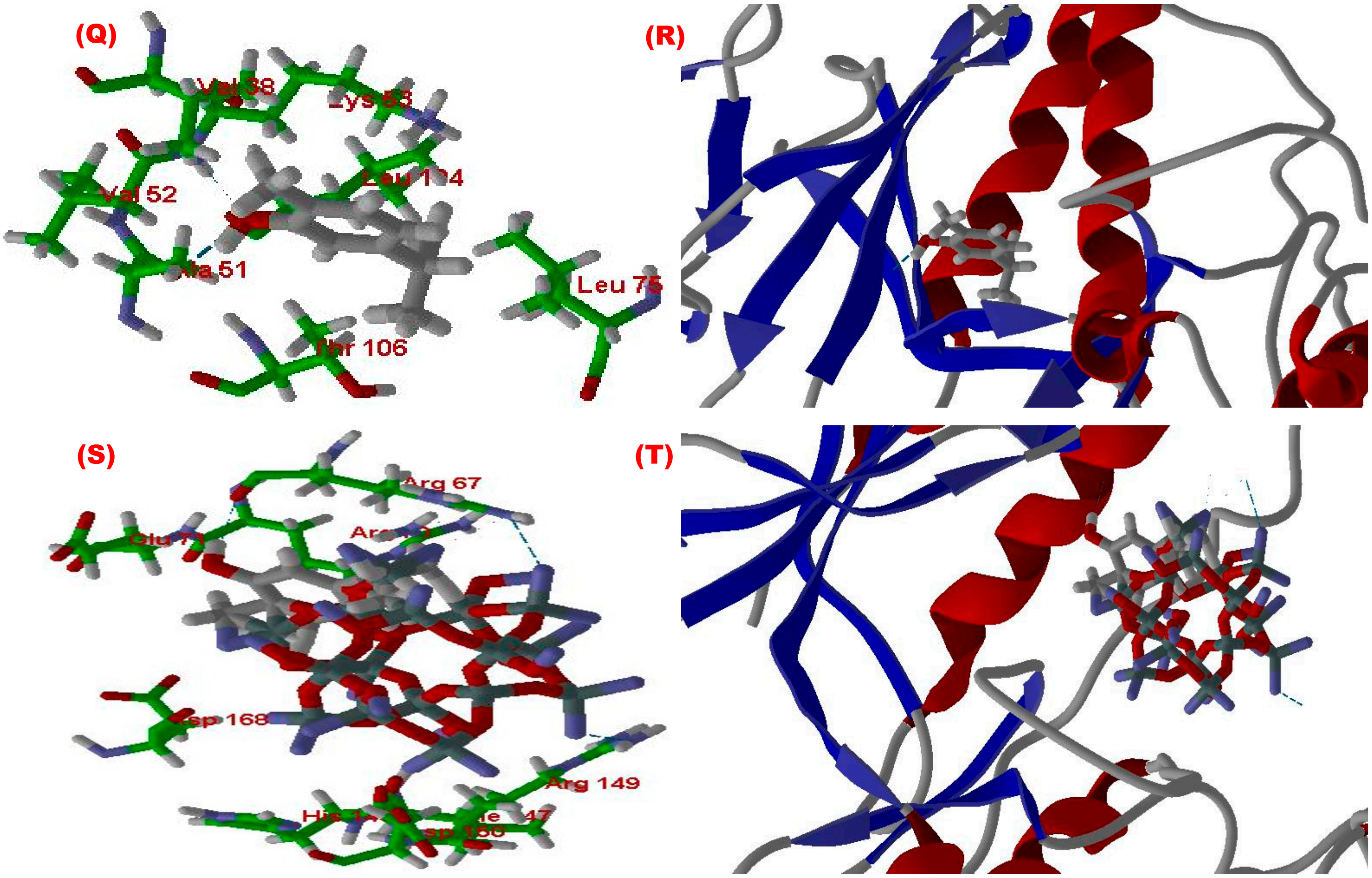

Figure S12. Molecular docking of free CV and CV-MSN with (A-D) iNOS (PDB ID: 4NOS), (E-H) JAK-1 (PDB ID: 3EYG), (I-L) STAT-3 (PDB ID: 3CWG), (M-P) TLR4 (PDB ID: 3FXI) and (Q-T) p38-MAPK (PDB ID: 3ZSG). Ligand map and 3D interaction image with the active sites.

Table S6. Global reactivity descriptors of Free CV and CV-MSN calculated by DFT using B3LYP/6-31G basis set.

Compound	HOMO(eV)	LUMO(eV)	ΔE(eV)	η(eV)	S (eV^{-1})	μ (eV)	χ	N(eV)
Free CV	-7.30	-4.60	2.70	1.35	0.37	-5.95	5.95	1.94
CV-MSN	-7.01	-6.86	0.15	0.072	6.67	-6.94	6.94	2.23

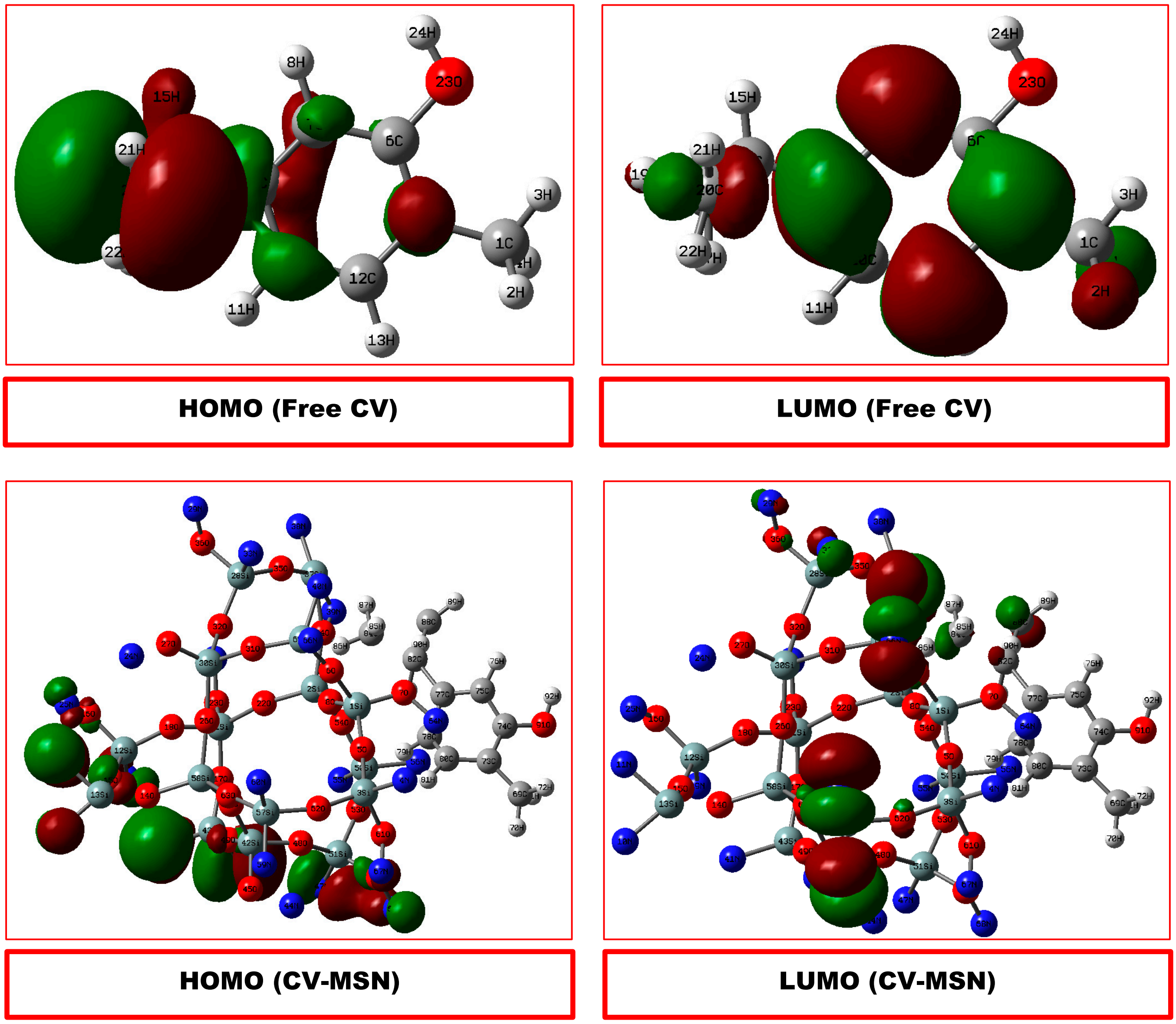

Figure S13. Visualization of HUMOs and LUMOs of free CV and CV-MSN determined using DFT calculation with B3LYP/6-31G

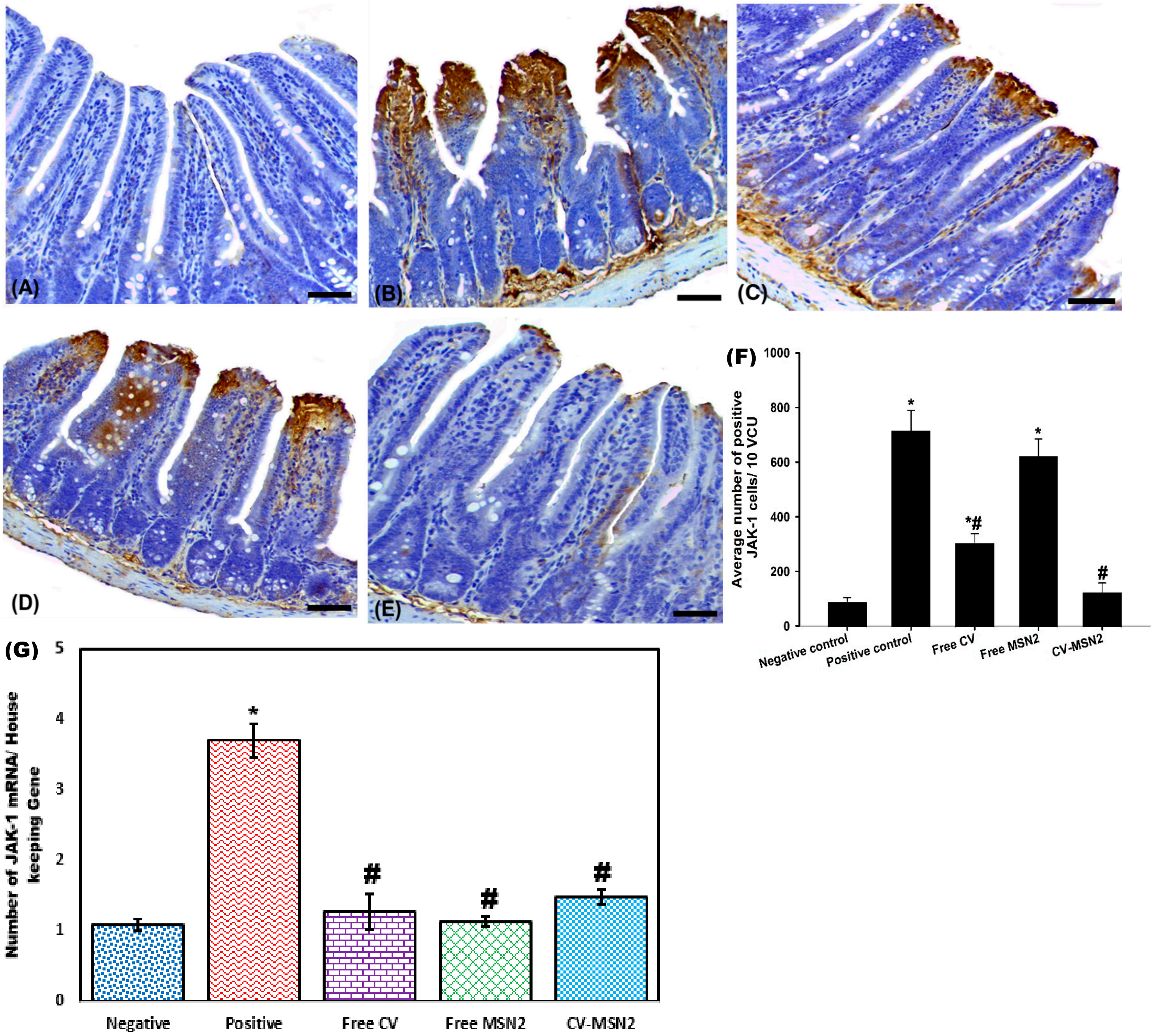
(A)
(B)
(C)
(D)
(E)
(F)
Average number of positive JAK-1 cells/ 10 VCU
0
200
400
600
800
1000
*
*#
*
#
Negative control
Positive control
Free CV
Free MSN2
CV-MSN2
(G)
Number of JAK-1 mRNA/ House keeping Gene
0
1
2
3
4
5
*
#
#
#
Negative
Positive
Free CV
Free MSN2
CV-MSN2

Figure S14. IHC photographs of cytoplasmic JAK-1 protein expression in ileal tissue of *C. parvum* infected mice. **(A)** Negative control. **(B)** Positive control. **(C)** Free CV. **(D)** Free MSN2. **(E)** CV-MSN2 treated groups. Scale bar = 100 µm. **(F)** IHC scoring of JAK-1 positive staining cells. **(G)** JAK-1 gene expression determined by qRT-PCR. ELISA results of IL-6 (H). Data are shown as means ± SD. *Significant at P< 0.05 against Negative control. #Significant at P< 0.05 against Positive control.

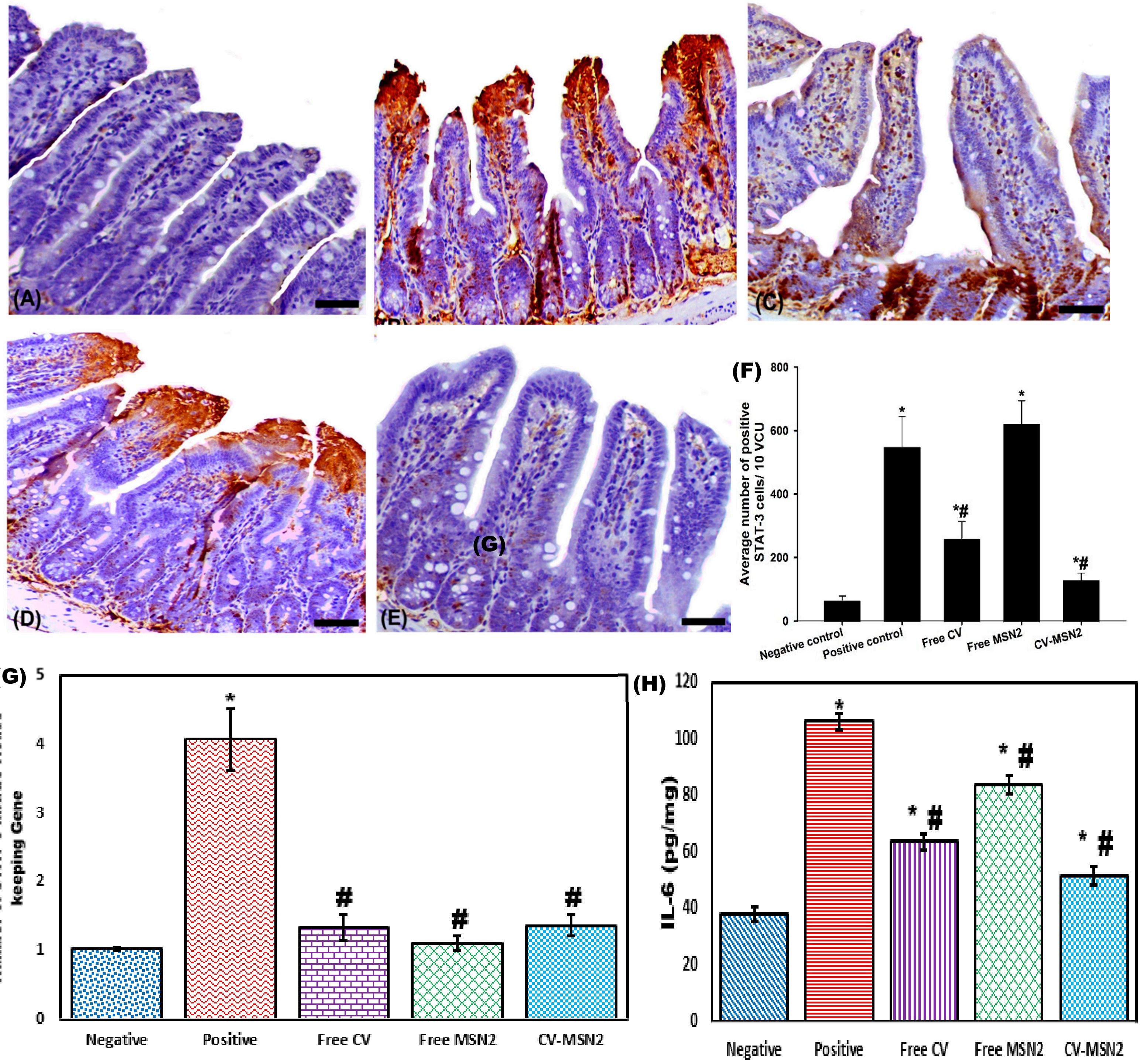

Figure S15. IHC photographs of cytoplasmic and nuclear STAT-3 protein expression in ileal tissue of *C. parvum* infected mice. **(A)** Negative control. **(B)** Positive control. **(C)** Free CV. **(D)** Free MSN2. **(E)** CV-MSN2 treated groups. Scale bar = 100 µm. **(F)** IHC scoring of STAT-3 positive staining cells. **(G)** STAT-3 gene expression determined by qRT-PCR. ELISA results of IL-6 **(H)** Data are shown as means ± SD. *Significant at P< 0.05 against Negative control. #Significant at P< 0.05 against Positive control.

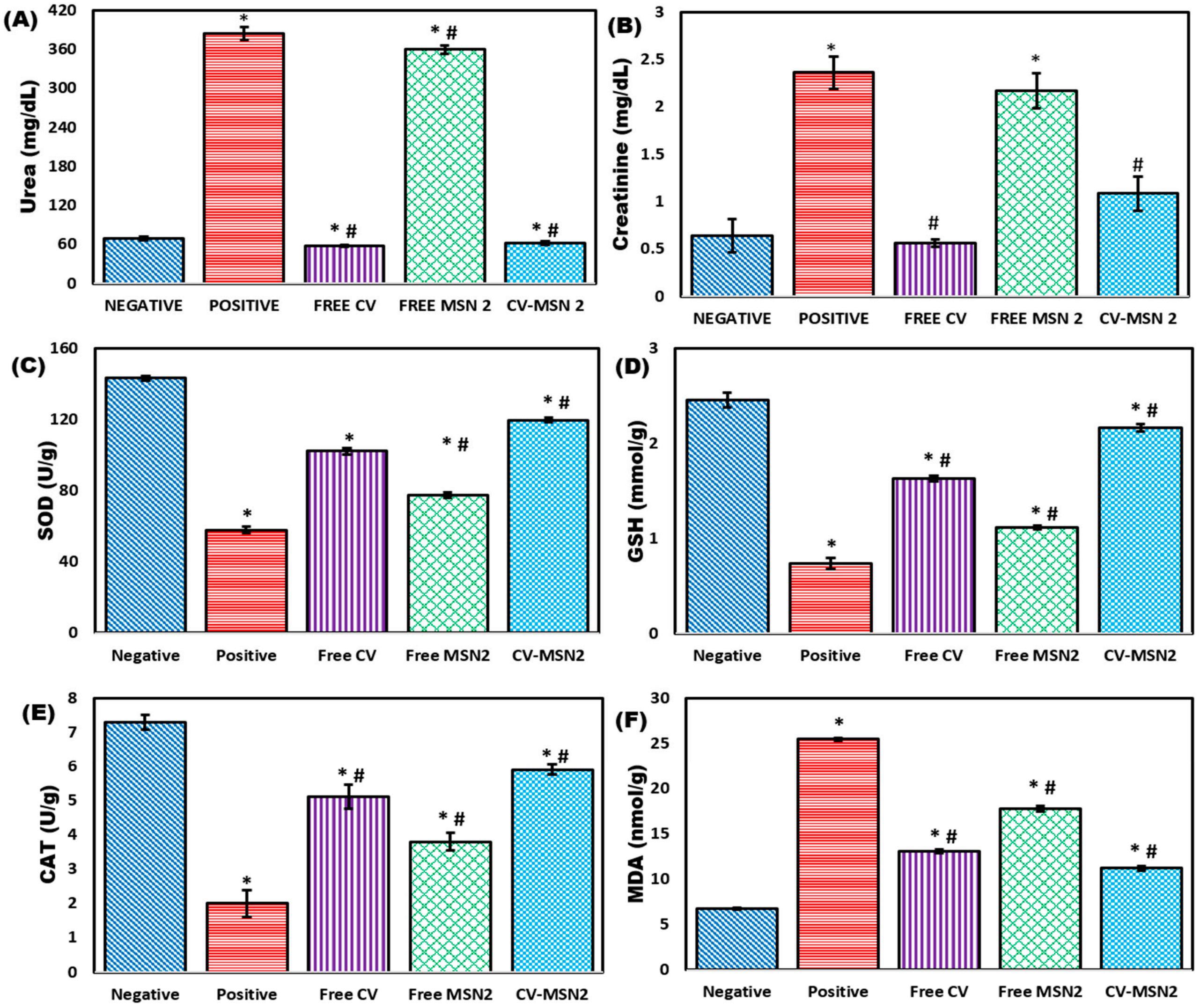

Figure S16. Kidney functions analysis. **(A)** Urea, and Creatinine **(B)** & Oxidative stress biomarkers determined by UV-vis spectrophotometer. SOD **(C)**, GSH **(D)**, CAT **(E)**, and MDA **(F)**. Data are shown as means ± SD. *Significant at P< 0.05 against Negative control. #Significant at P< 0.05 against Positive control.
